# Co-targeting CDK2 and CDK9 inhibits MYC oncogenic programs and has anti-tumor effects in *CIC*-rearranged sarcoma

**DOI:** 10.1038/s43856-026-01956-1

**Published:** 2026-10-07

**Authors:** Quentin Odom-Lewis, Samantha L. Davis, Stephan Pieper, Shannon M. R. Legge, Emilia F. A. Regenfuss, Nicolas Fernandez, Phillip D. Michaels, João A. Paulo, Steven P. Gygi, Chen Chu, Shanshan Zheng, Paola Dal Cin, Jason L. Hornick, Nicole L. Solimini, Timothy B. Branigan, Mohamed Alyousef, Emanuele Mazzola, Ewa T. Sicinska, Karen Bui, Prafulla C. Gokhale, Christopher A. French, George D. Demetri, Geoffrey I. Shapiro, Inga-Marie Schaefer

**Affiliations:** 1 Department of Pathology, Brigham and Women’s Hospital, Harvard Medical School, Boston, MA USA; 2 Broad Institute of MIT and Harvard, Cambridge, MA USA; 3 Department of Cell Biology, Harvard Medical School, Boston, MA USA; 4 Department of Cancer Biology, Dana-Farber Cancer Institute, Boston, MA USA; 5 Department of Genetics, Blavatnik Institute, Harvard Medical School, Boston, MA USA; 6 Department of Medical Oncology, Experimental Therapeutics Core, Dana-Farber Cancer Institute, Harvard Medical School, Boston, MA USA; 7 Center for Sarcoma and Bone Oncology, Dana-Farber Cancer Institute, Boston, MA USA; 8 Ludwig Center at Harvard, Boston, MA USA; 9 Department of Data Science, Dana-Farber Cancer Institute, Boston, MA USA; 10 Department of Pathology, Dana-Farber Cancer Institute, Harvard Medical School, Boston, MA USA; 11 Department of Medicine, Brigham and Women’s Hospital, Harvard Medical School, Boston, MA USA

**Keywords:** Sarcoma, Cancer genetics, Targeted therapies

## Abstract

**Background:**

*CIC*-rearranged sarcoma is a rare and highly aggressive cancer that primarily affects children and young adults and has few effective treatment options. Previous studies have identified molecular dependence on cyclin-dependent kinase 2 (CDK2), but additional therapeutic vulnerabilities remain poorly defined. Here, we investigate the activity of CDK2-targeted therapies and evaluate combination strategies using new patient-derived models of *CIC*-rearranged sarcoma.

**Methods:**

We established patient-derived cell lines and matching xenograft models from tumors harboring *CIC::DUX4* and *CIC::NUTM1* fusions, with xenograft studies performed in 7-week-old female NSG mice. We assessed the effects of CDK2 inhibition alone and in combination with cyclin-dependent kinase 9 (CDK9) inhibition using molecular, functional, and transcriptomic analyses in vitro and in vivo.

**Results:**

Here we show that *CIC*-rearranged sarcoma depends on the MYC oncogene and is vulnerable to CDK2 inhibition. The CDK2 inhibitor tagtociclib suppresses MYC-regulated gene expression, inhibits tumor cell proliferation, and reduces tumor growth in patient-derived models. Combined inhibition of CDK2 and CDK9 produces synergistic anti-tumor activity by further suppressing MYC-driven transcription, enhancing apoptotic cell death, and improving tumor control in vitro and in vivo.

**Conclusions:**

These findings identify MYC-driven transcription as a therapeutic vulnerability in *CIC*-rearranged sarcoma and support combined CDK2 and CDK9 inhibition as a promising treatment strategy. The patient-derived models established in this study also provide valuable resources for future preclinical and translational research.

## Plain Language Summary

*CIC*-rearranged sarcoma is a rare and aggressive cancer that mainly affects children and young adults. There are currently no effective treatments for this disease. To help find new treatment options, we created laboratory models from patients’ tumors, including the first model of a rare form called *CIC::NUTM1* sarcoma. Using these models, we discovered that the cancer relies on a protein called MYC, which helps cancer cells grow and survive. We found that two enzymes, CDK2 and CDK9, keep MYC active. When we used drugs to block both enzymes at the same time, the treatment worked much better than blocking either one alone. The drug combination stopped cancer cells from growing, switched off key cancer-driving processes, and caused tumor cells to die in laboratory experiments and in mice. These findings suggest that targeting CDK2 and CDK9 together could be a promising new treatment approach that should be tested in clinical trials for people with *CIC*-rearranged sarcoma.

## Abstract (web summary)

Odom-Lewis et al. identify CDK2 and CDK9 as actionable dependencies across multiple *CIC-*rearranged sarcoma subtypes, including *CIC::NUTM1*, using newly developed patient-derived xenograft and cell line models. Their preclinical data suggest that combining CDK2 inhibition with therapies that indirectly suppress MYC expression may enhance antitumor activity while reducing the toxicity associated with less selective CDK inhibitors.

## Introduction

*CIC*-rearranged sarcoma is a rare and aggressive mesenchymal neoplasm that affects patients over a wide age range (6–81 years) with a median age of 30 years and ~20% of cases affecting children^1,2^. These sarcomas predominantly arise within soft-tissue (mostly trunk and extremities), with subsets occurring in bone or at visceral sites. Previously misdiagnosed as a type of Ewing sarcoma lacking *EWSR1* rearrangement, it was discovered in 2006 that this sarcoma harbors *capicua* (*CIC)* rearrangement^3^, leading to its recognition as a distinct entity in the 2020 WHO Classification of Tumors^4^. While 95% of *CIC*-rearranged sarcomas are driven by a *CIC::DUX4* fusion^5^, rare cases harbor alternate fusions of *CIC* with either *NUTM1*^6,7^, *NUTM2A*^8^, *FOXO4*^9^, or *LEUTX*^10^. Our group had previously described, for the first time, a sarcoma with a *CIC::NUTM1* fusion in an adult patient^6^, which has subsequently been reported in a smaller case series^7^. In general, *CIC*-rearranged sarcoma shows very aggressive clinical behavior with frequent metastases that most commonly affect the lung^1,2^. A median overall survival of 12 months and poor response to systemic therapies developed for Ewing sarcoma (such as the VDC/IE regimen comprising vincristine, Adriamycin/doxorubicin, cyclophosphamide, ifosfamide, and etoposide) and/or radiation^1,2,11^ highlights the urgent need to generate a better understanding of the oncogenic mechanisms of *CIC*-rearranged sarcoma and identify biologic vulnerabilities for therapeutic targeting in this disease, for which no effective therapies currently exist.

The lack of in vitro and in vivo models that reliably exhibit the biologic and genetic characteristics observed in patients’ tumors has largely prevented therapeutic advances for patients with *CIC*-rearranged sarcoma. While some groups have recently established cell lines or in vivo models of *CIC::DUX4*-rearranged sarcoma, models harboring alternative fusions such as *CIC::NUTM1* have been lacking. The development of representative preclinical models spanning the molecular spectrum of *CIC*-rearranged sarcoma is therefore essential to improve our understanding of disease biology and facilitate the identification of new therapeutic strategies.

Genomic analysis of *CIC::DUX4*-rearranged sarcoma demonstrated that these tumors harbor frequent trisomy 8 and/or *MYC* amplification, revealing a potentially targetable oncogenic pathway^12^. The recent development of other preclinical patient-derived models driven by *CIC::DUX4* fusion, including the NCC-CDS1-X1-C1 and NCC-CDS2-C1 cell lines, has allowed drug screening to identify bortezomib and crizotinib, among others, as having anti-tumor activity^13,14^.

CDK2-cyclin E was recently identified as a potential druggable target in *CIC*-rearranged sarcoma, with the pan-CDK inhibitor dinaciclib suppressing tumor growth and metastasis in vivo^15^. CDK2 belongs to the cyclin-dependent kinase family of Ser/Thr protein kinases. It binds cyclin E and regulates the transition from G1 to S phase; binding of CDK2 to cyclin A coordinates S phase progression. CDK2 can phosphorylate substrates involved in a range of physiological functions, including the regulation of histone modifications, chromatin, transcription, and RNA/DNA metabolism^16^. Based on these observations, we hypothesized that selective CDK2 inhibitors in clinical development for other solid tumors might have anti-tumor effects in *CIC*-rearranged sarcoma.

Here, we establish patient-derived cell lines and corresponding xenograft models of *CIC*-rearranged sarcoma, including the first reported models harboring a *CIC::NUTM1* fusion, and use these together with previously described models to evaluate CDK2-targeted therapeutic strategies. We show that *CIC*-rearranged sarcoma depends on MYC-driven transcriptional programs that are vulnerable to CDK2 inhibition and that combined inhibition of CDK2 and CDK9 synergistically suppresses tumor growth by enhancing apoptotic cell death in vitro and in vivo. These findings identify MYC-driven transcription as a therapeutic vulnerability in *CIC*-rearranged sarcoma, provide new preclinical models for future studies, and support combined CDK2 and CDK9 inhibition as a promising therapeutic strategy.

## Methods

### Tumor and tissue samples

Frozen, discarded, deidentified tumor specimens were obtained from patients undergoing clinically indicated surgical procedures at Brigham and Women’s Hospital and banked at Brigham and Women’s Hospital. Paraffin-embedded and formalin-fixed (FFPE) tissue samples were retrieved from the Brigham and Women’s Hospital surgical pathology archives.

### Cell lines

The CIC01 and CIC02 cell lines were established in the Schaefer laboratory from cryopreserved tumor tissue originally obtained from patients undergoing clinically indicated surgery at Brigham and Women’s Hospital under the IRB-approved protocols described below. Tumor tissue was transferred to the Sicinska laboratory at Dana-Farber Cancer Institute for establishment of patient-derived xenograft (PDX) models, and residual cryopreserved tumor tissue was subsequently used to establish the CIC01 and CIC02 cell lines. Cryopreserved tumor tissue was mechanically disaggregated using 100 µL collagenase (200 mg/mL), followed by overnight incubation. Cell clusters were subsequently mechanically disaggregated, filtered through a cell strainer, centrifuged, and plated into a T25 flask. Cell cultures were continuously maintained in regular growth media for 15 passages until they were deemed immortalized cell lines. CIC01 and CIC02 were validated against the initial biopsy material by sequencing verification of known unique gene mutations. NCC-CDS1-X1-C1 (CDS1) and NCC-CDS2-C1 (CDS2) cells were established by Dr. Tadashi Kondo, National Cancer Center Research Institute, Tokyo, Japan, and shared by Dr. Ross Okimoto, Department of Medicine, and Helen Diller Family Comprehensive Cancer Center, UCSF, San Francisco. Daudi (cat. no. CCL-213) and U2-OS cells (cat. no. 50-238-4124) were obtained from ATCC. All cultures were mycoplasma-free.

### Cell culture and reagents

The CIC01 and CIC02 cell lines were maintained in IMDM medium (Gibco, cat no. 12440-061) supplemented with 15% and 10% fetal bovine serum, respectively (FBS; Gemini Bio, cat no. 100-106-500), antibiotic antimycotic solution (Gemini Bio, cat no. 400-101-100), L-glutamine (Corning, cat no. 25-005-CL), gentamycin (Gibco, cat no. 15750060), and amphotericin B (Gibco, cat no. 15290-029). CDS1 and CDS2 cells were grown in RPMI 1640 medium (Gibco, cat no. 11875-119) supplemented with 15 and 10% FBS, respectively, containing antibiotic antimycotic solution, L-glutamine, gentamycin, and amphotericin B. Daudi cells were grown in RPMI 1640 medium supplemented with 10% FBS, antibiotic antimycotic solution, L-glutamine, gentamycin, and amphotericin B. Tagtociclib (PF-07104091) was obtained from Selleck Chemicals (cat. no. S9878), BLU-222 from MedChem Express (cat. no. HY-160701), PF-06873600 from Selleck Chemicals (cat. no. S8816), palbociclib from LC Laboratories (cat. no. P7788), KB-0742 from MedChem Express (cat. no. HY-137478A), AZD4573 from Selleck Chemicals (cat. no. S8719), 10058-F4 from MedChem Express (cat. no. HY-12702), and 10074-G5 from MedChem Express (cat. no. HY-100996). Cycloheximide was obtained from Cell Signaling Technology (cat. no. 2112).

### Xenograft models

The CIC01 and CIC02 PDX models were established in the Sicinska laboratory at Dana-Farber Cancer Institute from tumor specimens obtained from patients with *CIC*-rearranged sarcoma undergoing clinically indicated surgery at Brigham and Women’s Hospital. Cryopreserved tumors were implanted subcutaneously into 7-week-old female NSG (NOD.Cg-*Prkdc*^*scid*^ *Il2rg*^*tm1Wjl*^/SzJ; strain #005557) mice obtained from The Jackson Laboratory. Briefly, mice were anesthetized with inhaled isoflurane and a small incision was made in the flank region of the mouse. A tumor fragment, approximately 2–3 mm dipped in Matrigel was placed in the subcutaneous space and the wound closed with staples. Topical ropivacaine was applied intra-incisional, and staples were removed after 7 days. For drug treatment studies, singly engrafted mice were enrolled into treatment groups when tumors reached an average tumor volume of 70–80 mm^3^ in size, as measured by calipers, and determined by the tumor volume equation: volume = long diameter × short diameter^2^ × 0.5. Mice were randomly assigned to treatment groups using Studylog software (Studylog Systems, Inc.) and received tagtociclib (120 or 150 mg/kg oral gavage twice daily for 28 days; formulated in 0.5% methylcellulose A4M and 0.1% Tween 80 in water), AZD4573 (10 mg/kg IP twice daily 2 h apart (Q2H) on a 2ON 5OFF schedule; formulated in 2% DMA, 30% PEG400, 1% Tween 80 in water) or vehicle control. No animals died from drug treatment. No formal a priori sample size calculation was performed; at least three animals per group were planned, with tumor volume as the primary outcome measure. Animals with growing tumors were eligible for enrollment, and no animals or data points were excluded from the analyses. Animals in each treatment group were housed in separately labeled cages, and treatment order was maintained throughout the study. The study was not blinded; however, the animal technician performing the experiments was agnostic to the expected study outcome. Animals were monitored daily for signs of distress and tumor growth and were euthanized when predefined humane endpoints were reached, including tumor volumes exceeding 2000 mm^3^ or if the tumors became ulcerated/necrotic. Tumors were dissected and fixed in 10% formalin for corollary studies, including hematoxylin and eosin staining and IHC of sectioned tumors. Sections (4 μm) were cut from fixed and embedded tumors and stained with Ki67 and pRb Ser 807/811. Immunoblotting studies were performed as described above. Mice were housed in OptiMice cages and rack system (Animal Care Systems) in rooms with a 12-h day/night cycle and temperature and humidity controlled at 72 ± 2 °F and 35–55% relative humidity. All procedures were conducted under protocols approved by the Institutional Animal Care and Use Committee at the Dana-Farber Cancer Institute.

### Immunohistochemistry

IHC was performed on 4-µm-thick formalin-fixed paraffin-embedded TMA sections following pressure cooker antigen retrieval (Target Retrieval Solution; pH 6.1; Dako) using antibodies listed in Supplementary Table S4 as well as controls and staining conditions as previously described in ref. ^17^.

### Fluorescence in situ hybridization

Fluorescence in situ hybridization (FISH) was performed on 5-µm-thick formalin-fixed paraffin-embedded tissue sections, which were de-paraffinized and digested using methods described previously^18^. For *CIC* break-apart FISH, a *CIC* break-apart probe (Empire Genomics) was hybridized to tissue sections following manufacturer’s directions. For chromosome 8 copy number, a chromosome 8 centromere probe (Abbott Molecular) was hybridized to tissue sections following manufacturer’s directions. Briefly, tissues and probes were co-denatured at 80 °C, hybridized at least 16 h at 37 °C in a darkened, humid chamber, washed in 2× SSC at 71 °C for two minutes, rinsed in room temperature (RT) 2× SSC, and counterstained with DAPI (4’,6-diamidino-2-phenylindole, Abbott Molecular/Vysis). Slides were imaged using an Olympus AX51 fluorescence microscope. Individual images were captured using an Applied Imaging system running CytoVision Genus version 7.5. *CIC* and centromere 8 status was assessed in 50 nuclei per sample.

### PCR and Sanger sequencing analysis

Genomic PCR of the *CIC::NUTM1* and *CIC::DUX4* fusion breakpoints was performed using primers with the sequences CTGAGTTGCCTGAGTTTCGG (*CIC* exon 18 + 39 F), cccctaatccagtccccatt (*NUTM1* intron 5 + 287 R), CCTCCAAGGTTTGTCTGCAG (*CIC* exon 21 + 46 F), GGAAAGCGATCCTTCTCAAA (*DUX4* exon 1 + 356 R), and Platinum Hot Start 2× PCR Master Mix (Invitrogen/Thermo Fisher, cat no. 13000-013) with analysis by Sanger sequencing.

### Targeted sequencing

Targeted sequencing was performed using the OncoPanel platform as described before^19,20^, interrogating the exonic sequences of 447 cancer-associated genes for mutations and copy number variations, and 191 introns across 60 genes for gene rearrangements. DNA was extracted from FFPE or frozen tissue using a QIAamp DNA Mini Kit (Qiagen). Construction of hybrid-capture libraries, sequencing using the Illumina NovaSeq 6000 (Illumina), and sequence data analysis was performed as previously described in ref. ^19^. All detected alterations were reviewed manually and annotated.

### Protein blotting

Whole cell lysates were prepared as previously described in ref. ^21^. Protein concentrations were determined using the Bio-Rad protein assay (Bio-Rad Laboratories, cat. no. 5000006). Electrophoresis, immunoblotting, and chemiluminescence detection were performed as described previously^21^ using antibodies listed in Supplementary Table S4. For the detection of >200 kDa fusion oncoproteins, conditions were modified to include the use of NuPAGE 3-8% Tris-Acetate gel (Thermo Fisher Scientific, cat no. EA0375BOX) and NuPAGE Tris-Acetate SDS running buffer (Thermo Fisher, cat. no. LA0041), followed by 16-h protein transfer at 30 V.

### Mass spectrometry

Digested and gel-extracted peptides were desalted using StageTip before LC-MS/MS. Data for CIC01 and CIC02 were collected using an Exploris 480 mass spectrometer coupled with a Proxeon 1200 liquid chromatograph (Thermo Fisher Scientific). Peptides were separated on a 100-μm inner diameter column packed with ~35 cm of Accucore C18 resin (2.6 μm, 150 Å). Peptides were separated using a 90-min gradient of 5 to 30% acetonitrile in 0.125% formic acid at 320 nL/min. Data for CDS1 and CDS2 were collected using an Orbitrap Astral mass spectrometer coupled with a Neo Vanquish liquid chromatograph (ThermoFisher Scientific). Peptides were separated on a 110-cm uPAC C18 column using a 60-min gradient of 5–29% acetonitrile in 0.125% formic acid at 300 nL/min. The scan sequence began with an Orbitrap MS^1^ spectrum and the top twenty precursors were selected for MS^2^ analysis. Mass spectra were processed using a Comet-based in-house software pipeline. Database searching included all entries from the human UniProt database, concatenated with database of all protein sequences in reversed order. Searches were performed using a 50-ppm precursor ion tolerance and a product ion tolerance of 0.03 Th. Carbamidomethylation of cysteine residues (+ 57.0215 Da) was set as static, while oxidation of methionine residues (+ 15.9949 Da) was set as a variable modification. Peptide-spectral matches were altered to a 1% FDR^22,23^ and filtered using a linear discriminant analysis as described previously^24^, and then further collapsed to a final protein-level FDR of 1%. Furthermore, protein assembly produced the smallest set of proteins necessary to account for all observed peptides. Spectral counts were extracted, and the data were subsequently analyzed.

### Cell proliferation assays

Cells were plated in 96-well plates at 5000 cells/well (CIC02 and CDS2), 10,000 cells/well (CIC01 and Daudi), or 15,000 cells/well (CDS1) in growth medium and incubated overnight. Drug treatment was performed for 48 h. BrdU was then added to the cells for 24 h. BrdU incorporation, fixation, and detection were performed using a BrdU Cell Proliferation ELISA according to the manufacturer’s protocol (Roche Diagnostics). BrdU incorporation was presented as fold change of the DMSO control. All cell response assays were performed in triplicate.

### Cell viability and apoptosis assays

Cells were plated in 96-well plates at 5000 cells/well (CIC02 and CDS2), 10,000 cells/well (CIC01 and Daudi), or 15,000 cells/well (CDS1) in growth medium and incubated overnight. Cell viability studies were performed after adding MYC inhibitors for 3 days or tagtociclib for 3 (Daudi) or 6 days (CIC01, CIC02, CDS1, and CDS2) versus the DMSO-control using a CellTiter-Glo luminescence assay (Promega). Apoptosis studies were performed after 48 h of treatment using the Caspase-Glo 3/7 luminescence assay (Promega).

### BrdU incorporation assay

The cells were pulsed with BrdU (10 µM; BD Biosciences) for 2 h, trypsinized, washed with phosphate-buffered saline (PBS), and fixed with 100% ethanol (EtOH) overnight at 4 °C. Next, the DNA was hydrolyzed by incubation with 2 N HCl/0.5% Triton ×-100 for 30 min at RT and neutralized by adding 0.1 M Na2B4O7 (pH 8.5). Subsequently, the cells were incubated in 100 µL of PBS/1% bovine serum albumin containing fluorescein isothiocyanate-conjugated anti-BrdU antibody (BD Biosciences, cat. no. 556028) 1:5 for 30 min at RT. Next, the cells were washed twice with PBS, stained with 20 µL of 7-AAD (7-Amino-Actinomycin D) (BD-Biosciences), resuspended in PBS, and analyzed by flow cytometry. Data acquisition was performed using a BD LSRFortessa Flow Cytometry Analyzer, and data analysis was performed using FlowJo (v10.8.1).

### Growth over time analysis and cell segmentation

Cells were plated in regular growth media at 2 mio cells/well in 6-well plates and treated with inhibitor or DMSO control starting one day after plating (day 0). Culture images were obtained using a Spot RT Slider Camera and Spot Software (Version 4.6 for Windows) and a Nikon Eclipse TE2000-S inverted microscope. The cells were trypsinized and counted on day 7 using an automated cell counter (TC20, Bio-Rad). Cell segmentation was performed using the Cellpose framework (v4.0.8; Python 3.10.19)^25^, incorporating the Segment Anything Model (SAM) via the Cellpose-SAM interface to improve boundary detection^26^. For standard samples, pre-trained cyto3^27^ and base models were initially applied. To address differences in cell morphology and confluence across samples, a custom segmentation model (CDS3) was developed using an iterative human-in-the-loop training approach^28^. Representative images were divided into non-overlapping 256 × 256-pixel tiles to increase feature density and improve local boundary detection. The CDS3 model was initialized with cyto3 weights and fine-tuned for 100 epochs in a GPU-accelerated training environment to capture the characteristic cell shapes and boundary features present in the dataset. To ensure high biological accuracy, all automated segmentation outputs underwent comprehensive manual curation. Using the Cellpose graphical user interface, each segmentation mask was visually inspected and manually corrected, redrawn, or removed where necessary. While the automated models, including CDS3, provided efficient initialization, the final datasets used for downstream analyses consisted exclusively of manually verified masks.

### IC_50_ calculations and drug synergy analysis

IC_50_ values were calculated using GraphPad Prism. Drug synergy testing was performed using SynergyFinder 3.0 (https://synergyfinder.fimm.fi/)^29^, which generated the Loewe synergy scores.

### RNA sequencing

RNA library preparation, sequencing reaction, and bioinformatic analysis were conducted at GeneWiz, LLC. after 48 h treatment with tagtociclib (200 nM) versus DMSO. Total RNA was extracted using a Qiagen RNeasy Plus Universal mini kit following the manufacturer’s instructions (Qiagen) and a sequencing library was prepared using the NEBNext Ultra II RNA Library Prep Kit for Illumina (NEB, Ipswich, MA, USA). The samples were sequenced using a 2 × 150 bp Paired End (PE) configuration and overall read amount per HiSeq lane of ~350 M, and trimmed reads were mapped to the Homo Sapiens GRCh38 (hg38) reference genome available on ENSEMBL using the STAR aligner v.2.5.2b. Unique gene hit counts were calculated using feature Counts from the Subread package v.1.5.2. Differential expression was performed using the PyDESeq2 library in Python. We first filtered the top 10,000 genes across three replicates of treated versus untreated cell lines. We then followed the PyDESeq2 workflow to fit size factors, fit dispersion, and calculate log-fold changes. A list of up and downregulated genes with an adjusted *P* value of <0.05, and absolute value log2 fold change of > 0.5 was generated. We used the interactive heatmap Python library, Clustergrammer2, to hierarchically cluster our samples and visualize the differentially expressed gene expression data. Enrichment analysis was performed using the web tool Enrichr to identify transcription factors that may be upstream of differentially expressed genes. The following links provide direct access to the Enrichr analyses:

CIC01_up: https://maayanlab.cloud/Enrichr/enrich?dataset=3629904cb513bb274e6d9393d1023a6f

CIC01_down: https://maayanlab.cloud/Enrichr/enrich?dataset=6f428684e72b2832f944b729aa2bd641

CIC02_up: https://maayanlab.cloud/Enrichr/enrich?dataset=a1d03d575565348cdf0f965bd5c35e58

CIC02_down: https://maayanlab.cloud/Enrichr/enrich?dataset=ffcddfdf1b4be77b219cc6d9bb20f27a

### Constructs and virus infection

Three *CDK2* sgRNA constructs inserted into a pLenti-CRISPR V2 empty vector were obtained from GenScript. Transfections using vsvg and psPAX2 (Addgene) packaging plasmids and lentiviral harvesting were performed as described previously^30^. Following transduction, cells were selected with 1.5 μg/mL puromycin starting on day 2 post-infection. Culture images were obtained using a Spot RT Slider Camera and Spot Software (Version 4.6 for Windows) and a Nikon Eclipse TE2000-S inverted microscope.

### Statistics and reproducibility

Statistical analyses were performed using GraphPad Prism and Stata IC15 software, and Wilcoxon rank-sum tests or, where appropriate, log-linear *F*-tests or mixed-effects analysis accounting for repeated measurements over time, were used to compare two data sets. All applicable statistical tests were two-sided unless otherwise indicated. Statistical significance was set at *P* ≤ 0.05, and *P* values were corrected for multiple comparisons using the Benjamini–Hochberg method when needed. All experiments except cell cycle phase analyses and cell segmentation were performed at least in triplicate.

### Human and animal ethics statement

Frozen, discarded, deidentified tumor specimens were obtained from Brigham and Women’s Hospital under a protocol approved by the Brigham and Women’s Hospital Institutional Review Board (Protocol No. 2019P001987). The Institutional Review Board determined that informed consent was not required and granted a waiver of consent because the specimens were discarded and deidentified prior to analysis. Preclinical studies involving human tumor specimens and establishment of cell lines in the Schaefer laboratory were conducted under this protocol. Tumor banking and establishment of PDX models were performed under a research protocol approved by the Dana-Farber Cancer Institute Institutional Review Board (Protocol No. 05-434), with written informed consent obtained from participants. All human-subject research was conducted in accordance with applicable institutional policies, relevant guidelines and regulations, and the ethical principles governing research involving human participants. Animal studies were conducted at Dana-Farber Cancer Institute with approval from the Dana-Farber Cancer Institute Institutional Animal Care and Use Committee (Protocol No. 04-111) in an Association for Assessment and Accreditation of Laboratory Animal Care International-accredited vivarium. All animal procedures were performed in accordance with relevant institutional and national regulations for the care and use of laboratory animals. The study was conducted and reported in compliance with the ARRIVE guidelines.

## Results

### Copy number gains of *ETV4* and *MYC* are recurrent events in *CIC*-rearranged sarcoma

**Fig. 1:**
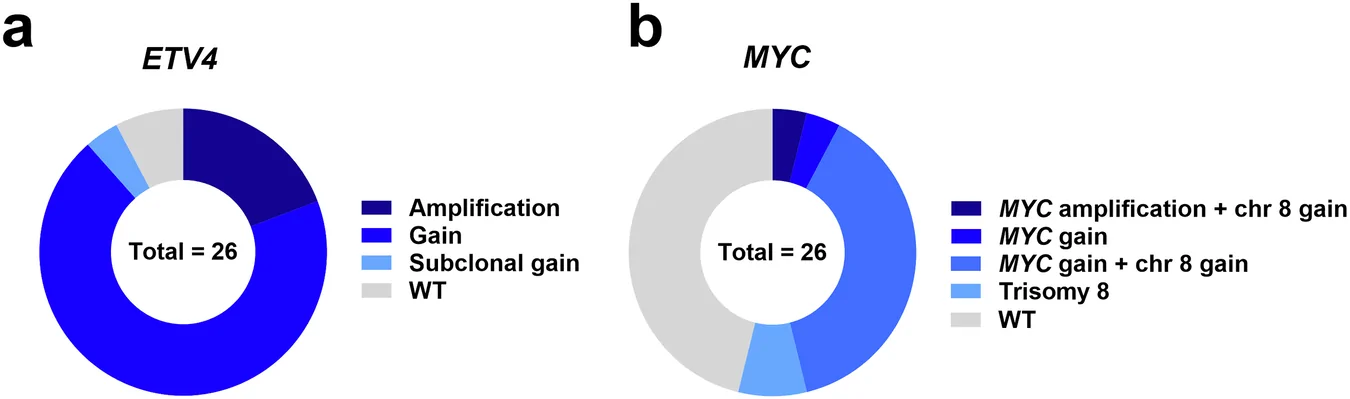
Copy number gains of *ETV4* and *MYC* are recurrent events in *CIC*-rearranged sarcoma. **a** NGS analysis in 26 clinical cases of *CIC*-rearranged sarcoma demonstrates genomic gain of *ETV4* in 24/26 cases, including amplification (*N* = 5), copy number gain (*N* = 18), and subclonal gain (*N* = 1). **b** NGS and FISH analysis reveal genomic gain of *MYC* in 14/26 cases, including amplification (*N* = 1), copy number gain (*N* = 11), and trisomy 8 (*N* = 2). *N* indicates biologically independent samples.

We performed targeted NGS in 26 clinical cases of FISH-proven *CIC*-rearranged sarcoma to determine the frequency of aberrations that indirectly or directly activate MYC, supporting a rationale for therapeutic targeting. FISH for *MYC* and centromere 8 was performed in a subset of two cases. The 26 patients comprised 9 males and 17 females, aged 6–72 (median, 35.5 years). Twenty-four tumors were primary tumors, and two were metastases. Upon manual review, NGS uncovered a *CIC::DUX4* fusion in 24 cases, a *CIC::LEUTX* fusion in one case, and no definitive *CIC* rearrangement in one case. NGS identified genomic gains in the transcription factor *ETV4* in 24/26 cases, including amplification (*N* = 5), copy number gain (*N* = 18), and subclonal gain (*N* = 1) (Fig. 1a). In addition, NGS analysis identified genomic gain of *MYC* in 14/26 cases, including *MYC* amplification (seven copies) with concurrent gain of chromosome 8 (*N* = 1), copy number gain of *MYC* only (*N* = 1), copy number gain of *MYC* and chromosome 8 (*N* = 10). Trisomy 8 was detected by FISH in two additional cases (*N* = 2) (Fig. 1b). These findings are in line with the previously reported additional gain of chromosome 8 and *MYC* amplification in *CIC::DUX4*-rearranged sarcoma^12^. Together, these data support a rationale for evaluating MYC as an oncogenic target in *CIC*-rearranged sarcoma.

### Creation and validation of novel patient-derived in vitro and in vivo models of *CIC*-rearranged sarcoma

**Fig. 2:**
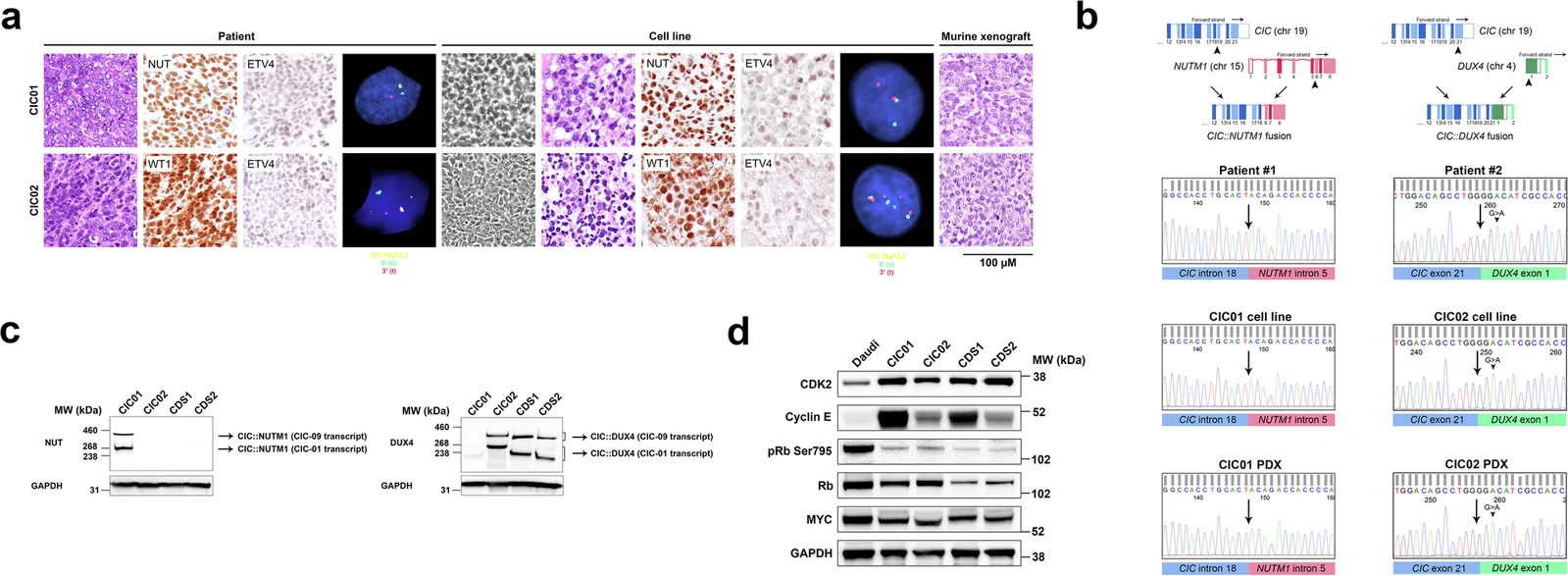
Creation and validation of novel patient-derived in vitro and in vivo models of *CIC*-rearranged sarcoma. **a** Overlapping morphologic features and expression of NUT, ETV4, and WT1 by immunohistochemistry in the CIC01 and CIC02 patient tumors, cell line, and PDX. *CIC* break-apart fluorescence in-situ hybridization demonstrates the presence of *CIC* gene rearrangement in CIC01 and CIC02. A separation of green (5′ centromeric) and red (3′ telomeric) probes indicates *CIC* rearrangement. Yellow indicates a normal signal (i.e., absence of *CIC* gene rearrangement). **b** Sanger sequencing of the breakpoint region identifies a *CIC::NUTM1* and *CIC::DUX4* fusion in the CIC01 and CIC02 cell lines, PDX, and primary tumors. **c** Mass spectrometry studies in CIC01 and CIC02 demonstrate the presence of CIC::NUT and CIC::DUX4 fusion oncoproteins, respectively, as visualized by immunoblotting studies. Fusion proteins appear as two bands resulting from a longer CIC-09 and shorter CIC-01 transcript. **d** Immunoblotting studies demonstrate that CIC01, CIC02, CDS1, and CDS2 expressed CDK2 and overexpressed cyclin E, while pRb Ser795 and Rb were retained. All four cell lines show strong MYC expression, comparable to levels detected in Daudi cells. GAPDH serves as loading control.

To enable the discovery of biological vulnerabilities for therapeutic targeting, we established two new cell lines and murine xenografts, CIC01 and CIC02, from snap-frozen tumor fragments of patients with *CIC*-rearranged sarcoma who underwent surgery in our program and provided informed consent. CIC01 was derived from a lung metastasis in a 25-year-old female who had a perineal primary tumor resected 10 months prior and had been treated with adjuvant chemotherapy consisting of vincristine, Adriamycin/doxorubicin, cyclophosphamide, ifosfamide, and etoposide (VDC/IE) as well as radiation to the perineum and pelvis. The tumor had also metastasized to the brain. CIC02 was derived from a locally recurrent tumor involving spleen, peritoneum, psoas muscle, and colon in a 39-year-old male who had a primary kidney tumor resected 6 months prior and had been treated with adjuvant chemotherapy (VDC/IE). To validate whether these new preclinical models closely represented the respective patient tumors, we performed microscopic evaluation, which highlighted that CIC01 and CIC02 primary tumors, cell lines, and PDX exhibited overlapping histomorphological and immunohistochemical features (Fig. 2a). Specifically, both primary tumors and cell lines demonstrated undifferentiated round cell morphology and weak nuclear expression of ETV4, a diagnostic marker of *CIC*-rearranged sarcomas. CIC01 demonstrated strong and diffuse nuclear expression of NUT which is observed in *NUTM1*-rearranged neoplasms, whereas CIC02 displayed strong and diffuse nuclear expression of WT1 which is expressed in most *CIC::DUX4* sarcomas (Fig. 2a). The CIC01 and CIC02 PDXs exhibited characteristic round cell features (Fig. 2a). Fluorescence in-situ hybridization (FISH) using a *CIC* break-apart probe revealed the presence of *CIC* rearrangement in CIC01 and CIC02 (Fig. 2a). Targeted next-generation sequencing (NGS) detected a rearrangement between *CIC* (NM_001386298.1) intron 18 (hg19 breakpoint: chr19:42,294,150) and *NUTM1* (NM_001284292.2) intron 5 (hg19 breakpoint: chr15:34,354,009) in CIC01 (Supplementary Fig. S1) and a rearrangement between *CIC* (NM_001386298.1) exon 21 (hg19 breakpoint: chr19:42,295,124) and *DUX4* (NM_001306068.3) exon 1 (hg19 breakpoint: chr4:190,173,906) in CIC02 (Supplementary Fig. S1). Sanger sequencing confirmed *CIC::NUTM1* and *CIC::DUX4* fusions in the CIC01 and CIC02 cell lines, PDX, and primary tumors (Fig. 2b). As controls, we utilized the NCC-CDS1-X1-C1 (subsequently abbreviated as “CDS1”)^13^ and NCC-CDS2-C1 (subsequently abbreviated as “CDS2”)^14^ cell lines provided by Tadashi Kondo (National Cancer Center Research Institute, Tokyo, Japan) with previously reported *CIC::DUX4* fusions (Supplementary Fig. S1). Mass spectrometry studies in CIC01 and CIC02 confirmed the presence of CIC::NUT and CIC::DUX4 fusion oncoproteins, respectively, which were visualized by NUT and DUX4 immunoblotting of CIC01, CIC02, CDS1, and CDS2 lysates. Fusion proteins appeared as two distinct bands resulting from a longer CIC-09 and shorter CIC-01 transcript (Fig. 2c and Supplementary Fig. S2). Together, these results indicate that the CIC01 and CIC02 cell lines and PDXs resemble the primary tumors from which they were established and represent additional preclinical models for subsequent in vitro and in vivo analyses, including the first-ever model of *CIC::NUTM1*-rearranged sarcoma.

Based on previous reports indicating molecular dependence on the CDK2-cyclin E pathway^15^ along with high cyclin E expression^31,32^ and frequent genomic gain of *MYC*, a regulator of CDK2-cyclin E^33^, we first assessed the CDK2-cyclin E pathway as a potential therapeutic vulnerability in our *CIC*-rearranged sarcoma models. Exploratory immunoblotting studies demonstrated that CIC01, CIC02, CDS1, and CDS2 expressed CDK2 and cyclin E, in line with previous reports indicating molecular dependence on the CDK2-cyclin E pathway^15^ (Fig. 2d and Supplementary Fig. S3). Furthermore, pRb Ser795 and Rb were retained, which was predictive of potential sensitivity to CDK2-targeted therapies (Fig. 2d and Supplementary Fig. S3)^34^. Notably, all four cell lines showed strong MYC expression, comparable to levels observed in Daudi (Burkitt lymphoma) cells, a prototypical MYC-driven cancer (Fig. 2d and Supplementary Fig. S3), further suggesting MYC as an oncogenic driver in *CIC*-rearranged sarcoma.

### Selective CDK2 inhibition and CRISPR-mediated *CDK2* knockout demonstrate anti-tumor effects

**Fig. 3:**
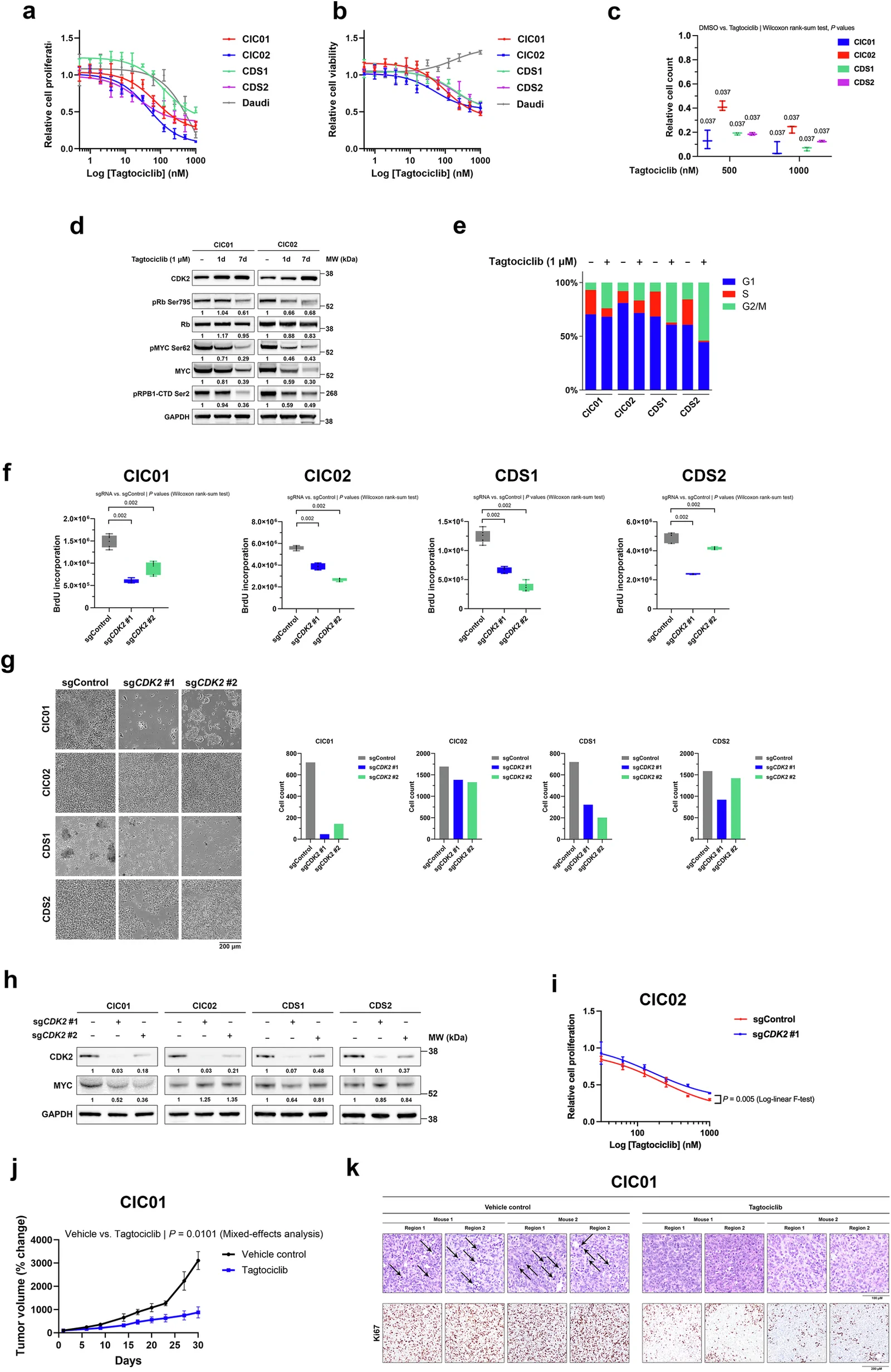
Selective CDK2 inhibition and CRISPR-mediated *CDK2* knockout demonstrate anti-tumor effects. Tagtociclib inhibits cell proliferation as assessed by BrdU incorporation at 24 h (**a**) and cell viability as assessed by CellTiter-Glo assay (**b**) in the CIC01, CIC02, CDS1, and CDS2 cell lines (normalized to DMSO). Tests were performed in triplicate (*N* = 3 biologically independent samples; mean +/− s.d) (**c**). Tagtociclib also reduces cell counts after 7 days at concentrations of 500 nM and 1 µM in the CIC01, CIC02, CDS1, and CDS2 cell lines (normalized to DMSO) (*P* = 0.037 each; two-sided Wilcoxon rank-sum test). Tests were performed in triplicate (*N* = 3 biologically independent samples; mean +/− s.d). **d** Immunoblotting analyses show that tagtociclib treatment inhibits the expression of phospho-Rb Ser795, phospho-MYC Ser62, total MYC, and phospho-RPB1-carboxyl-terminal domain (CTD) Ser2 in the CIC01 and CIC02 cell lines. This effect is more pronounced after 7 days of treatment than after 1 day of treatment. CDK2 expression remains unchanged. The calibrated expression is shown at the bottom of each row. GAPDH was used as the loading control. **e** BrdU incorporation (2 h) and flow cytometry analysis demonstrates that tagtociclib (1 µM, 7 d) increases the fraction of cells in the G2/M phase in the CIC01, CIC02, CDS1, and CDS2 cell lines and reduces the fraction of cells in the S phase in CIC01, CDS1, and CDS2 (*N* = 1 biological sample). **f** CRISPR/Cas9-mediated *CDK2* knockout using two different sgRNAs targeting *CDK2* results in a significant reduction in cell proliferation in the CIC01, CIC02, CDS1, and CDS2 cell lines for both sgRNAs as assessed by BrdU incorporation at 24 h and 8 days post-infection (*P* = 0.002 each; two-sided Wilcoxon rank-sum test). Tests were performed at least in triplicate (*N* = 3 biologically independent samples; mean +/− s.d). The empty vector served as a control. **g** Both sgRNAs directed against *CDK2* also reduced cell counts, as shown in phase-contrast photographs 14 days post-infection. The empty vector served as a control. Bar graphs illustrate corresponding cell counts obtained with semiautomated quantification using the Cellpose-SAM tool (*N* = 1 biological sample). **h** *CDK2* knockout not only reduced CDK2 expression but also MYC expression 7 days post-infection. **i** *CDK2* knockout significantly reduced the sensitivity to tagtociclib as assessed by BrdU incorporation at 24 h in the CIC02 cell line 12 days post-infection (normalized to DMSO) (*P* = 0.005; two-sided log-linear *F*-test). The test was performed in triplicate (*N* = 3 biologically independent samples; mean +/− s.d). **j** Tagtociclib treatment at 150 mg/kg BID for 28 days (*N* = 5 biologically independent animals) significantly reduced tumor volumes in the CIC01 PDX compared to the vehicle control (*P* = 0.0101; mixed-effects analysis accounting for repeated measurements over time) (*N* = 3 biologically independent animals; mean +/− s.e.m.). **k** Tagtociclib treatment at 150 mg/kg BID for 28 days also eliminated mitotic activity and Ki67 proliferation index in CIC01 PDX cells compared to the vehicle control (shown are *N* = 2 per arm with two regions sampled). Mitoses are indicated by arrows.

Dinaciclib, a pan-CDK inhibitor of CDK1, CDK2, CDK5, CDK9, and CDK12^35,36^, inhibited tumor growth and cell viability in *CIC*-rearranged sarcoma models^15^, leading us to next assess the efficacy of the selective CDK2 inhibitors tagtociclib (PF-07104091) and BLU-222 in our *CIC*-rearranged sarcoma cell lines and PDXs. Although highly selective for CDK2, tagtociclib also inhibits CDK1 and CDK9 to some extent^37^. Treatment of the *CIC*-rearranged cell lines with tagtociclib substantially inhibited cell proliferation (Fig. 3a) with IC_50_ values ranging from 23.91–125.4 nM (Supplementary Table S1), whereas cell viability was reduced to a lesser extent (Fig. 3b). In the MYC-driven Daudi control cell line, cell proliferation, but not viability, was inhibited (Fig. 3a, b). There was no effect on apoptosis, as evidenced by the lack of induction of caspase 3/7 activity (Supplementary Fig. S4a). Comparable results were obtained with BLU-222 (Supplementary Fig. S4b–d, Supplementary Table S1, and Supplementary Fig. S5). Tagtociclib treatment also significantly reduced cell counts in the CIC01, CIC02, CDS1, and CDS2 cell lines (Fig. 3c). Immunoblotting analyses showed that tagtociclib inhibited the expression of the CDK2 substrates phospho-Rb Ser795 and phospho-MYC Ser62^38^ in the CIC01 and CIC02 cell lines (Fig. 3d and Supplementary Fig. S6). This effect was more pronounced after 7 days of treatment than after 1 day of treatment (Fig. 3d and Supplementary Fig. S6). To determine the effects of tagtociclib on cell cycle progression, BrdU incorporation and flow cytometry revealed that tagtociclib increased the fraction of cells in G2/M phase in all *CIC*-rearranged sarcoma cell lines and reduced the fraction of cells in S phase in CIC01, CDS1, and CDS2 (Fig. 3e). Together, these findings demonstrate that tagtociclib induces cell cycle arrest and inhibits CDK2-mediated effects. Surprisingly, tagtociclib also reduced the expression of the CDK9-specific phospho-RPB1-carboxyl-terminal domain (CTD) Ser2 (Fig. 3d and Supplementary Fig. S6). The reduction in total MYC (Fig. 3d and Supplementary Fig. S6) could be an effect of reduced phosphorylation at Ser62-mediated stability or reduced phospho-CTD Ser2-mediated transcription. Together, these studies are consistent with inhibition of CDK2 with some activity against CDK1 and CDK9.

To assess whether *CDK2* knockout could recapitulate the anti-proliferative effects observed with tagtociclib, we used a CRISPR/Cas9 all-in one vector system and two different sgRNAs targeting *CDK2*. *CDK2* knockout with either sgRNA significantly reduced proliferation of CIC01, CIC02, CDS1, and CDS2 cell lines as assessed by BrdU incorporation 8 days post-infection (Fig. 3f) and cell culture density 14 days post-infection (Fig. 3g). Interestingly, *CDK2* knockout not only reduced CDK2 expression but also MYC expression in three cell lines 7 days post-infection (Fig. 3h and Supplementary Fig. S7), possibly due to reduced CDK2-mediated phosphorylation of MYC at Ser62, which affects MYC stability and senescence^39,40^. *CDK2* knockout significantly reduced the sensitivity of the CIC02 cell line to tagtociclib as assessed by BrdU incorporation 12 days post-infection when using an *F*-test applied to the corresponding log-linear models, thereby confirming the relative target selectivity of tagtociclib for CDK2 (Fig. 3i).

To validate these observations in vivo, we treated the CIC01 PDX with tagtociclib (150 mg/kg, twice a day [BID]) for 28 days and showed that CDK2 inhibition significantly reduced tumor volumes (Fig. 3j), mitotic activity, and Ki67 proliferation index (Fig. 3k). We also assessed the efficacy of the combined CDK2/4/6 inhibitor PF-06873600 and the CDK4/6 inhibitor palbociclib in *CIC*-rearranged sarcoma cell lines. Cell proliferation (Supplementary Fig. S8a, b and Supplementary Table S1), cell counts (Supplementary Fig. S8c), and cell viability (Supplementary Fig. S8d, e) were reduced in all cell lines treated with PF-06873600, but not with palbociclib, suggesting that the effect of PF-06873600 was due to its inhibitory effect on CDK2. In summary, the above studies highlight anti-tumor effects of CDK2 inhibition in *CIC*-rearranged sarcoma in vitro and in vivo.

### Tagtociclib inhibits MYC oncogenic programs and reduces MYC protein stability

**Fig. 4:**
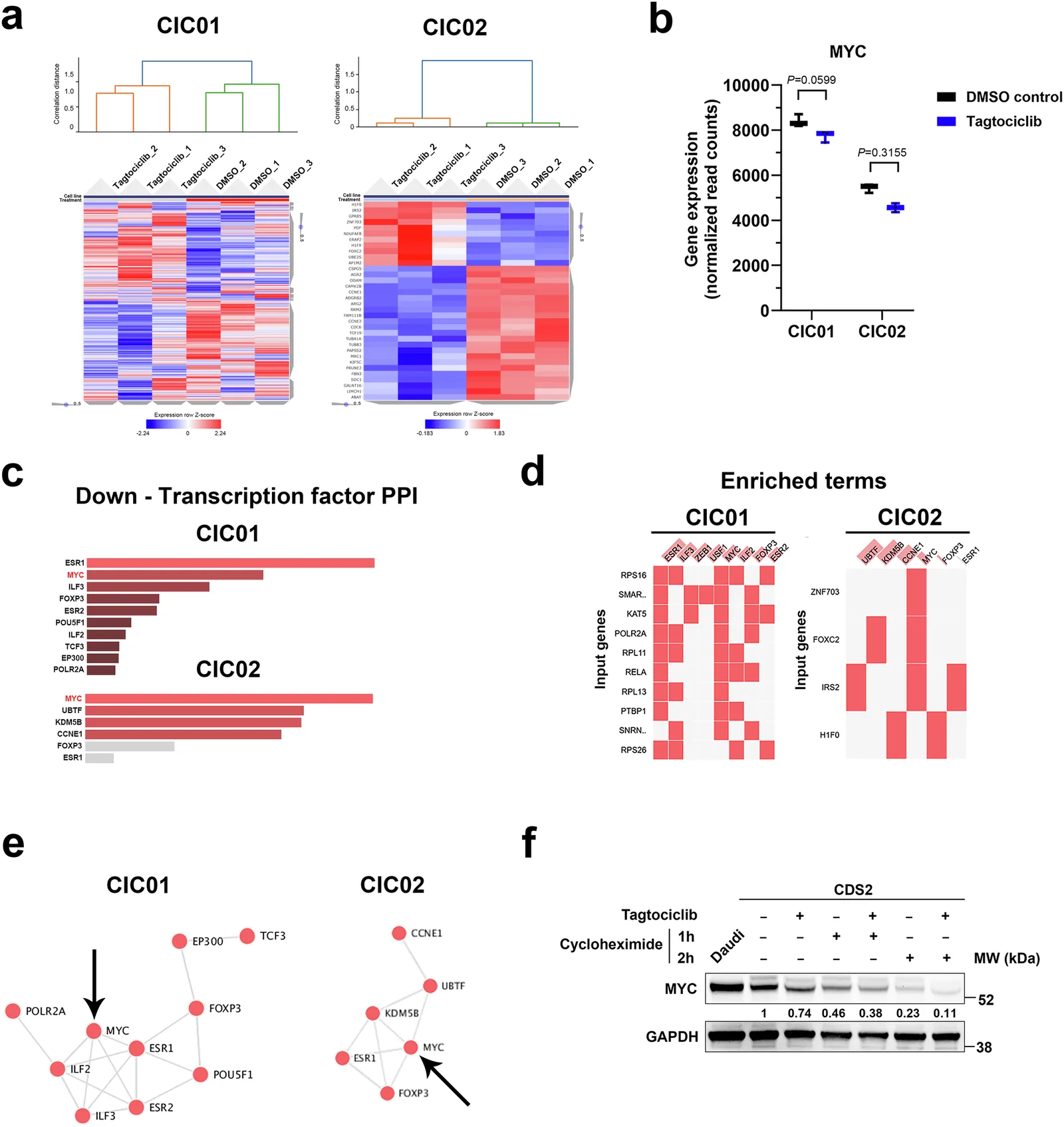
Tagtociclib inhibits MYC oncogenic programs and reduces MYC protein stability. **a** RNA sequencing analysis was performed in triplicate in the CIC01 and CIC02 cell lines after treatment with tagtociclib at 200 nM for 48 h. Unsupervised hierarchical clustering demonstrates that tagtociclib treated cells separated from DMSO-control within each cell line. **b** Tagtociclib treatment did not suppress MYC transcript expression in CIC01 and CIC02 (two-sided Wald test with Benjamini–Hochberg adjustment for multiple comparisons; adjusted *P* = 0.0599 and *P* = 0.3155, respectively). The test was performed in triplicate (*N* = 3 biologically independent samples). **c** Analysis of the transcription factor protein-protein interactions (PPI) library demonstrates that tagtociclib treatment results in downregulation of the MYC transcription factor signature in CIC01 and CIC02, indicating inhibition of genes encoding proteins that interact with MYC. Signatures are ranked by *P* value. Red bars indicate *P* ≤ 0.05; gray bars indicate *P* > 0.05. **d** Heatmap showing the genes downregulated by tagtociclib treatment in CIC01 and CIC02 that contribute to the MYC transcription factor signature. **e** Functional networks for the MYC transcription factor signature highlighting MYC and other genes significantly downregulated by tagtociclib treatment in CIC01 and CIC02. **f** Combined treatment with tagtociclib at 1 µM for 6 days and cycloheximide at 178 µM for 1 or 2 h produced an additive reduction in MYC protein expression, supporting a model in which tagtociclib primarily reduces MYC stability rather than inhibiting MYC translation.

To determine the effects of CDK2 inhibition at the transcriptomic level, we performed RNA sequencing analysis of the CIC01 and CIC02 cell lines in response to tagtociclib. Unsupervised hierarchical clustering demonstrated that cells treated with tagtociclib were separated from DMSO-control within each cell line (Fig. 4a). All significantly differentially expressed genes identified in CIC02 and CIC01 were included in the enrichment analyses. *CCNE1* and *CCNE2* were among the 11 most significantly upregulated genes common in CIC01 and CIC02 cells in response to tagtociclib treatment (Supplementary Fig. S9), indicating compensatory cyclin E upregulation upon CDK2 inhibition. Tagtociclib treatment did not suppress MYC transcription (Fig. 4b), suggesting that the observed reduction in MYC expression and downregulation is not primarily mediated at the MYC transcriptional level. However, analysis of the transcription factor protein-protein interactions library uncovered that tagtociclib treatment of CIC01 and CIC02 cells resulted in downregulation of genes encoding proteins that interact with MYC, indicating an indirect effect of tagtociclib treatment on MYC signatures (Fig. 4c–e and Supplementary Table S2). We next examined the effects of tagtociclib on MYC protein stability using cycloheximide, a strong inhibitor of protein translation, to assess protein turnover. Combined treatment with tagtociclib and cycloheximide produced an additive reduction in MYC protein expression (Fig. 4f), supporting a model in which tagtociclib primarily decreases MYC stability rather than inhibiting MYC transcription. Together, these findings suggest that CDK2 inhibition in *CIC*-rearranged sarcoma cells downregulates MYC transcriptional activity by reducing MYC protein stability.

### *CIC*-rearranged sarcomas are driven by MYC and respond to CDK9 inhibition

**Fig. 5:**
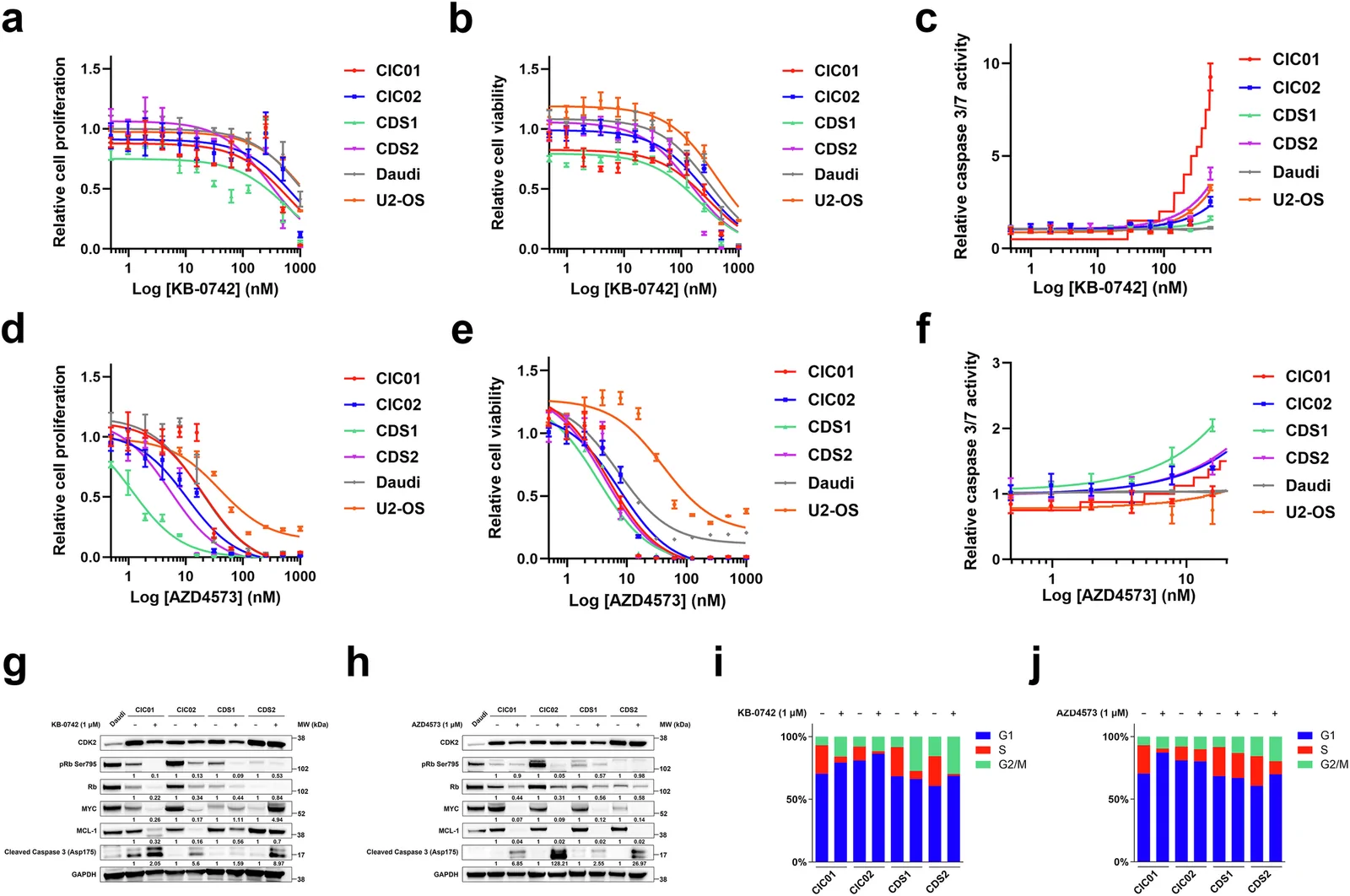
*CIC*-rearranged sarcomas are driven by MYC and respond to CDK9 inhibition. KB-0742 inhibits cell proliferation as assessed by BrdU incorporation at 24 h (**a**) and cell viability as assessed by CellTiter-Glo assay (**b**) in CIC01, CIC02, CDS1, and CDS2. KB-0742 also induces apoptosis in CIC01, CIC02, CDS1, and CDS2 as measured by Caspase-Glo 3/7 assay. Tests were performed in triplicate (*N* = 3 biologically independent samples; mean +/− s.d) (**c**). AZD4573 inhibits cell proliferation as assessed by BrdU incorporation at 24 h (**d**) and cell viability as assessed by CellTiter-Glo assay (**e**) in CIC01, CIC02, CDS1, and CDS2. AZD4573 also induces apoptosis in CIC01, CIC02, CDS1, and CDS2 as measured by Caspase-Glo 3/7 assay. Tests were performed in triplicate (*N* = 3 biologically independent samples; mean +/− s.d) (**f**). MYC-independent U2-OS cells were substantially less sensitive to either treatment (**a–f**). Immunoblotting studies reveal that treatment with KB-0742 (48 h) (**g**) and AZD4573 (12 h) (**h**) inhibits phospho-Rb and total Rb expression, as well as MYC and MCL-1 expression, and increases levels of cleaved caspase 3 (Asp175), specifically the 17-kDa form. The calibrated expression is shown at the bottom of each row. The 17-kDa band was used for calibration of cleaved caspase 3 (Asp175). GAPDH was used as the loading control. BrdU incorporation (2 h) and flow cytometry analysis demonstrate that KB-0742 (1 µM, 48 h) increases the fraction of cells in the G2/M phase and blocks S phase entry in CIC01, CIC02, CDS1, and CDS2 (*N* = 1 biological sample) (**i**), whereas AZD4573 (1 µM, 12 h) increases the fraction of cells in G2/M in all cell lines and blocks S phase entry in CIC01 and CDS2 (*N* = 1 biological sample) (**j**).

Given the high levels of MYC expression in our four cell line models, we aimed to determine whether direct inhibition of MYC affects tumor growth in *CIC*-rearranged sarcoma. We therefore tested the MYC-MAX dimerization inhibitors 10074-G5 and 10058-F4 in our cell lines and found that both compounds inhibited cell viability in CIC01, CIC02, CDS1, and CDS2 cells, comparable to effects seen in Daudi cells, but not in the MYC-independent osteosarcoma cell line U2-OS (Supplementary Fig. S10). We next assessed the efficacy of the CDK9 inhibitors KB-0742^41^ and AZD4573^42^, which are currently in clinical development and demonstrate preclinical efficacy in MYC-dependent cancers. Our cell-based assays demonstrated that KB-0742 inhibited cell proliferation and cell viability and increased apoptosis in CIC01, CIC02, CDS1, and CDS2 (Fig. 5a–c), with proliferation IC_50_ values ranging from 304.1 to 744.4 nM (Supplementary Table S1). Similarly, treatment with AZD4573 inhibited cell proliferation and cell viability and increased apoptosis in CIC01, CIC02, CDS1, and CDS2 (Fig. 5d–f), with proliferation IC_50_ values ranging from 1.16 to 20.49 nM (Supplementary Table S1). U2-OS cells were substantially less sensitive to either treatment (Fig. 5a–f). Immunoblotting studies revealed that KB-0742 and AZD4573 inhibited phospho-Rb and total Rb expression, as well as MYC and MCL-1 expression and led to an increase in the expression of cleaved caspase 3 (Asp175), specifically the 17-kDa form of activated caspase 3 (Fig. 5g, h and Supplementary Figs. S11 and 12). A compensatory increase of MYC expression induced by KB-0742 was observed in CDS2 and to a lesser extent in CDS1 (Fig. 5g and Supplementary Fig. S11), a phenomenon reported to occur in some cancer cells after CDK9 inhibition^43^. This observation was further validated in a time course experiment comparing MYC expression in CDS2 after treatment with KB-0742 for 6 to 72 h which showed a temporary increase in MYC and MCL-1 expression peaking at 36 h of treatment, along with an increase in cleaved caspase 3 expression peaking at 72 h of treatment (specifically the 17-kDa form), indicating effective inhibition of MYC downstream pathways (Supplementary Figs. S13 and S14). BrdU incorporation and flow cytometry analysis demonstrated that KB-0742 increased the fraction of cells in G2/M phase and blocked S phase entry in CIC01, CIC02, CDS1, and CDS2 (Fig. 5i), whereas AZD4573 increased the fraction of cells in the G2/M in all cell lines and blocked S phase entry in CIC01 and CDS2 (Fig. 5j). Overall, these findings highlight anti-tumor effects of CDK9 inhibition in *CIC*-rearranged sarcoma through the inhibition of MYC oncogenic signaling and induction of apoptotic cell death.

### Combined CDK2 and CDK9 inhibition is synergistic and inhibits cell proliferation and viability

**Fig. 6:**
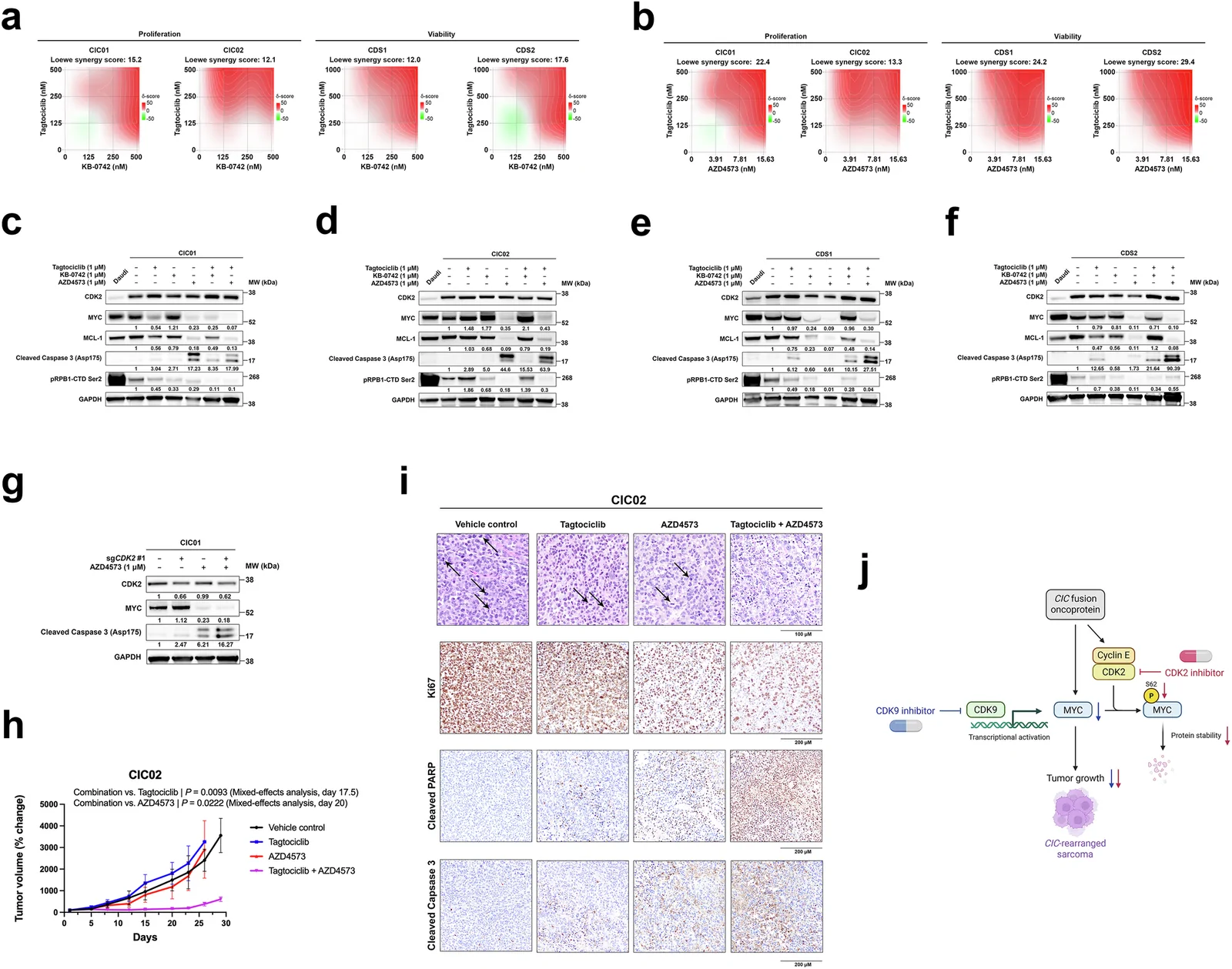
Combined CDK2 and CDK9 inhibition is synergistic and inhibits cell proliferation and viability. **a** Synergy distributions for the interaction of tagtociclib and KB-0742 demonstrate drug synergy (Loewe score >10) in CIC01 and CIC02 for cell proliferation as assessed by BrdU incorporation at 24 h and for cell viability in CDS1 and CDS2 as assessed by CellTiter-Glo assay. Tests were performed in triplicate. **b** Synergy distributions for the interaction of tagtociclib and AZD4573 also demonstrate drug synergy (Loewe score >10) in CIC01 and CIC02 for cell proliferation as assessed by BrdU incorporation at 24 h and for cell viability in CDS1 and CDS2 as assessed by CellTiter-Glo assay. Tests were performed in triplicate. Corresponding immunoblotting studies show that combined treatment of CIC01 (**c**), CIC02 (**d**), CDS1 (**e**), and CDS2 (**f**) with tagtociclib (7 d) and KB-0742 (6 h) or with tagtociclib (7 d) and AZD4573 (6 h) has a greater effect on inhibition of MYC and MCL-1 expression, increase of cleaved caspase 3 expression (17-kDa form), and inhibition of phospho-RPB1-CTD Ser2 than single-agent treatment with tagtociclib (7 d), KB-0742 (6 h), or AZD4573 (6 h). Expression of CDK2 is unchanged. Calibrated expression shown at the bottom of each row. GAPDH serves as loading control. **g** Combination of *CDK2* knockout and AZD4573 (6 h) also had a greater effect on inhibition of MYC and increase of cleaved caspase 3 expression in CIC01 compared to *CDK2* knockout or AZD4573 alone. Calibrated expression shown at the bottom of each row. GAPDH serves as loading control. **h** Combined treatment with tagtociclib at 120 mg/kg BID PO and AZD4573 at 10 mg/kg Q2H BID 2ON 5OFF IP for 28 days (*N* = 5 biologically independent animals) significantly reduced tumor volumes in the CIC02 PDX compared to single-agent tagtociclib at 120 mg/kg BID PO (*N* = 5 biologically independent animals) (*P* = 0.0093; mixed-effects analysis accounting for repeated measurements over time, adjusted for multiple comparisons using the Benjamini–Hochberg method) or single-agent AZD4573 at 10 mg/kg Q2H BID 2ON 5OFF IP (*N* = 4 biologically independent animals) (*P* = 0.0222; mixed-effects analysis accounting for repeated measurements over time, adjusted for multiple comparisons using the Benjamini–Hochberg method; mean +/− s.e.m.) (vehicle control *N* = 3 biologically independent animals). **i** Combined treatment with tagtociclib at 120 mg/kg BID PO and AZD4573 at 10 mg/kg Q2H BID 2ON 5OFF IP for 10 days eliminated mitotic activity, induced apoptotic cell death, substantially reduced the Ki67 proliferation index, and induced expression of cleaved PARP (Asp 214) and cleaved caspase 3 (Asp175) compared to single-agent tagtociclib at 120 mg/kg/d or single-agent AZD4573 at 10 mg/kg/d in CIC02 (*N* = 1 each). Mitoses are indicated by arrows. **j** Schematic illustrating the key roles and interactions of CDK2 and CDK9 in relation to MYC signaling and *CIC* fusion-driven sarcoma biology.

Based on the observation that CDK2 inhibition and CDK9 inhibition alone had anti-tumor effects in our preclinical models of *CIC*-rearranged sarcoma, we next determined whether concurrent inhibition of CDK2 and CDK9 would augment these effects. Cell-based assays demonstrated that combined treatment of the CIC01, CIC02, CDS1, and CDS2 cell lines with tagtociclib and KB-0742 (Fig. 6a) or with tagtociclib and AZD4573 (Fig. 6b) had a synergistic effect on the inhibition of cell proliferation and cell viability (Supplementary Fig. S15 and Supplementary Table S3). Corresponding immunoblotting studies demonstrated a greater effect on the inhibition of MYC and MCL-1 expression, increased cleaved caspase 3 expression, and inhibition of phospho-RPB1-CTD Ser2 after combination treatment in all four cell lines compared to single-agent treatment (Fig. 6c–f and Supplementary Figs. S16–S19). The combination of *CDK2* knockout and AZD4573 in the CIC01 cell line also had a greater effect on the inhibition of MYC and an increase in cleaved caspase 3 expression than *CDK2* knockout or AZD4573 alone (Fig. 6g and Supplementary Fig. S20). To validate these observations in vivo, we treated the CIC02 PDX with a tolerated combination of low-dose tagtociclib (120 mg/kg BID PO) and AZD4573 (10 mg/kg Q2H BID 2ON 5OFF IP) for 28 days and showed that combined CDK2 and CDK9 inhibition significantly reduced tumor volumes compared to single-agent tagtociclib or single-agent AZD4573 (Fig. 6h). Combined treatment with tagtociclib and AZD4573 for 10 days eliminated mitotic activity, induced apoptotic cell death, substantially reduced the Ki67 proliferation index, and induced expression of cleaved PARP (Asp 214) and cleaved caspase 3 (Asp175) compared to single-agent tagtociclib or single-agent AZD4573 in CIC02 (Fig. 6i). Together, these results demonstrate that concurrent CDK2 and CDK9 inhibition has greater anti-tumor effects in preclinical models of *CIC*-rearranged sarcoma than CDK2 or CDK9 inhibition alone through combined proliferative arrest and induction of apoptosis via CDK9-mediated inhibition of MYC transcription and CDK2-mediated impairment of MYC stability (Fig. 6j).

## Discussion

*CIC*-rearranged sarcoma is a molecularly defined aggressive sarcoma subtype that disproportionally occurs in adolescents and young adults, for which no effective therapies currently exist^1,2^. Its poor response to conventional chemotherapy^1,2,11^ highlights the critical need to uncover oncogenic drivers and other resistance mechanisms for therapeutic targeting.

By leveraging *CIC*-rearranged human sarcoma cell lines and PDX models, we discovered that these cancers are MYC-dependent and can be targeted effectively with CDK2 and CDK9 inhibitors. While CDK2 depletion is not expected to have anti-tumor effects in all cell types because other CDKs, such as CDK1, can compensate for CDK2 loss^44^, our data revealed that all *CIC*-rearranged sarcoma cell lines were sensitive to *CDK2* knockout, supporting the selective oncogenic dependence of this sarcoma subtype on CDK2. We showed that therapeutic CDK2 inhibition and *CDK2* knockout inhibited canonical CDK2 targets, such as Rb phosphorylation at Ser795 and induced cell cycle arrest. The observed difference in therapeutic CDK2 inhibition versus *CDK2* knockout may reflect dosing and specificity of tagtociclib, as well as potential off-target activity of CDK2 inhibitors on CDK1 and CDK9 or a combination of these. Additionally, incomplete knockout efficiency, as evidenced by residual CDK2 expression, may contribute to the phenotype we observed. CDK2 has been identified as a critical mediator of acquired CDK4/6 inhibitor resistance in breast cancer^45–47^. The selective CDK2 inhibitor tagtociclib has anti-tumor activity in CDK4/6 inhibitor-resistant breast cancer^48^ where it induces G1 arrest. Tagtociclib is being evaluated for advanced or metastatic small cell lung, breast, and ovarian cancers, as well as other solid tumors (NCT04553133, NCT05262400). Interestingly, our studies also demonstrated that CDK2 inhibition and *CDK2* knockout resulted in downregulation of MYC expression through decreased MYC protein stability rather than inhibition of MYC transcription (Fig. 6j). Of note, previous studies have suggested that phosphorylation of MYC at Ser62 is involved in the recruitment and maintenance of MYC in subsets of MYC-regulated genes involved in managing oncogene-induced and other types of stresses that can cause apoptosis or senescence^38,49^. Pharmacological inhibition of CDK2-mediated Ser62 phosphorylation has been shown to result in the depletion of MYC from genes involved in senescence regulation despite continuous MYC expression, resulting in the abrogation of MYC-mediated senescence suppression. CDK2 has also been shown to directly regulate MYC-mediated repression of RAS-induced senescence through phosphorylation of MYC at Ser62^50^. Depletion or pharmacological inhibition of CDK2 could restore RAS-induced senescence as a potentially useful strategy for targeting MYC in tumors^51^.

CDK9, a Ser/Thr protein kinase and catalytic subunit of positive transcription elongation factor b, regulates transcriptional elongation by phosphorylating RNA polymerase II at serine 2 (pRPB1-CTD Ser2). Transient inhibition of CDK9 modulates the expression of genes with short-lived transcripts and labile proteins, providing a potential therapeutic opportunity in cancers dependent upon oncogenes fitting these criteria^52^. KB-0742^41,53,54^ and AZD4573^42,52^ are potent and selective ATP-competitive CDK9 inhibitors that inhibit phospho-RPB1-CTD Ser2, thereby decreasing the expression of MCL-1, a member of the BCL-2 family of proteins that prevents apoptosis and the oncogene MYC, which induces apoptosis in a broad range of malignancies. KB-0742 and AZD4573 are being evaluated clinically for MYC-dependent hematologic malignancies (NCT04718675, NCT05140382, NCT04630756, and NCT03263637).

In our preclinical models of *CIC*-rearranged sarcoma, we found that tagtociclib not only resulted in Rb-mediated proliferative arrest but also suppressed MCL-1 and MYC expression, similar to KB-0742 and AZD4573, along with an increase in cleaved caspase 3 expression. Of note, two recent studies identified sensitivity of *CIC*-rearranged sarcoma tumoroids to MCL-1 inhibitors which, however, have limited clinical utility due to cardiac side effects^55,56^. Our studies further highlighted that the combination of tagtociclib with KB-0742 or AZD4573 was synergistic and induced proliferative arrest and induction of caspase 3-mediated apoptotic cell death in all four cell lines. We also showed that AZD4573 had a greater effect when combined with *CDK2* knockout. While the concurrent inhibition of CDK2 and CDK9 has not been tested in cancer patients, tagtociclib, KB-0742, and AZD4573 are generally well tolerated with manageable toxicity^48,52,54^. Our studies showed that the toxicity of tagtociclib and AZD4573 in combination was tolerable in mice. Notably, our results likely explain the previously reported activity of dinaciclib in *CIC*-rearranged sarcoma models, although potent concurrent CDK1 inhibition by dinaciclib likely contributed to a steep dose-toxicity relationship that limited clinical development in patients with solid tumors^57,58^.

The models we used included three cell lines defined by a *CIC::DUX4*^5^ fusion and one with a *CIC::NUTM1*^6,7^ fusion, thereby making the results of our work applicable to the most common molecular subtypes of *CIC*-rearranged sarcoma. The *CIC::DUX4* fusion converts *CIC* from a transcriptional repressor to a transcriptional activator and drives specific tumorigenic programs^59,60^. Okimoto et al. reported that the CIC::DUX4 fusion oncoprotein leads to increased expression of ETS variant 4 (ETV4) and cyclin E1 (CCNE1) via direct neomorphic effects and drives metastasis and tumor cell survival, respectively^15^. ETV4 is overexpressed in the majority of *CIC*-rearranged sarcomas, resulting from ETV4 transcript upregulation^5,61^; a subset of cases also show low-level/subclonal *ETV4* gain^62^. While the interaction between *ETV4* and *MYC* has not been studied in *CIC*-rearranged sarcoma, *ETV4* has previously been shown to modulate MYC expression in myeloma^63^ and prostate cancer^64^. Our genomic profiling studies demonstrated near-ubiquitous oncogenic activation of MYC in clinical samples of *CIC*-rearranged sarcoma, either directly or indirectly, through the genomic gain of *ETV4*. Of note, response to CDK2 and CDK9 inhibition seems to be independent of *MYC* copy number, as only CDS2 shows *MYC* copy number gain, whereas CIC01, CIC02, and CDS1 do not. These findings support the concept of MYC dependence in *CIC*-rearranged sarcoma and provide further justification for MYC-targeted treatment strategies.

In summary, our study highlights *CIC*-rearranged sarcoma as a MYC-dependent cancer and demonstrates anti-tumor effects of concurrent inhibition of CDK2 and CDK9 through proliferative arrest and induction of caspase 3-mediated apoptotic cell death in preclinical models of *CIC*-rearranged sarcoma. We further identify distinct but complementary mechanisms of MYC regulation, whereby CDK9 inhibition suppresses MYC transcription and CDK2 inhibition impairs MYC protein stability. Our findings provide key preclinical data that could serve as a rationale for clinical trials evaluating CDK2 and CDK9-targeted therapies in patients with *CIC*-rearranged sarcoma.

## Supplementary information

Transparent Peer Review file

Supplementary Material File

Description of Additional Supplementary files

Supplementary Data 1

ARRIVE Checklist

## Data Availability

The data generated in this study are publicly available in GenBank (NCBI) under accession number PQ299793 (https://www.ncbi.nlm.nih.gov/nuccore/PQ299793) and PQ227342 (https://www.ncbi.nlm.nih.gov/nuccore/PQ227342), Gene Expression Omnibus (GEO) under accession number GSE291596 (https://www.ncbi.nlm.nih.gov/geo/query/acc.cgi?acc=GSE291596), and within the article and its supplementary data files. As per genomic data sharing guidelines, sequence and assay data were deposited in a publicly available database sponsored by the American Association for Cancer Research (AACR) Project GENIE (https://www.aacr.org/professionals/research/aacr-project-genie/aacr-project-genie-data/). Variant calls and a limited clinical dataset from patients are sent to the Synapse platform, developed by Sage Bionetworks, where the data are harmonized and protected health information (PHI) removed in a secure Health Insurance Portability and Accountability Act (HIPAA)-compliant environment that provides data governance. Raw numerical data underlying the graphs in Figs. 3–6 and Supplementary Figs. S4, S8, and S10 are provided in Supplementary Data 1. Source data underlying immunoblots are provided as uncropped and unprocessed images in Supplementary Figs. S2, S3, S5–S7, S11, S12, S14, and S16–S20.
