# Supplementary material for: Co-targeting CDK2 and CDK9 inhibits MYC oncogenic programs and has anti-tumor effects in CIC-rearranged sarcoma
Source: Commun Med (Lond). 2026 Oct 7;6:572. doi: 10.1038/s43856-026-01956-1 (PMC13728178; doi:10.1038/s43856-026-01956-1)
Supplement: Supplementary file 1 [file 43856_2026_1956_MOESM1_ESM.pdf]

nature portfolio

Peer Review File

Co-targeting CDK2 and CDK9 inhibits MYC oncogenic programs and has anti-tumor effects in *CIC*-rearranged sarcoma

Corresponding Author: Dr Inga-Marie Schaefer

This file contains all reviewer reports in order by version, followed by all author rebuttals in order by version.

Version 0:

Reviewer comments:

Reviewer #1

(Remarks to the Author)
Odom-Lewis et al., report on their preclinical studies that further define a targetable dependency in CIC-rearranged sarcoma. Specifically, they leverage prior studies that demonstrate a dependence on CDK2-cyclin E complex in CIC::DUX4 models but now expand these findings into other non-CIC::DUX4 tumors including CIC::NUTM1 for which they generate a new cell-based model system. The findings of this study are of importance due to the paucity of effective treatments for CIC-rearranged cancers but also due to the lack of clinical advancement of CDK2 inhibitors in the late-phase space. Thus, the aim of this study was to identify the key CDKs that promote survival and to leverage this insight to refine the therapeutic approach that limits drug toxicity of older less-specific CDK2 inhibitors such as dinaciclib.

Through their studies, they define CDK9 and CDK2 as key molecular and therapeutic targets that potentially converge on Myc in CIC-rearranged sarcomas. They argue that the therapeutic effect of targeting CDK2 can be augmented with combinatorial approaches using drugs that indirectly suppress MYC expression. Overall, this is a well-written and straightforward preclinical study that further develops a potential therapeutic strategy for CIC-rearranged sarcoma.

Major Comments:
1. Figure 5H. The tumor growth curve in Figure 5H (CIC02 xenografts) requires correlative data including an assessment of proliferative and apoptotic markers (Cl-caspase by IHC and/or Parp-cleavage by protein expression) in the explanted tumors to support their claim that the observed effect is through “proliferative arrest and induction of apoptosis”.

2. Since a major point in the manuscript is the dependence on MYC, it is important to at least attempt to define a mechanism by which MYC is regulated in CIC-rearranged cells. Thus, please attempt to rescue MYC expression using a MYC ser62 mutant isoform in at least one tagtociclib treated CIC-rearranged cell line to further define the mechanism of MYC regulation (post-tranlational vs transcriptional).

3. While it is appreciated that the MYC-MAX dimerization inhibitors decrease viability (Supplmental Figure 5) in all CIC-rearranged cell lines it is important to include a proper MYC-independent cell line for control.

Minor Comments:

1. A summary schematic/cartoon figure that highlights the key CDKs and how they interact with MYC and CIC fusions would help the reader understand this rather complex pathway.

2. Please expand the discussion to speculate why tagociclib treatment is less effective than CDK2 knockout? Is this the result of incomplete inhibition of CDK2 by tagociclib? Alternatively, are there off-target effects from tagociclib that enable partial survival ?

3. Please reference the following studies that demonstrate increased CCNE levels in CIC::DUX4 tumors and thus lends additional support to the authors findings: PMID: 38703529, PMID: 38705279

Reviewer #2

(Remarks to the Author)
In this paper, the authors characterize two new PDX/cell line models of CIC rearranged sarcomas and address therapeutic targeting. Although the reported findings do support the idea of targeting CDK2 and CDK9 in the treatment of this disease, several issues should be addressed.

Since the patients from whom the new cell lines were derived have metastatic disease, it would be great to know if the sample was obtained from the primary tumor or from a metastasis. If the latter, where was it? And were these samples collected before or after administration of chemotherapy?

The data in Figure 1 demonstrate that the anticipated defining molecular features are present, much more work (for example, RNA-seq comparisons, global epigenomic profiling, etc) would be needed to validate the statement that these cell lines and PDXs "closely resemble" the primary tumors.

Most cancer cells express Cyclin E and cdk2, and the mere presence of retained RB1 or high level protein expression of MYC are not evidence of molecular dependence or an oncogenic driver.
The experiments in Figure 2 lack appropriate negative controls. Almost any rapidly proliferating cell line would be expected to slow down when treated with an inhibitor of a key mediator of cell cycle progression, like CDK2. If the authors wish to prpose that this is somehow a unique characteristic of CIC rearranged sarcomas, then either showing lack of efficacy of an inhibitor of a different cyclin-dependent kinase or lack of effect of tagtociclib on a related sarcoma would be necessary. This criticism applies to both inhibitor and KO experiments.

Similarly, experiments in Figure 4 would be more convincing that CDK2 and CDK9 are somewhat unique vulnerabilities of this tumor type if appropriate negative controls (for example, another small round blue cell sarcoma) were included to demonstrate that this approach works for CIC rearranged sarcomas and not for others.

Reviewer #3

(Remarks to the Author)
In this manuscript, Odom-Lewis, et. al. establish new CIC-rearranged sarcoma models (cell lines and patient-derived xenografts [PDXs]) and use these models to explore the biology and therapeutic vulnerabilities in these tumors. They find that CIC-rearranged tumors frequently contain MYC amplification and a dependence on CDK2. Both pharmacological and genetic approaches to target CDK2 reduce MYC levels, impair cell cycle progression, proliferation, and change gene expression. Blocking CDK2 pharmacologically with tagtociclib also slows PDX growth in vivo. The authors further demonstrate reduced cell viability following inhibition of the MYC-MAX interaction and CDK9. Notably, CDK9 inhibitors induce apoptosis with cleaved caspase demonstrated. CDK2 and CDK9 inhibition are shown to synergistically reduce proliferation, cell viability, and in vivo xenograft growth. Together, this is an interesting manuscript, these are promising results, and this study is appropriate for publication in Communications Medicine, pending completion of revisions outlined below.

There are several strengths of this paper, including the curation of a small panel of patient proximal models that clearly capture some heterogeneity in fusions and response to treatment presented here, as well as analysis of clinical samples to investigate genomic gains of ETV4 and MYC. The authors do a good job of highlighting differences in how their cell lines respond to treatment where it is relevant. Another strength is the use of a diverse set of pharmacological compounds with varying degrees of target specificity to confirm results, coupled with genetic approaches for CDK2 depletion to validate the pharmacological findings. The xenograft data are compelling and the writing is generally clear and straightforward. The transcriptomic analysis and its presentation is a major weakness in this paper, and there are several additional weaknesses related to the presentation of the data described that need to be addressed before this manuscript can be published. See our comments below.

1) Line 61 (Introduction)-The authors included helpful details on the tumor type, age range, and fusion proteins. It would be great to also provide a brief, expanded explanation of CIC rearranged tumors, since they are rare. It may be useful to mention the typical body sites involved, common metastasis locations, any current treatment approaches and their success rates, and whether this cancer has been reported in children or in older adults over 30.

2) Line 65: (Introduction) "our group first described an alternate CIC::NUTM1 fusion" - This fusion has been reported previously in literature, and the paper cited is not from the corresponding author's research group. We would appreciate clarification on the novelty of the present study specifically, what aspects of this fusion are being reported for the first time in the context of this cancer, or whether this represents the first description of the fusion gene by your group. It would be helpful if this could be clearly stated in the manuscript, for example: "Although isolated instances of this fusion have been mentioned in earlier studies, our study is the first to systematically validate and characterize this fusion in a cohort of CIC patients."
Paper reference-Clinicopathologic Features of CIC-NUTM1 Sarcomas, a New Molecular Variant of the Family of CIC-Fused Sarcomas - PMID: 30407212

3) Line 134: Fig.1C- Do you have a Western blot with a longer exposure? The bands for CIC09 and CIC01 appear somewhat faint. We recognize that some proteins and antibodies can be challenging to detect and may not always yield

strong signals in Western blot analyses.

4) Line 134-135: Fig.1C- We would appreciate clarification on the observed expression pattern, as wildtype DUX4 appears relatively consistent across samples, whereas wild-type NUTM1 shows greater variability. Is there a biological or technical explanation for this difference, or is this the expected expression pattern, or does this effect the biology of this cell line in ways that might influence interpretation of the data presented here?

5) Line 145: Fig.1D- Although high MYC expression is often associated with RB loss or inactivation, RB protein can sometimes remain detectable due to hyperphosphorylation. Given the relatively high RB levels observed in some MYC-high samples, could the authors please clarify whether this reflects phosphorylated RB or another mechanism of RB stabilization?

6) There are several issues with the dose response curves that need to be fixed.
a. For all of the compound dosing data presented the IC50 is presented as a single number, but this does not capture the variability present in data with technical replicates or the uncertainty that comes from performing a nonlinear regression to fit a curve. I went to the tool listed in the methods section and entered some data from a dose-response curve in our lab. It is clearly fitting to the curve to the right equation, and somewhat obliquely reports the maximum, minimum, Hill slope, and IC50, but it is not reporting any measures that allow you to capture the uncertainty inherent in your measurement. IC50s should have 95% confidence intervals reported with them, and you should evaluate the actual curve fit by looking at QC measures such as the R^2 value. These are readily available when analyzing this type of data in GraphPad, which appears to be the software used to generate plots. The 95% CIs should be included in Supplementary Table S1.
b. Given that the plots are made in GraphPad, it is unclear if the curves presented are also from a nonlinear regression and if the values there match the values presented in the text and Supplementary Table S1. This is particularly important, because some of the curves presented (Panels 2I, 4A-B, S5A-B) appear to show a curve that has not turned over to a minimum value yet, which would indicate that those data are fit to a model with negative cell viability, which is physically impossible. When fitting in GraphPad, you are able to constrain your curve fit with a minimum value that remains at or above 0.
c. In panel 2I, this is not the typical way to report resistance. You should evaluate an IC50 for each condition and compare them and whether their 95% CI overlap. Here, there is not quite enough data to be confident in your curve fits of an IC50 for either condition. Somewhat related, do the authors have any idea about the sgControl looks to have a higher IC50 that the parental cell line along in panel 2A?

7) Can the data from 2G be quantified with image analysis?

8) In discussing the data presented in Panel 2H, CDK2 is hardly knocked down. The authors stated that cells die at later timepoints (14 d) making it hard to assess CDK2 levels, but is there any data to show CDK2 depletion at intermediate timepoints, particularly for sgCDK2 #2? Also, the data in 2G indicate that there are cells in CIC02 and CDS2 that could be assayed at 14 days.

9) The data presented in 2K should include a more robust analysis of quantified, or semi-quantification of multiple high-magnification fields for each condition, or at least some additional replicates if possible. A single image of a slide is not sufficient.

10) The transcriptomic analysis has several issues as outlined below
a. For panel 3A, these data need to be presented with the hierarchical clustering tree shown to properly evaluate how samples are clustering. Simply being next to each other vertically is not sufficient to say they cluster without showing the branching. This is particularly important for the left subpanel of panel 2A.
b. There is no way to gauge differential gene expression without the inclusion of a legend showing what the colors represent for these heatmaps.
c. The data from Enrichr in panel 3B need to presented with some sort of scale with each sub panel. I see that there are data presented in Supplementary Table S2, however, when I plug in the same genes to Enrichr I get qualitatively the same results, but with slightly different values. Can you discuss which program version you performed these analyses with to improve transparency and reproducibility in reporting bioinformatics analyses?
d. Likewise, a more complete description of the data would report the differentially expressed genes (both up and down) in a separate supplementary table, and discuss how gene lists were curated for analysis with Enrichr. For example, for the CIC02 cell line, I see that 4 genes were the input for Enrichr, but there were 11 differentially expressed downregulated genes detected in the differential expression analysis. Why were only 4 genes chosen? They are not the 4 common between both cell lines. Likewise, out of 965 downregulated genes for CIC01, why are only 10 used for Enrichr input? Pathway analysis should be performed using the whole set of differentially expressed genes, unless there is a cutoff being used that is not reported. If there is a separate cutoff performed from what is reported on Line 564.
e. Related to comment 5d, why did authors choose to present the Transcription Factor PPI library from Enrichr from all of the possible enrichment options? I believe this means that enriched transcription factors are likely interactors with the genes listed in the gene set, as opposed to transcription factors that are regulated by the differentially expressed genes or transcription factors that control expression of the differentially expressed genes, either of which may be more interesting.
f. Given all of the issues with the transcriptomic analysis discussed, the subsection title on line 209 and the figure legend title for figure 3 both indicating that tagtociclib inhibits MYC transcript expression and MYC oncogenic programs are highly misleading. That is not what this transcriptomic analysis shows. The data presented here indicate that the differentially expressed genes are known to interact with MYC protein, not that the compound is having any effect on MYC transcript level. The data needed to support these titles would include an analysis of MYC mRNA expression or GSEAs looking at MYC target gene expression in the context of CDK2 inhibition. Alternatively, the titles of these sections should be changed

to more accurately reflect the transcriptomic data presented.

11) Line 219-224 (Results) – It was unclear how this information was connected to the results shown for figure 3 or any other relevant discussion of MYC. Is this information perhaps more suited to the Discussion section?

12) Line 259: (Results) - Is MYC expression uniformly high across all CIC-rearranged sarcomas? Have the authors evaluated MYC-nonamplified CIC sarcoma models (if any, figure 6 suggests that there are MYC-nonamplified CIC-rearranged sarcomas)? If so, could they clarify whether the mechanism of action of CDK1/2/9 inhibitors differs in those settings compared with the models described in this study?

13) Line 292: Fig 6A and 6B-The MYC amplification findings are intriguing, and we would welcome additional detail to better understand this observation. Specifically, could the authors clarify how many MYC copies are gained, and the nature of the amplification (e.g., extrachromosomal versus homogeneously staining regions, or another mechanism)? If FISH data are available, representative images would be especially valuable and would nicely complement the existing results.

14) Related to Figure 6, while these data are intriguing, would there be a way to include discussion of these results earlier in the paper, perhaps more connected to description of clinically available data or characteristics of the patient-derived models. As presented, this doesn't feel particularly well connected to the rest of the paper.

15) Line 343: There is an “in” missing after the word “sensitivity”.

Version 1:

Reviewer comments:

Reviewer #1

(Remarks to the Author)
The authors have addressed all my concerns. They have elevated the mechanistic and preclinical impact of their findings.

Reviewer #2

(Remarks to the Author)
The authors have adequately addressed my concerns.

Reviewer #3

(Remarks to the Author)
The authors have adequately addressed my comments and the manuscript should be accepted for publication.

Open Access This Peer Review File is licensed under a Creative Commons Attribution 4.0 International License, which

permits use, sharing, adaptation, distribution and reproduction in any medium or format, as long as you give appropriate credit to the original author(s) and the source, provide a link to the Creative Commons license, and indicate if changes were made.
In cases where reviewers are anonymous, credit should be given to 'Anonymous Referee' and the source.
The images or other third party material in this Peer Review File are included in the article's Creative Commons license, unless indicated otherwise in a credit line to the material. If material is not included in the article's Creative Commons license and your intended use is not permitted by statutory regulation or exceeds the permitted use, you will need to obtain permission directly from the copyright holder.
To view a copy of this license, visit https://creativecommons.org/licenses/by/4.0/

Inga-Marie Schaefer, MD
Associate Pathologist
Department of Pathology
Brigham and Women's Hospital
75 Francis Street
Boston, Massachusetts 02115-6110

Assistant Professor of Pathology
Harvard Medical School
Tel: (617) 525-7447
Fax: (617) 277-9015
ischaefer@bwh.harvard.edu

June 2, 2026

RE: COMMSMED-25-3066A - "Co-targeting CDK2 and CDK9 inhibits MYC oncogenic programs and has anti-tumor effects in *CIC*-rearranged sarcoma"

Thank you for the opportunity to submit a revised version of our manuscript "Co-targeting CDK2 and CDK9 inhibits MYC oncogenic programs and has anti-tumor effects in *CIC*-rearranged sarcoma". We are grateful to the reviewers for their careful evaluation and constructive comments, which have helped us strengthen the manuscript. In response to their suggestions, we have clarified key points and added additional calculations and figures to enhance the interpretation and presentation of our findings.

We appreciate the opportunity to revise our work and provide detailed responses to each comment below (changes marked in red).

REVIEWER COMMENTS

Reviewer 1:

Odom-Lewis et al., report on their preclinical studies that further define a targetable dependency in CIC-rearranged sarcoma. Specifically, they leverage prior studies that demonstrate a dependence on CDK2-cyclin E complex in CIC::DUX4 models but now expand these findings into other non-CIC::DUX4 tumors including CIC::NUTM1 for which they generate a new cell-based model system. The findings of this study are of importance due to the paucity of effective treatments for CIC-rearranged cancers but also due to the lack of clinical advancement of CDK2 inhibitors in the late-phase space. Thus, the aim of this study was to identify the key CDKs that promote survival and to leverage this insight to refine the therapeutic approach that limits drug toxicity of older less-specific CDK2 inhibitors such as dinaciclib.

Through their studies, they define CDK9 and CDK2 as key molecular and therapeutic targets that potentially converge on Myc in CICrearranged sarcomas. They argue that the therapeutic effect of targeting CDK2 can be augmented with combinatorial approaches using drugs that indirectly suppress MYC expression. Overall, this is a well-written and straightforward preclinical study that further develops a potential therapeutic strategy for CIC-rearranged sarcoma.

- We thank Reviewer #1 for their thoughtful and encouraging evaluation of our manuscript. We appreciate the recognition of the significance and translational potential of our work, particularly in the context of limited therapeutic options for *CIC*-rearranged sarcomas. Below, we address the reviewer's comments in detail.

Major Comments:

1. Figure 5H. The tumor growth curve in Figure 5H (CIC02 xenografts) requires correlative data including an assessment of proliferative and apoptotic markers (Cl-caspase by IHC and/or Parp-cleavage by protein expression) in the explanted tumors to support their claim that the observed effect is through "proliferative arrest and induction of apoptosis".

- We appreciate the reviewer's insightful suggestion. In response, we have added a new panel (Figure 6I) providing correlative analyses of the treated CIC02 PDX tumors. Specifically, we demonstrate a reduction in the proliferation marker Ki67 and induction of apoptotic cell death, along with increased expression of the apoptosis markers cleaved caspase-3 and cleaved PARP by immunohistochemistry. These additional data further support our conclusion that the observed antitumor effect is associated with both proliferative arrest and induction of apoptosis.

New Figure 6I:

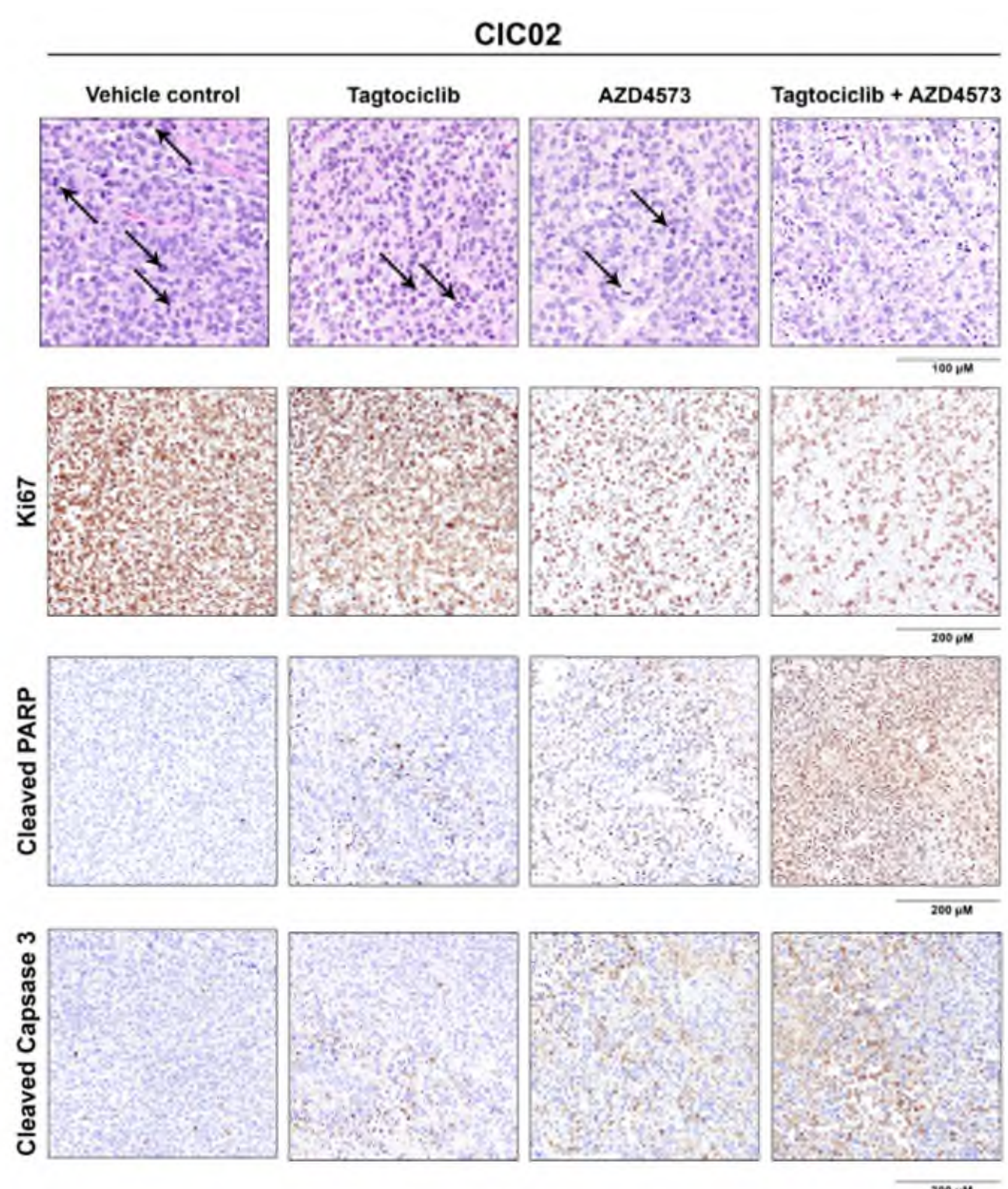

Figure 6I: Combined treatment with tagtociclib at 120 mg/kg BID PO and AZD4573 at 10 mg/kg Q2H BID 2ON 5OFF IP for 10 days eliminated mitotic activity, induced apoptotic cell death, substantially reduced the Ki67 proliferation index, and induced expression of cleaved PARP (Asp 214) and cleaved caspase 3 (Asp175) compared to single-agent tagtociclib at 120 mg/kg/d or single-agent AZD4573 at 10 mg/kg/d in CIC02 (N=1 each). Mitoses are indicated by arrows.

We have added the following paragraph to the Results section (lines: 310-314): Combined treatment with tagtociclib and AZD4573 for 10 days eliminated mitotic activity, induced apoptotic cell death, substantially reduced the Ki67 proliferation index, and induced expression of cleaved PARP (Asp 214) and cleaved caspase 3 (Asp175) compared to single-agent tagtociclib or single-agent AZD4573 in CIC02 (**Fig. 6I**).

2. Since a major point in the manuscript is the dependence on MYC, it is important to at least attempt to define a mechanism by which MYC is regulated in CIC-rearranged cells. Thus, please attempt to rescue MYC expression using a MYC ser62 mutant isoform in at least one tagtociclib treated CIC-rearranged cell line to further define the mechanism of MYC regulation (post-tranlational vs transcriptional).

- We thank the reviewer for this thoughtful and important suggestion. To further investigate the mechanism underlying MYC regulation in *CIC*-rearranged sarcoma cells following CDK2 inhibition, we performed several additional studies and incorporated the resulting data into the revised manuscript.

  First, to strengthen our findings regarding therapeutic CDK2 inhibition and MYC regulation, we added a new immunoblot analysis using the selective CDK2 inhibitor BLU-222 (Supplementary Figure S2D). Consistent with our prior findings using tagtociclib (Figure 2D), BLU-222 treatment reduced phospho-Rb (Ser795), phospho-MYC (Ser62), and total MYC protein levels in both CIC01 and CIC02 cell lines.

New Supplementary Figure S2D:

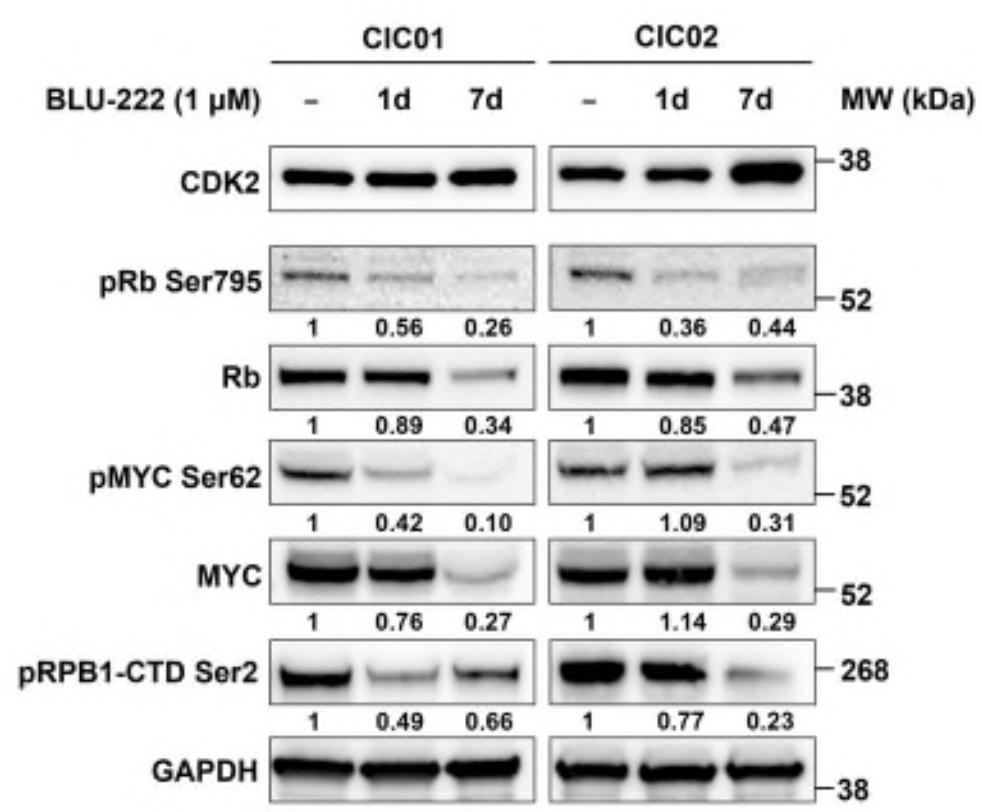

Supplementary Figure S2D: Immunoblotting analyses show that BLU-222 treatment inhibits the expression of phospho-Rb Ser795, phospho-MYC Ser62, total MYC, and phospho-RPB1-carboxyl-terminal domain (CTD) Ser2 in the CIC01 and CIC02 cell lines. This effect is more pronounced after 7 days of treatment than after 1 day of treatment. CDK2 expression remains unchanged. The calibrated expression is shown at the bottom of each row. GAPDH was used as the loading control.

  To further address whether MYC regulation occurs at the transcriptional or post-translational level, we performed additional mechanistic studies. As shown in newly added Figure 4B, tagtociclib treatment did not suppress MYC transcription, suggesting that the observed reduction in MYC expression is not primarily mediated at the transcriptional level. We next examined the effects of tagtociclib in combination with cycloheximide, an inhibitor of protein translation. As shown in newly added Figure 4F, combined treatment with tagtociclib and cycloheximide produced an additive reduction in MYC protein expression, supporting a model in which tagtociclib primarily reduces MYC stability rather than inhibiting MYC translation.

  Together, these findings provide additional mechanistic insight into MYC regulation in *CIC*-rearranged sarcoma cells following CDK2 inhibition. We have also incorporated these

results into the newly added schematic model presented in Figure 6J (see also our response to Minor Comment #1).

Revised Figure 4 (*formerly 3*):

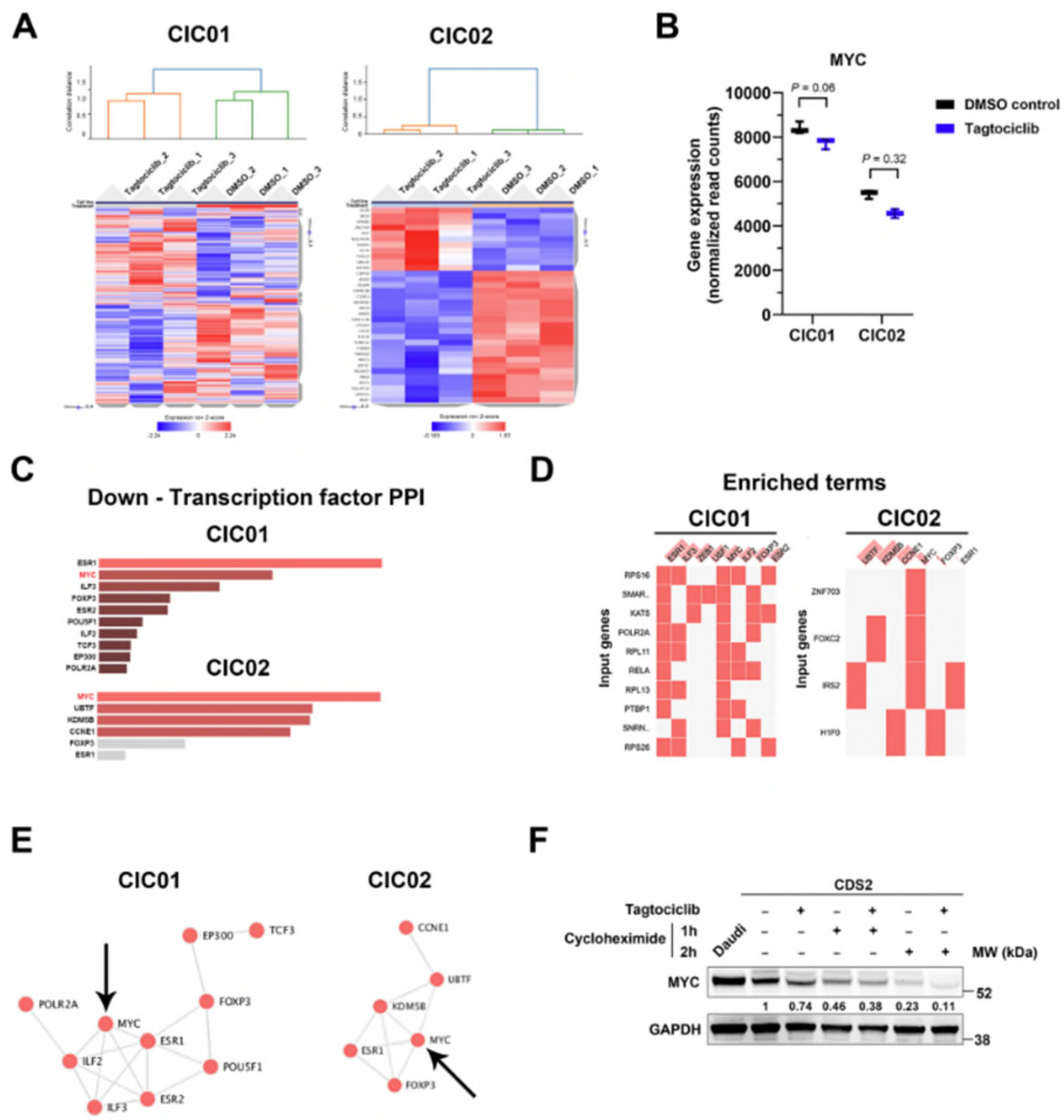

Fig. 4. Tagtociclib inhibits MYC oncogenic programs and reduces MYC protein stability.
(A) RNA sequencing analysis was performed in triplicate in the CIC01 and CIC02 cell lines after treatment with tagtociclib at 200 nM for 48 hours. Unsupervised hierarchical clustering demonstrates that tagtociclib treated cells separated from DMSO-control within each cell line. **(B)** Tagtociclib treatment did not suppress MYC transcript expression in CIC01 and CIC02. **(C)** Analysis of the transcription factor protein-protein interactions (PPI) library demonstrates that tagtociclib treatment results in downregulation of the MYC transcription factor signature in CIC01 and CIC02 indicating inhibition of genes encoding proteins that interact with MYC. Signatures are ranked by *P* value. Red bars indicate $P \leq 0.05$; gray bars indicate $P > 0.05$. **(D)** Heatmap showing the genes downregulated by tagtociclib treatment in CIC01 and CIC02 that contribute to the MYC transcription factor signature. **(E)** Functional networks for the MYC transcription factor signature highlighting MYC and other genes significantly downregulated by tagtociclib treatment in CIC01 and CIC02. **(F)** Combined treatment with tagtociclib at 1 µM for 6 days and cycloheximide at 178 µM for 1 or 2 hours produced an additive reduction in MYC protein expression, supporting a model in which tagtociclib primarily reduces MYC stability rather than inhibiting MYC translation.

Accordingly, we revised the heading of the Results section and included our new observations (lines: 235-256):

Tagtociclib inhibits MYC oncogenic programs and reduces MYC protein stability.
To determine the effects of CDK2 inhibition at the transcriptomic level, we performed RNA sequencing analysis of the CIC01 and CIC02 cell lines in response to tagtociclib.

Unsupervised hierarchical clustering demonstrated that cells treated with tagtociclib were separated from DMSO-control within each cell line (**Fig. 4A**). All significantly differentially expressed genes identified in CIC02 and CIC01 were included in the enrichment analyses. *CCNE1* and *CCNE2* were among the 11 most significantly upregulated genes common in CIC01 and CIC02 cells in response to tagtociclib treatment (**Supplementary Fig. S4**), indicating compensatory cyclin E upregulation upon CDK2 inhibition. Tagtociclib treatment did not suppress MYC transcription (**Fig. 4B**) suggesting that the observed reduction in MYC expression and downregulation is not primarily mediated at the MYC transcriptional level. However, analysis of the transcription factor protein-protein interactions (PPI) library uncovered that tagtociclib treatment of CIC01 and CIC02 cells resulted in downregulation of genes encoding proteins that interact with MYC, indicating an indirect effect of tagtociclib treatment on MYC signatures (**Fig. 4C-E, Supplementary Table S2**). We next examined the effects of tagtociclib on MYC protein stability using cycloheximide, a strong inhibitor of protein translation, to assess protein turnover. Combined treatment with tagtociclib and cycloheximide produced an additive reduction in MYC protein expression (**Fig. 4F**), supporting a model in which tagtociclib primarily decreases MYC stability rather than inhibiting MYC transcription. Together, these findings suggest that CDK2 inhibition in *CIC*-rearranged sarcoma cells downregulates MYC transcriptional activity by reducing MYC protein stability.

We added the following sections to the Discussion (lines: 343-345 and 402-409): Interestingly, our studies also demonstrated that CDK2 inhibition and *CDK2* knockout resulted in downregulation of MYC expression through decreased MYC protein stability rather than inhibition of MYC transcription (**Fig. 6J**). [...] In summary, our study highlights *CIC*-rearranged sarcoma as a MYC-dependent cancer and demonstrates anti-tumor effects of concurrent inhibition of CDK2 and CDK9 through proliferative arrest and induction of caspase 3-mediated apoptotic cell death in preclinical models of *CIC*-rearranged sarcoma. We further identify distinct but complementary mechanisms of MYC regulation, whereby CDK9 inhibition suppresses MYC transcription and CDK2 inhibition impairs MYC protein stability. Our findings provide key preclinical data that could serve as a rationale for clinical trials evaluating CDK2 and CDK9-targeted therapies in patients with *CIC*-rearranged sarcoma.

3. While it is appreciated that the MYC-MAX dimerization inhibitors decrease viability (Supplmental Figure 5) in all CIC-rearranged cell lines it is important to include a proper MYC-independent cell line for control.

- We appreciate the reviewer's thoughtful suggestion to include a MYC-independent cell line as an additional control for the MYC-MAX inhibitor experiments shown in Supplementary Figure S5. In response, we have added U2-OS, an osteosarcoma cell line that is well established as a bona fide MYC-independent model, and therefore serves as an appropriate negative control for assessing the specificity of MYC-MAX inhibition. The updated data are now included in Supplementary Figure S5.

Revised Supplementary Figure S5:

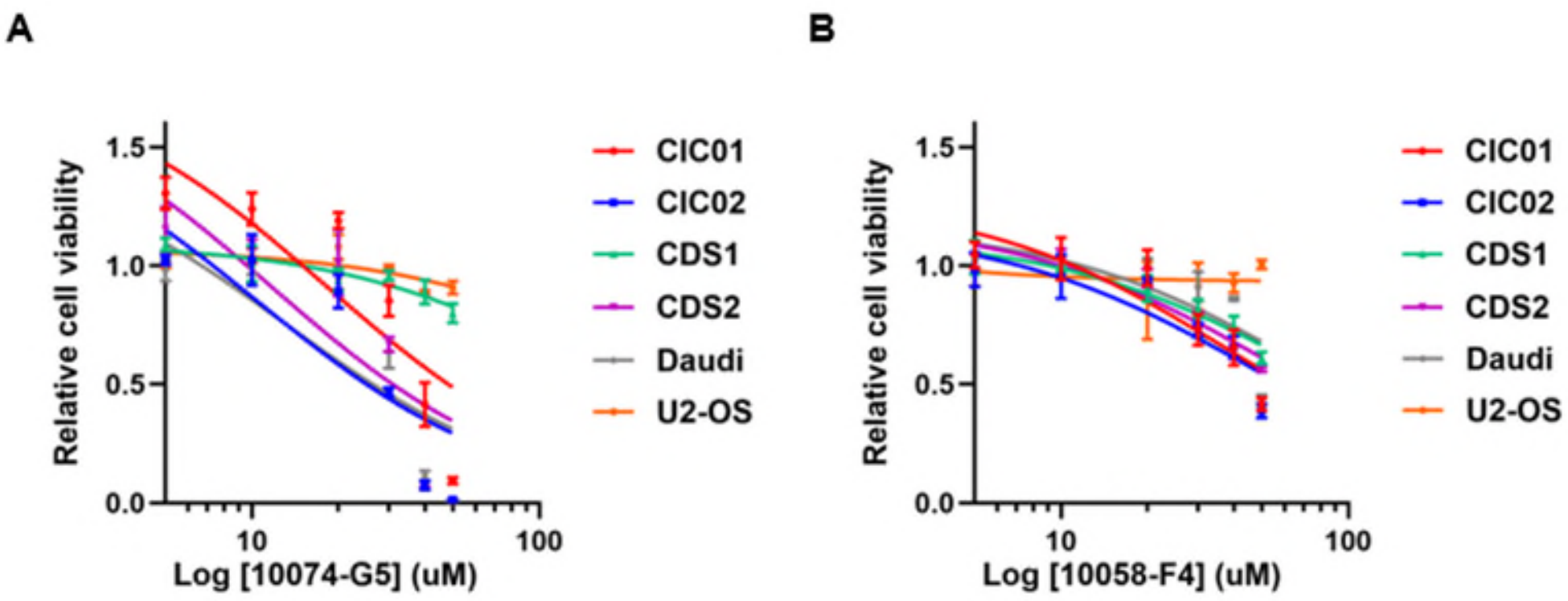

Supplementary Figure S5. Direct MYC inhibition has anti-tumor effects in *CIC*-rearranged sarcoma. The MYC-MAX dimerization inhibitors 10074-G5 **(A)** and 10058-F4 **(B)** inhibit cell viability as assessed by CellTiter-Glo assay in the CIC01, CIC02, CDS1, and CDS2 cell lines as well as in Daudi control but not in U2-OS (normalized to DMSO). Tests were performed in triplicate (mean +/- s.d).

Minor Comments:

1. A summary schematic/cartoon figure that highlights the key CDKs and how they interact with MYC and CIC fusions would help the reader understand this rather complex pathway.

- We appreciate the reviewer's thoughtful suggestion. In response, we have added a new schematic figure (Figure 6J) illustrating the key roles and interactions of CDK2 and CDK9 in relation to MYC signaling and *CIC* fusion-driven sarcoma biology. We hope this summary diagram helps clarify the pathway and improves the overall accessibility of this complex mechanism for readers.

New Figure 6J:

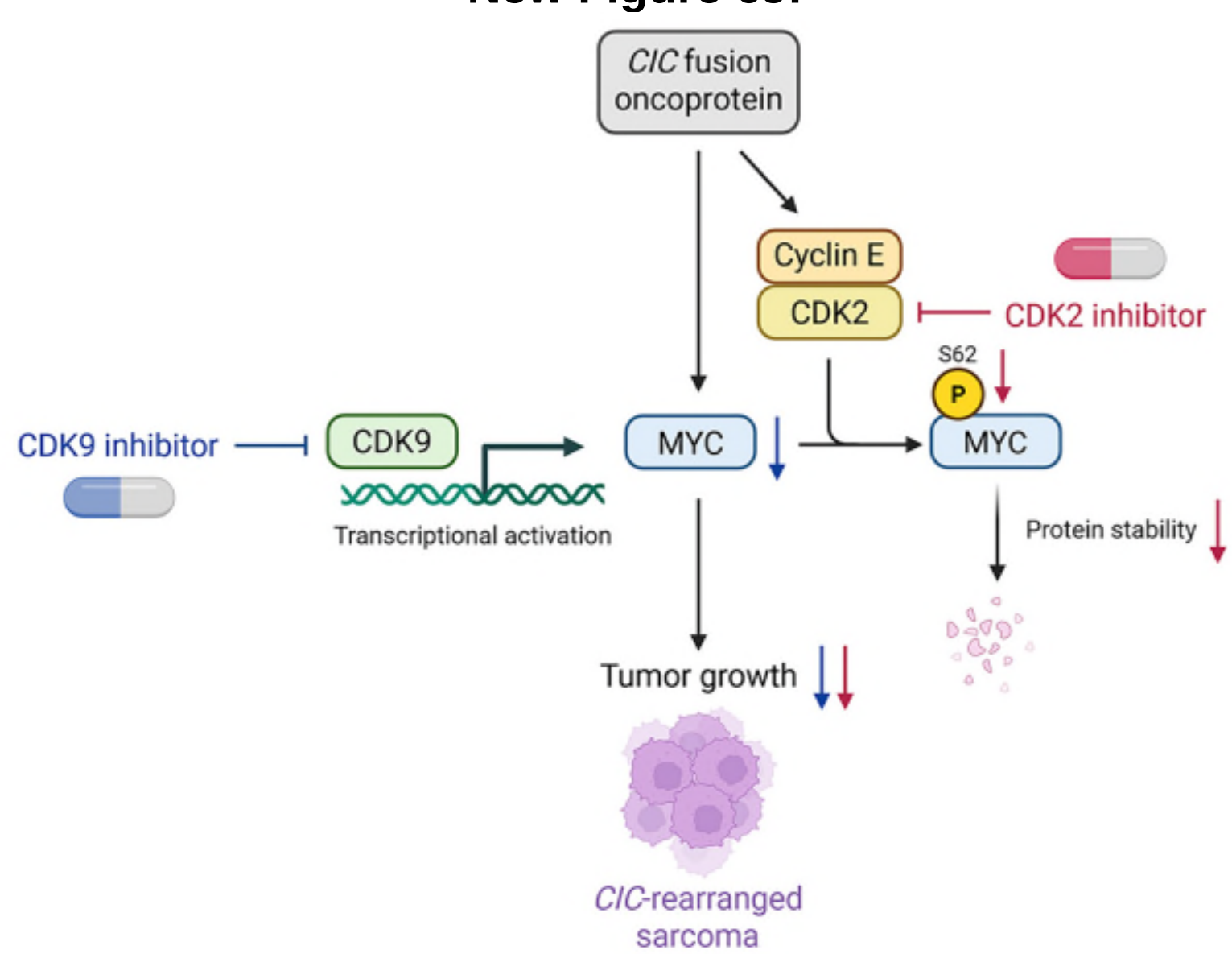

Figure 6J: Schematic illustrating the key roles and interactions of CDK2 and CDK9 in relation to MYC signaling and *CIC* fusion-driven sarcoma biology.

We have added the following to the Results section (lines: 314-318): Together, these results demonstrate that concurrent CDK2 and CDK9 inhibition has greater anti-tumor effects in preclinical models of *CIC*-rearranged sarcoma than CDK2 or CDK9 inhibition alone through combined proliferative arrest and induction of apoptosis via CDK9-mediated inhibition of MYC transcription and CDK2-mediated impairment of MYC stability (**Fig. 6J**).

2. Please expand the discussion to speculate why tagociclib treatment is less effective than CDK2 knockout? Is this the result of incomplete inhibition of CDK2 by tagociclib? Alternatively, are there off-target effects from tagociclib that enable partial survival ?

- We thank the Reviewer for this insightful comment. The observed difference in therapeutic CDK2 inhibition versus *CDK2* knockout may reflect dosing and specificity of tagtociclib, as well as potential off-target activity of CDK2 inhibitors on CDK1 and CDK9 or a combination of these. Additionally, the possibility that incomplete knockout efficiency as evidenced by residual CDK2 expression may contribute to the phenotype we observed. We added a paragraph to the Discussion (lines: 333-338): The observed difference in therapeutic CDK2 inhibition versus *CDK2* knockout may reflect dosing and specificity of tagtociclib, as well as potential off-target activity of CDK2 inhibitors on CDK1 and CDK9 or a combination of these. Additionally, the possibility that incomplete knockout efficiency as evidenced by residual CDK2 expression may contribute to the phenotype we observed.

3. Please reference the following studies that demonstrate increased CCNE levels in CIC::DUX4 tumors and thus lends additional support to the authors findings: PMID: 38703529, PMID: 38705279

- We appreciate the reviewer's suggestion and have included the two suggested reference in the Results (lines: 169-172): Based on previous reports indicating molecular dependence on the CDK2-cyclin E pathway[16] along with high cyclin E expression[17, 18] and frequent genomic gain of MYC, a regulator of CDK2-cyclin E[19], we first assessed the CDK2-cyclin E pathway as a potential therapeutic vulnerability in our *CIC*-rearranged sarcoma models.

  17. Karabulut B, Yukruk FA, Yenidunya S, Kandemir O, Kosemehmetoglu K. Differential cyclin-E1 expression in CIC-rearranged sarcoma. Ann Diagn Pathol 72, 152320 (2024).

  18. Doll S, et al. Proteomic Characterization of Undifferentiated Small Round Cell Sarcomas With EWSR1 and CIC::DUX4 Translocations Reveals Diverging Tumor Biology and Distinct Diagnostic Markers. Mod Pathol 37, 100511 (2024).

Reviewer 2:

In this paper, the authors characterize two new PDX/cell line models of CIC rearranged sarcomas and address therapeutic targeting. Although the reported findings do support the idea of targeting CDK2 and CDK9 in the treatment of this disease, several issues should be addressed.

- We thank Reviewer #2 for their thoughtful evaluation of the manuscript. They appreciate the recognition of the value of the newly developed PDX and cell line models, as well as the potential of CDK2 and CDK9 as therapeutic targets in *CIC*-rearranged sarcomas. We authors address the reviewer's comments and concerns in detail below.

1. Since the patients from whom the new cell lines were derived have metastatic disease, it would be great to know if the sample was obtained from the primary tumor or from a metastasis. If the latter, where was it? And were these samples collected before or after administration of chemotherapy?

- We appreciate the reviewer’s suggestion and have included details on the origin of the patient tumors that our cell lines and PDXs were established from and any prior treatment in the Results (lines: 131-138): CIC01 was derived from a lung metastasis in a 25-year-old female who had a perineal primary tumor resected 10 months prior and had been treated with adjuvant chemotherapy consisting of vincristine, Adriamycin/doxorubicin, cyclophosphamide, ifosfamide, and etoposide (VDC/IE) as well as radiation to the perineum and pelvis. The tumor had also metastasized to the brain. CIC02 was derived from a locally recurrent tumor involving spleen, peritoneum, psoas muscle, and colon in 39-year-old male who had a primary kidney tumor resected 6 months prior and had been treated with adjuvant chemotherapy (VDC/IE).

2. The data in Figure 1 demonstrate that the anticipated defining molecular features are present, much more work (for example, RNA-seq comparisons, global epigenomic profiling, etc) would be needed to validate the statement that these cell lines and PDXs “closely resemble” the primary tumors.

- We agree with the reviewer and have rephrased our statement in the Results accordingly to omit the word "closely" (lines: 165-168): Together, these results indicate that the CIC01 and CIC02 cell lines and PDXs ~~closely~~ resemble the primary tumors from which they were established and represent additional preclinical models for subsequent *in vitro* and *in vivo* analyses, including the first-ever model of *CIC::NUTM1*-rearranged sarcoma.

3. Most cancer cells express Cyclin E and cdk2, and the mere presence of retained RB1 or high level protein expression of MYC are not evidence of molecular dependence or an oncogenic driver.

- We thank the reviewer for this important comment. We agree that the presence of Cyclin E/CDK2 expression, retained Rb, or elevated MYC levels alone does not establish molecular dependence or define an oncogenic driver. To address this, we emphasize that functional evidence is critical. In our *CIC*-rearranged sarcoma models, both pharmacologic inhibition and genetic knockout of *CDK2* result in a marked proliferative arrest. These findings support a functional dependence on CDK2 activity in this context, rather than reliance on expression levels alone.

4. The experiments in Figure 2 lack appropriate negative controls. Almost any rapidly proliferating cell line would be expected to slow down when treated with an inhibitor of a key mediator of cell cycle progression, like CDK2. If the authors wish to prpose that this is somehow aunique characteristic of CIC rearranged sarcomas, then either showing lack of efficacy of an inhibitor of a different cyclin-dependent kinase or lack of effect of tagtociclib on a related sarcoma would be necessary. This criticism applies to both inhibitor and KO experiments.

- We appreciate the reviewer's suggestion to show lack of efficacy of an inhibitor of a different cyclin-dependent kinase in *CIC*-rearranged sarcomas. As shown in Supplementary Figure S3B and E, the CDK4/6 inhibitor palbociclib had no effect on cell proliferation or viability in any of the four *CIC*-rearranged sarcoma cell lines tested, supporting that *CIC*-rearranged sarcomas are uniquely sensitive to CDK2 inhibition but not to CDK4/6 inhibition:

Supplementary Figure S3:

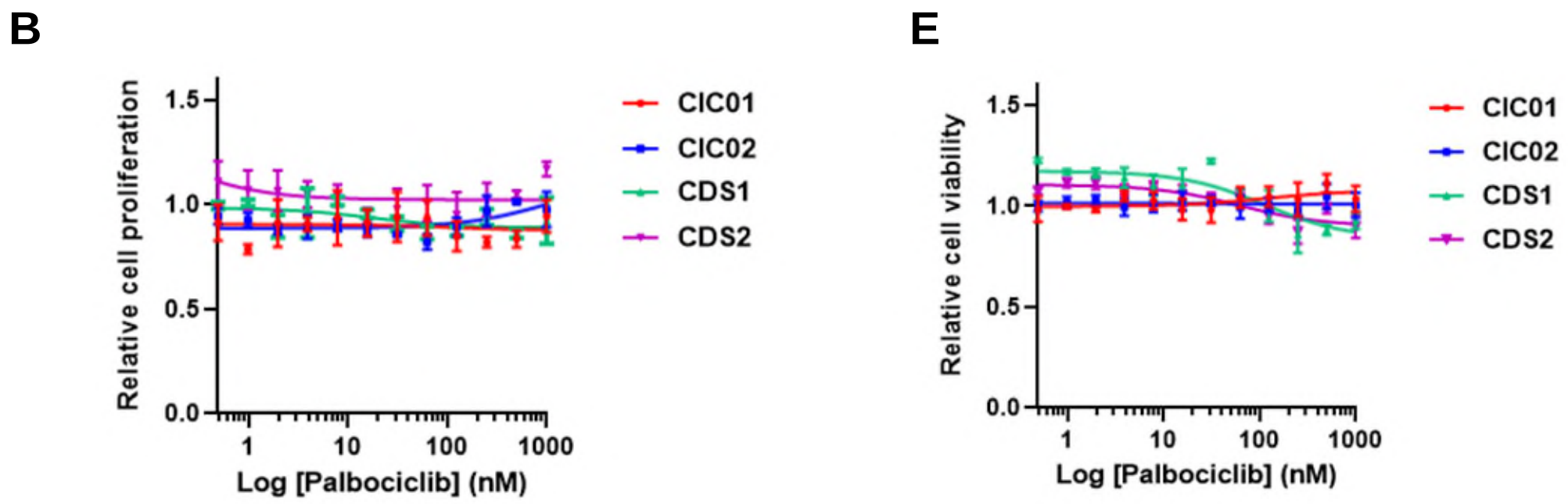

5. Similarly, experiments in Figure 4 would be more convincing that CDK2 and CDK9 are somewhat unique vulnerabilities of this tumor type if appropriate negative controls (for example, another small round blue cell sarcoma) were included to demonstrate that this approach works for CIC rearranged sarcomas and not for others.

- We appreciate the reviewer's suggestion to include a MYC-independent cell line as control for the CDK9 inhibitors shown in Figure 5 (*formerly 4*). In response, we have added U2-OS, an osteosarcoma cell line that is well established as a bona fide MYC-independent model, and therefore serves as an appropriate negative control for assessing the specificity of CDK9 inhibition. The updated data are now included in Figure 5A-F (see also Supplementary Figure S5):

Revised Figure 5A-F (*formerly 4A-F*):

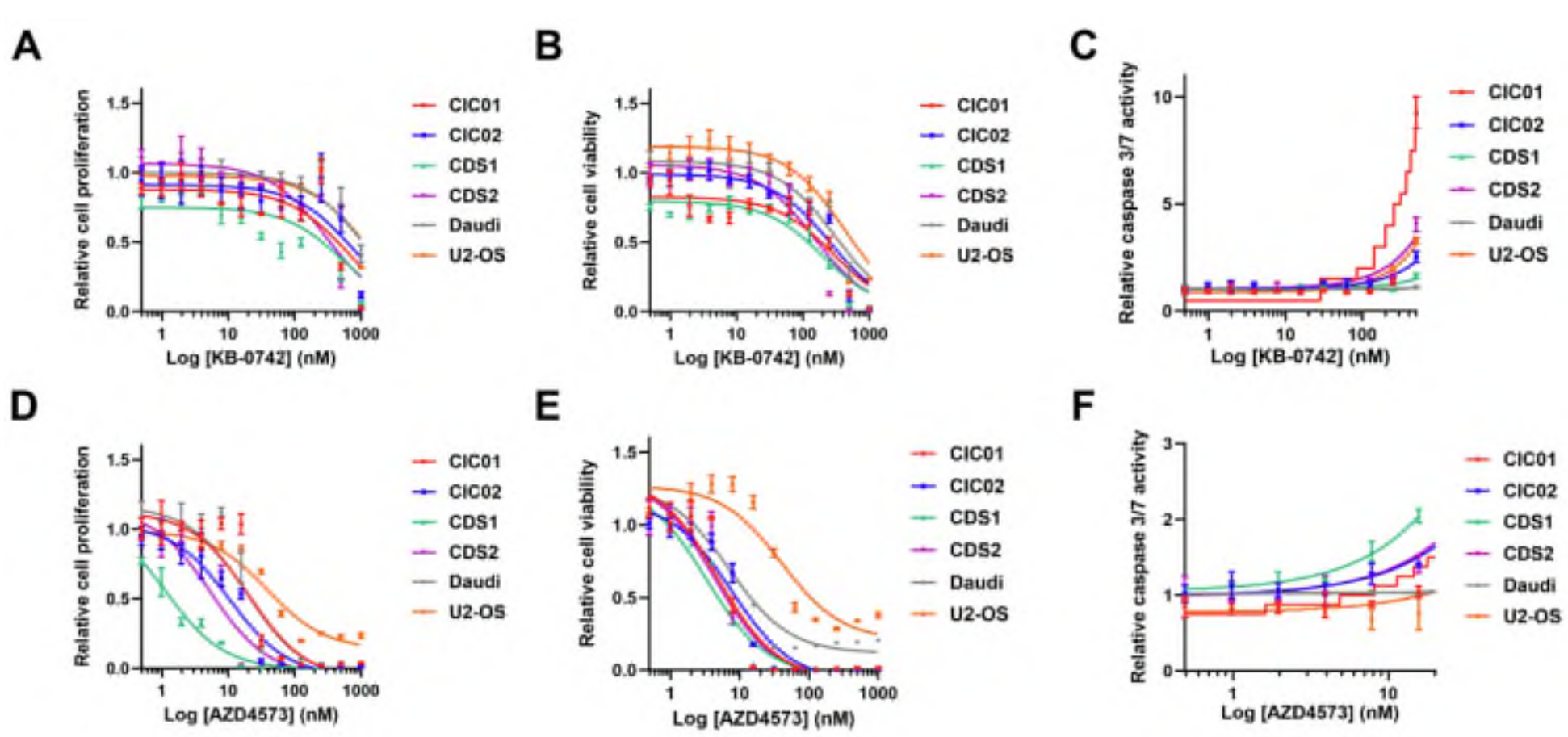

Fig. 5: KB-0742 inhibits cell proliferation as assessed by BrdU incorporation at 24 h **(A)** and cell viability as assessed by CellTiter-Glo assay **(B)** in CIC01, CIC02, CDS1, and CDS2. KB-0742 also induces apoptosis in CIC01, CIC02, CDS1, and CDS2 as measured by Caspase-Glo 3/7 assay. Tests were performed in triplicate (mean +/- s.d) **(C)**. AZD4573 inhibits cell proliferation as assessed by BrdU incorporation at 24 h **(D)** and cell viability as assessed by CellTiter-Glo assay **(E)** in CIC01, CIC02, CDS1, and CDS2. AZD4573 also induces apoptosis in CIC01, CIC02, CDS1, and CDS2 as measured by Caspase-Glo 3/7 assay. Tests were performed in triplicate (mean +/- s.d) **(F)**. MYC-independent U2-OS cells were substantially less sensitive to either treatment (**A-F**).

We added these findings to the Results (lines: 267-273): Our cell-based assays demonstrated that KB-0742 inhibited cell proliferation and cell viability and increased apoptosis in CIC01, CIC02, CDS1, and CDS2 (**Fig. 5A-C**), with proliferation IC_{50} values ranging from 304.1-744.4

nM (**Supplementary Table S1**). Similarly, treatment with AZD4573 inhibited cell proliferation and cell viability and increased apoptosis in CIC01, CIC02, CDS1, and CDS2 (**Fig. 5D-F**), with proliferation IC_{50} values ranging from 1.16-20.49 nM (**Supplementary Table S1**). U2-OS cells were substantially less sensitive to either treatment (**Fig. 5A-F**).

We also included the proliferation IC_{50} values in Supplementary Table S1:

Revised Supplementary Table S1:

Proliferation IC_{50} values of CDK2 and CDK9 inhibitor drugs in *CIC*-rearranged sarcoma cell lines.

	IC_{50} value [nM] (95% CI)					
Cell line	Tagtociclib	KB-0742	AZD4573	BLU-222	PF-06873600	Palbociclib
CIC01	71.32 (52.17-97.74)	589.6 (325.4-760.3)	20.49 (14.56-28.56)	12.88 (9.103-18.09)	39.12 (30.02-50.99)	Not reached
CIC02	45.6 (35.50-58.33)	744.4 (499.7-1089)	10.56 (8.110-13.65)	33.74 (23.29-48.98)	34.39 (26.23-45.03)	Not reached
CDS1	125.4 (89.91-176.8)	524.6 (238.3-935.9)	1.16 (0.8552-1.539)	15.86 (11.97-20.95)	3.505 (1.101-10.68)	Not reached
CDS2	23.91 (10.20-54.42)	304.1 (197.3-349.5)	5.406 (3.813-7.555)	369.3 (177.4-896.6)	12.57 (8.960-17.48)	Not reached
Daudi	824.7 (402.0-2444)	1066 (548.3-1392)	18.65 (13.60-25.53)	-	-	-
U2-OS	-	1187 (919.0-1494)	38.93 (31.26-48.4)	-	-	-

Reviewer #3:

In this manuscript, Odom-Lewis, et. al. establish new CIC-rearranged sarcoma models (cell lines and patient-derived xenografts [PDXs]) and use these models to explore the biology and therapeutic vulnerabilities in these tumors. They find that CIC-rearranged tumors frequently contain MYC amplification and a dependence on CDK2. Both pharmacological and genetic approaches to target CDK2 reduce MYC levels, impair cell cycle progression, proliferation, and change gene expression. Blocking CDK2 pharmacologically with tagtociclib also slows PDX growth in vivo. The authors further demonstrate reduced cell viability following inhibition of the MYC-MAX interaction and CDK9. Notably, CDK9 inhibitors induce apoptosis with cleaved caspase demonstrated. CDK2 and CDK9 inhibition are shown to synergistically reduce proliferation, cell viability, and in vivo xenograft growth. Together, this is an interesting manuscript, these are promising results, and this study is appropriate for publication in Communications Medicine, pending completion of revisions outlined below.

There are several strengths of this paper, including the curation of a small panel of patient proximal models that clearly capture some heterogeneity in fusions and response to treatment presented here, as well as analysis of clinical samples to investigate genomic gains of ETV4 and MYC. The authors do a good job of highlighting differences in how their cell lines respond to treatment where it is relevant. Another strength is the use of a diverse set of pharmacological compounds with varying degrees of target specificity to confirm results, coupled with genetic approaches for CDK2 depletion to validate the pharmacological findings. The xenograft data are compelling and the writing is generally clear and straightforward. The transcriptomic analysis and its presentation is a major weakness in this paper, and there are several additional weaknesses related to the presentation of the data described that need to be addressed before this manuscript can be published. See our comments below.

- We thank Reviewer #3 for their thorough and constructive evaluation of our manuscript. We greatly appreciate the recognition of the strengths of our study, including the development of

patient-relevant models, the integration of pharmacologic and genetic approaches, and the potential therapeutic implications of targeting CDK2 and CDK9. We also appreciate the reviewer's thoughtful feedback on areas for improvement, and we address these comments in detail below.

1) Line 61 (Introduction)-The authors included helpful details on the tumor type, age range, and fusion proteins. It would be great to also provide a brief, expanded explanation of CIC rearranged tumors, since they are rare. It may be useful to mention the typical body sites involved, common metastasis locations, any current treatment approaches and their success rates, and whether this cancer has been reported in children or in older adults over 30.

- We appreciate the reviewer's suggestion and have expanded the Introduction to include details on the age range, anatomic distribution, sites of distant metastases, current treatment approaches, and their efficacy (lines: 60-78): *CIC*-rearranged sarcoma is a rare and aggressive mesenchymal neoplasm that affects patients over a wide age range (6-81 years) with a median age of 30 years and ~20% cases affecting children.[1, 2] These sarcomas predominantly arise within soft-tissue (mostly trunk and extremities) with subsets occurring in bone or at visceral sites. Previously misdiagnosed as a type of Ewing sarcoma lacking EWSR1 rearrangement, it was discovered in 2006 that this sarcoma harbors *capicua* (*CIC*) rearrangement[3], leading to its recognition as a distinct entity in the 2020 WHO Classification of Tumors.[4] While 95% of *CIC*-rearranged sarcomas are driven by a *CIC::DUX4* fusion[5], rare cases harbor alternate fusion of *CIC* with either *NUTM1*[6, 7], *NUTM2A*[8], *FOXO4*[9], or *LEUTX*.[10] Our group had previously described, for the first time, a sarcoma with a *CIC::NUTM1* fusion in an adult patient[6] which has subsequently been reported in a smaller cases series.[7] In general, *CIC*-rearranged sarcoma shows very aggressive clinical behavior with frequent metastases that most commonly affect the lung.[1, 2] A median overall survival of 12 months and poor response to systemic therapies developed for Ewing sarcoma (such as the VDC/IE regimen comprising vincristine, Adriamycin/doxorubicin, cyclophosphamide, ifosfamide, and etoposide) and/or radiation[1, 2, 11] highlights the urgent need to generate a better understanding of the oncogenic mechanisms of *CIC*-rearranged sarcoma and identify biologic vulnerabilities for therapeutic targeting in an aggressive sarcoma for which currently no effective therapies exist.
(See also our response to Point #2, below).

2) Line 65: (Introduction) "our group first described an alternate CIC::NUTM1 fusion" - This fusion has been reported previously in literature, and the paper cited is not from the corresponding author's research group. We would appreciate clarification on the novelty of the present study specifically, what aspects of this fusion are being reported for the first time in the context of this cancer, or whether this represents the first description of the fusion gene by your group. It would be helpful if this could be clearly stated in the manuscript, for example: "Although isolated instances of this fusion have been mentioned in earlier studies, our study is the first to systematically validate and characterize this fusion in a cohort of CIC patients." Paper reference-Clinicopathologic Features of CIC-NUTM1 Sarcomas, a New Molecular Variant of the Family of CIC-Fused Sarcomas - PMID: 30407212

- We thank the reviewer for their suggestion. We have expanded the Introduction to include more details on the molecular subtypes of *CIC*-rearranged sarcoma and rephrased this paragraph to clarify that our group described a *CIC::NUTM1* for the first time in an adult patient with sarcoma (Schaefer et al. Genes Chromosomes Cancer. 2018 Sep;57(9):446-451). This paper was first-authored by me, the corresponding author, and senior-authored by Christopher French who also is a coauthor on the current manuscript. We also added the suggested reference by LeLoarer et al. (Am J Surg Pathol. 2019 Feb;43(2):268-276) to the

Introduction which reports a series of 6 *CIC::NUTM1* rearranged sarcomas. We also added two new references by Shintaro Sugita et al. for the description of *CIC::NUTM2A* and *CIC::FOXO4* rearranged sarcomas (Hum Pathol. 2017 Jul:65:225-230 and Am J Surg Pathol. 2014 Nov;38(11):1571-6) and by Kun Song et al. for the description of *CIC:: LEUTX4* rearranged sarcoma (Neuropathology. 2022 Dec;42(6):555-562) (lines: 66-70): While 95% of *CIC*-rearranged sarcomas are driven by a *CIC::DUX4* fusion[5], rare cases harbor alternate fusion of *CIC* with either *NUTM1*[6, 7], *NUTM2A*[8], *FOXO4*[9], or *LEUTX*.[10] Our group had previously described, for the first time, a sarcoma with a *CIC::NUTM1* fusion in an adult patient[6] which has subsequently been reported in a smaller cases series.[7]
(See also our response to Point #1, above).

8. Sugita S, et al. NUTM2A-CIC fusion small round cell sarcoma: a genetically distinct variant of CIC-rearranged sarcoma. Hum Pathol 65, 225-230 (2017).

9. Sugita S, et al. A novel CIC-FOXO4 gene fusion in undifferentiated small round cell sarcoma: a genetically distinct variant of Ewing-like sarcoma. Am J Surg Pathol 38, 1571-1576 (2014).

10. Song K, Huang Y, Xia CD, Zhu HQ, Wang J. A case of CIC-rearranged sarcoma with CIC-LEUTX gene fusion in spinal cord. Neuropathology 42, 555-562 (2022).

While the description of the *CIC::NUTM1* fusion is not novel, we here created the first patient-derived cell line and PDX from a patient tumor driven by this fusion, as we state in the Results (Together, these results indicate that the CIC01 and CIC02 cell lines and PDXs resemble the primary tumors from which they were established and represent additional preclinical models for subsequent *in vitro* and *in vivo* analyses, including the first-ever model of *CIC::NUTM1*-rearranged sarcoma).

We hope that these statements clarify the novelty and contributions of the current study in the context of this cancer.

3) Line 134: Fig.1C- Do you have a Western blot with a longer exposure? The bands for CIC09 and CIC01 appear somewhat faint. We recognize that some proteins and antibodies can be challenging to detect and may not always yield strong signals in Western blot analyses.

- We thank the reviewer for their comment. We have revised Figure 2C (*formerly 1C*) and replaced the images shown with longer exposures (5 min) for the NUT and DUX4 stain which we feel are more representative (see also our response to Point #4, below):

Revised Figure 2C (*formerly 1C*):

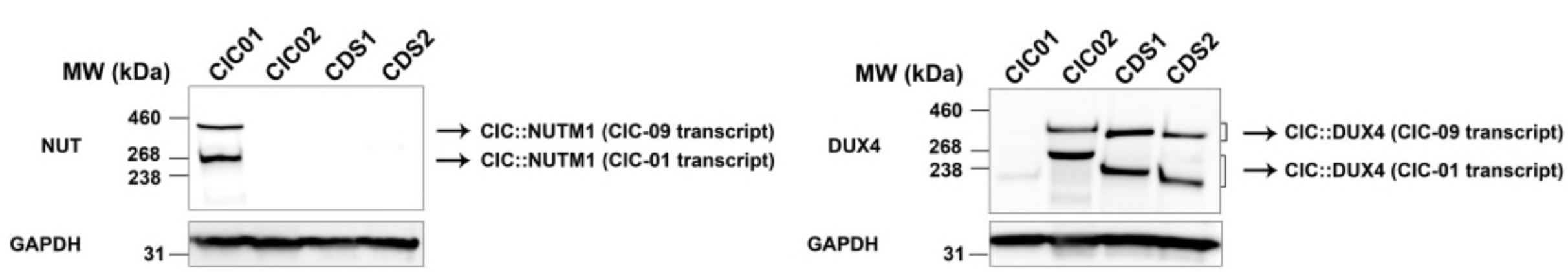

Fig. 2C: Mass spectrometry studies in CIC01 and CIC02 demonstrate the presence of CIC::NUT and CIC::DUX4 fusion oncoproteins, respectively, as visualized by immunoblotting studies. Fusion proteins appear as two bands resulting from a longer CIC-09 and shorter CIC-01 transcript.

- *4) Line 134-135: Fig.1C- We would appreciate clarification on the observed expression pattern, as wildtype DUX4 appears relatively consistent across samples, whereas wild-type NUTM1 shows greater variability. Is there a biological or technical explanation for this difference, or is this the expected expression pattern, or does this effect the biology of this cell line in ways that might influence interpretation of the data presented here?*We appreciate the reviewer's comment. We do not think that expression of wild-type NUT is markedly consequential to the biology of *CIC*-rearranged tumors based on the following reasoning. Wild-type NUT is a testis-protein whose physiologic function in post-meiotic spermatids is to acetylate histones via p300 binding, leading to nucleosome eviction via BRDT, followed by replacement with protamines. Given lack of expression of BRDT in somatic cells, wild-type NUT has no known function in these cells, and it is unusual to see it expressed (its expression in *CIC*-rearranged cell lines was unexpected). Without being fused to a nuclear chromatin-binding factor such as BRD4 or CIC, wild-type NUT is predicted to be biologically sub-functional in somatic cells, given that it requires binding to chromatin to alter it post-translationally. It is possible, however, that wild-type NUT may be recruited to p300-enriched sites, given that it binds p300, in which case it is predicted to colocalize with CIC-NUTM1, where it may function similarly to the NUT fusion oncoprotein to stimulate p300 HAT activity. Whether or not wild-type NUT may enhance an already hyper-activated p300 is not known, but likely it would be an incremental, if not minimal effect because p300 HAT activity is likely already saturated (please refer to the p300-activating properties of NUT in Reynoird et al. EMBO J. 2010 Sep 1;29(17):2943-52). We believe that the expression of NUT in *CIC*-rearranged cells is related mostly to cell of origin. While it is not known what upregulates NUT expression, its upregulation in subsets of *CIC*-rearranged tumors may be specified by their enhancer landscapes. Based on the significance of wild-type NUT expression not being known in *CIC*-rearranged sarcoma, we have revised Figure 1C and cropped out the wild-type bands for NUT and DUX4:

Revised Figure 2C (*formerly 1C*):

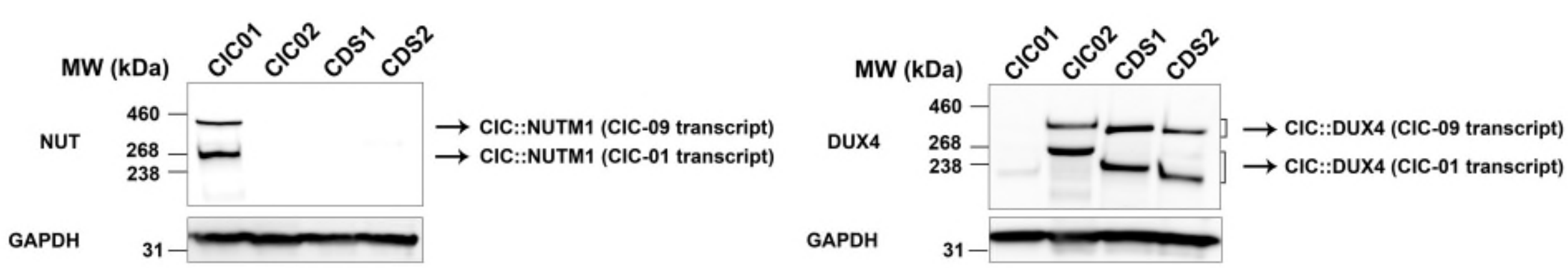

Fig. 2C: Mass spectrometry studies in CIC01 and CIC02 demonstrate the presence of CIC::NUT and CIC::DUX4 fusion oncoproteins, respectively, as visualized by immunoblotting studies. Fusion proteins appear as two bands resulting from a longer CIC-09 and shorter CIC-01 transcript.

5) Line 145: Fig.1D- Although high MYC expression is often associated with RB loss or inactivation, RB protein can sometimes remain detectable due to hyperphosphorylation. Given the relatively high RB levels observed in some MYC-high samples, could the authors please clarify whether this reflects phosphorylated RB or another mechanism of RB stabilization?

- We thank the reviewer for this insightful question. *CIC*-rearranged sarcomas typically do not exhibit Rb loss, which is consistent with our ability to detect total Rb protein in these

samples. To further clarify the reviewer's point regarding Rb phosphorylation status, we have now included immunoblot data for phospho-Rb (Ser795) in Figure 2D (*formerly 1D*). This addition helps distinguish between total and phosphorylated Rb and provides further context for the observed Rb levels in MYC-high samples.

Revised Figure 2D (*formerly 1D*):

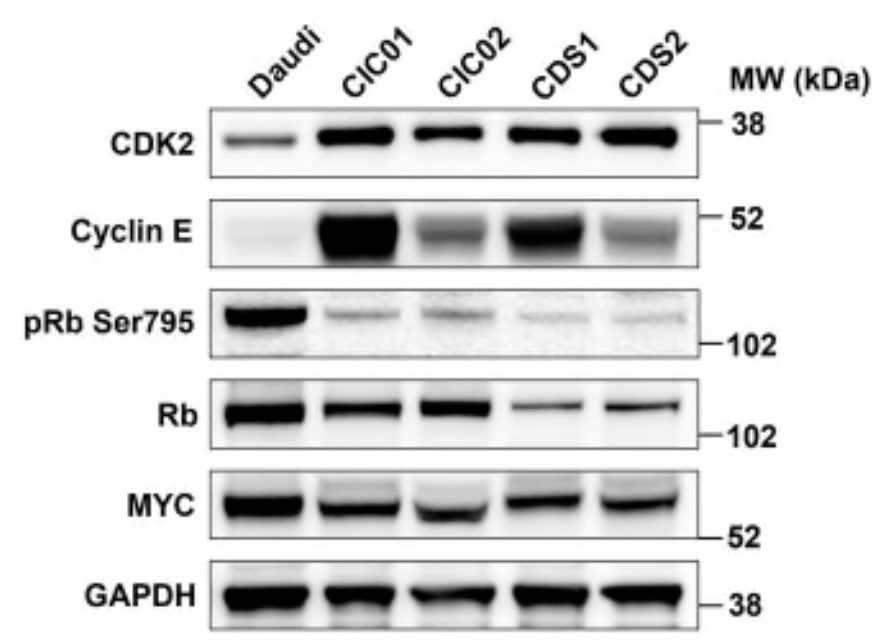

Fig. 2D: Immunoblotting studies demonstrate that CIC01, CIC02, CDS1, and CDS2 expressed CDK2 and overexpressed cyclin E, while pRb Ser795 and Rb were retained. All four cell lines show strong MYC expression, comparable to levels detected in Daudi cells. GAPDH serves as loading control.

- We've revised the Result section (lines: 175-176) accordingly: "Furthermore, pRb Ser795 and Rb were retained, which was predictive of potential sensitivity to CDK2-targeted therapies (**Fig. 2D**).[20]"

6) There are several issues with the dose response curves that need to be fixed.
a. For all of the compound dosing data presented the IC50 is presented as a single number, but this does not capture the variability present in data with technical replicates or the uncertainty that comes from performing a nonlinear regression to fit a curve. I went to the tool listed in the methods section and entered some data from a dose-response curve in our lab. It is clearly fitting to the curve to the right equation, and somewhat obliquely reports the maximum, minimum, Hill slope, and IC50, but it is not reporting any measures that allow you to capture the uncertainty inherent in your measurement. IC50s should have 95% confidence intervals reported with them, and you should evaluate the actual curve fit by looking at QC measures such as the R^2 value. These are readily available when analyzing this type of data in GraphPad, which appears to be the software used to generate plots. The 95% CIs should be included in Supplementary Table S1.

- We are grateful for the reviewer's suggestion to use the GraphPad Prism software, which we used to generate graphs, to determine IC_{50} values and corresponding 95% confidence intervals (CI). We have updated the IC_{50} values and added the CIs in Supplementary Table S1:

Revised Supplementary Table S1:

Proliferation IC_{50} values of CDK2 and CDK9 inhibitor drugs in *CIC*-rearranged sarcoma cell lines.

	IC_{50} value [nM] (95% CI)					
**Cell line**	**Tagtociclib**	**KB-0742**	**AZD4573**	**BLU-222**	**PF-06873600**	**Palbociclib**
CIC01	71.32 (52.17-97.74)	589.6 (325.4-760.3)	20.49 (14.56-28.56)	12.88 (9.103-18.09)	39.12 (30.02-50.99)	Not reached
CIC02	45.6 (35.50-58.33)	744.4 (499.7-1089)	10.56 (8.110-13.65)	33.74 (23.29-48.98)	34.39 (26.23-45.03)	Not reached
CDS1	125.4 (89.91-176.8)	524.6 (238.3-935.9)	1.16 (0.8552-1.539)	15.86 (11.97-20.95)	3.505 (1.101-10.68)	Not reached
CDS2	23.91 (10.20-54.42)	304.1 (197.3-349.5)	5.406 (3.813-7.555)	369.3 (177.4-896.6)	12.57 (8.960-17.48)	Not reached
Daudi	824.7 (402.0-2444)	1066 (548.3-1392)	18.65 (13.60-25.53)	-	-	-
U2-OS	-	1187 (919.0-1494)	38.93 (31.26-48.4)	-	-	-

We updated the IC_{50} values in the Results (lines: 187-190): Treatment of the *CIC*-rearranged cell lines with tagtociclib substantially inhibited cell proliferation (**Fig. 3A**) with IC_{50} values ranging from 23.91-125.4 nM (**Supplementary Table S1**), whereas cell viability was reduced to a lesser extent (**Fig. 3B**).

And (lines: 267-273): Our cell-based assays demonstrated that KB-0742 inhibited cell proliferation and cell viability and increased apoptosis in CIC01, CIC02, CDS1, and CDS2 (**Fig. 5A-C**), with proliferation IC_{50} values ranging from 304.1-744.4 nM (**Supplementary Table S1**). Similarly, treatment with AZD4573 inhibited cell proliferation and cell viability and increased apoptosis in CIC01, CIC02, CDS1, and CDS2 (**Fig. 5D-F**), with proliferation IC_{50} values ranging from 1.16-20.49 nM (**Supplementary Table S1**). U2-OS cells were substantially less sensitive to either treatment (**Fig. 5A-F**).

We also updated the Methods (line: 593): IC_{50} values were calculated using GraphPad Prism.

b. Given that the plots are made in GraphPad, it is unclear if the curves presented are also from a nonlinear regression and if the values there match the values presented in the text and Supplementary Table S1. This is particularly important, because some of the curves presented (Panels 2I, 4A-B, S5A-B) appear to show a curve that has not turned over to a minimum value yet, which would indicate that those data are fit to a model with negative cell viability, which is physically impossible. When fitting in GraphPad, you are able to constrain your curve fit with a minimum value that remains at or above 0.

- We appreciate the reviewer's suggestion and have constrained each of the nonlinear models to have a bottom threshold >0 and have updated all the corresponding graphs (Figures 3I, 5A-B, and Supplementary Fig. S5A-B) including the IC_{50} values and confidence intervals (see revised Supplementary Table S1, above).

New Figure 3I (*formerly 2I*):

Revised Figure 5A-B (*formerly 4A-B*):

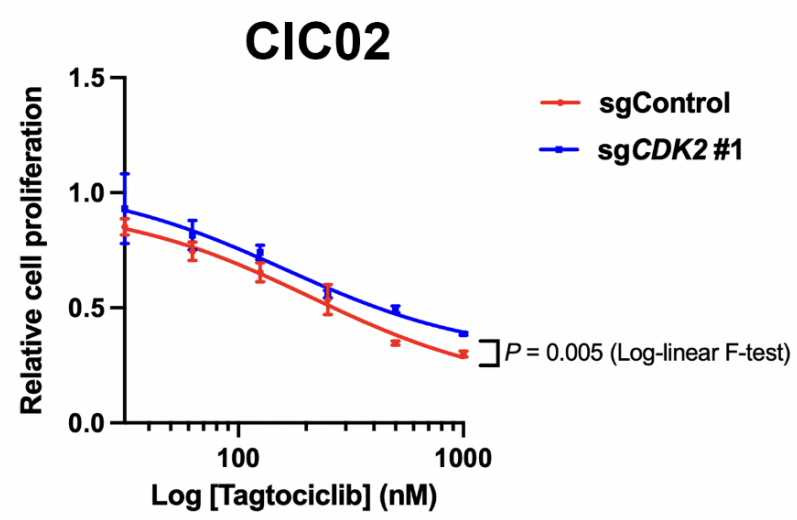

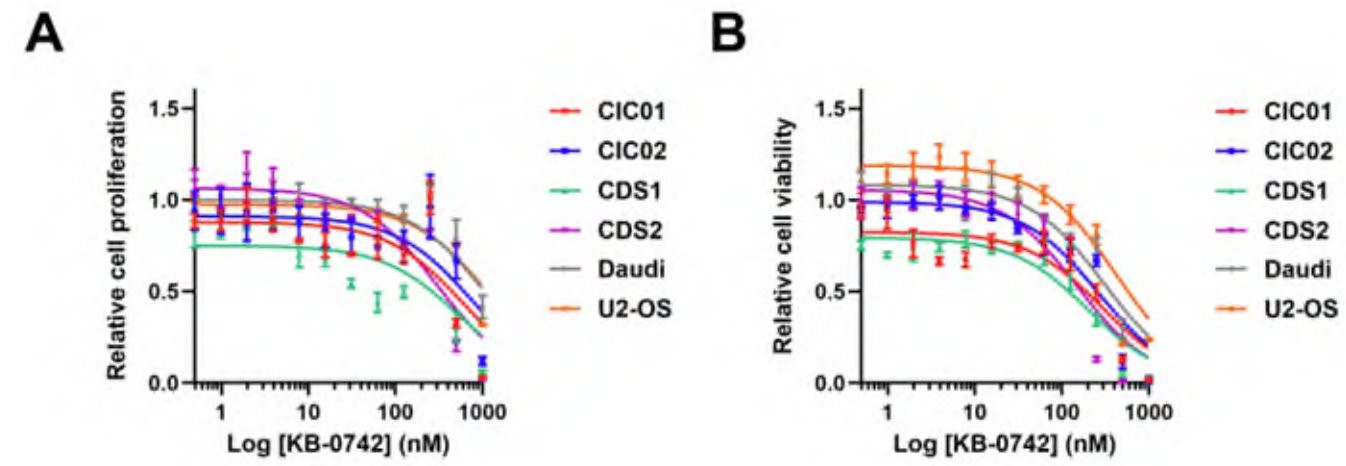

Revised Suppl. Fig. S5A-B:

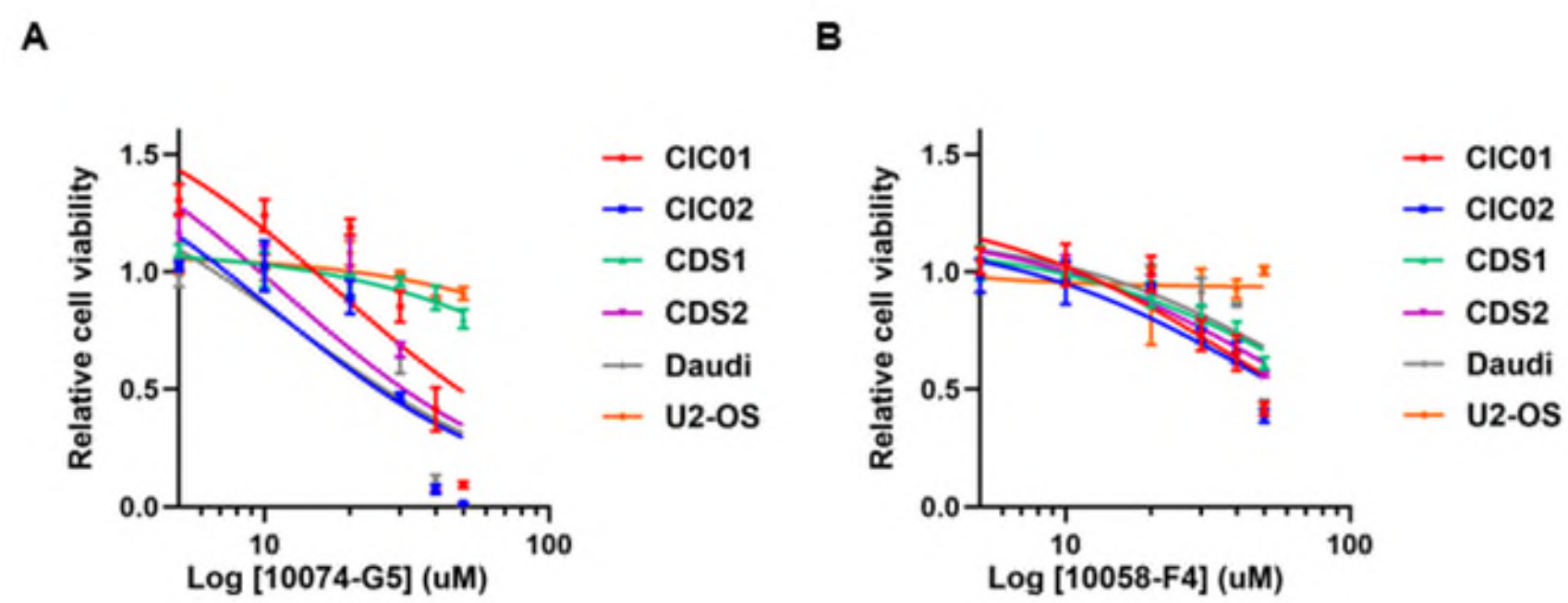

c. In panel 2I, this is not the typical way to report resistance. You should evaluate an IC50 for each condition and compare them and whether their 95% CI overlap. Here, there is not quite enough data to be confident in your curve fits of an IC50 for either condition. Somewhat related, do the authors have any idea about the sgControl looks to have a higher IC50 that the parental cell line along in panel 2A?

- We revised Figure 3I (formerly 2I) to show the effect of tagtociclib on cell proliferation 12 days after *CDK2* knockout in the CIC02 cell line. We acknowledge that the data points are too few to have a stable estimate for the variability of the IC_{50} values (161.1 versus 210 nM) which makes the 95% confidence intervals very wide (69.01 to 369.9 and 143.4 to 308.0, respectively). However, since we had fewer cells available for the comparison of response to tagtociclib +/- *CDK2* knockout, we were able to evaluate fewer doses (i.e., 0, 31.25, 62.5, 125, 250, 500, and 1000 nM). We therefore decided to use a log-linear F-test to statistically evaluate the difference between the two curves and added this to the Results (lines: 218-221): *CDK2* knockout significantly reduced the sensitivity of the CIC02 cell line to tagtociclib as assessed by BrdU incorporation 12 days post infection when using an F-test applied to the corresponding log-linear models, thereby confirming the relative target selectivity of tagtociclib for CDK2 (**Fig. 3I**).

  The small number of observations may also explain the apparent difference in IC_{50} values for tagtociclib in the sgControl (Figure 3I) (*formerly 2I*) and the parental CIC02 cell line (Figure 5A) (formerly 4A).

Revised Figure 3I (*formerly 2I*):

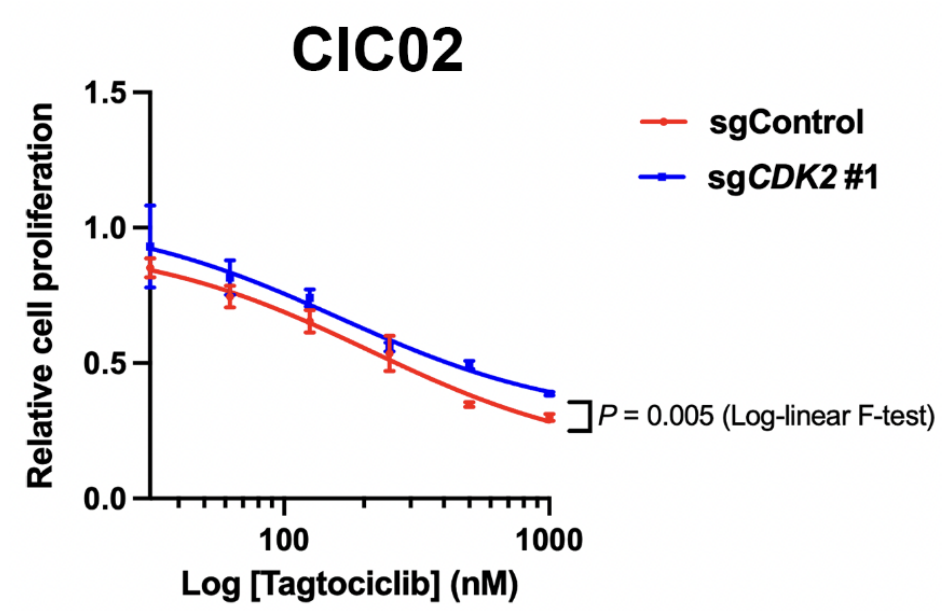

Fig. 3I: *CDK2* knockout significantly reduced the sensitivity to tagtociclib as assessed by BrdU incorporation at 24 h in the CIC02 cell line 12 days post-infection (normalized to DMSO).

7) Can the data from 2G be quantified with image analysis?

- We appreciate the reviewer's suggestion and have quantified the cells using the Cellpose-SAM tool. We have revised Figure 3G (*formerly 2G*) to include bar graphs for each cell line:

Revised Figure 3G (*formerly 2G*):

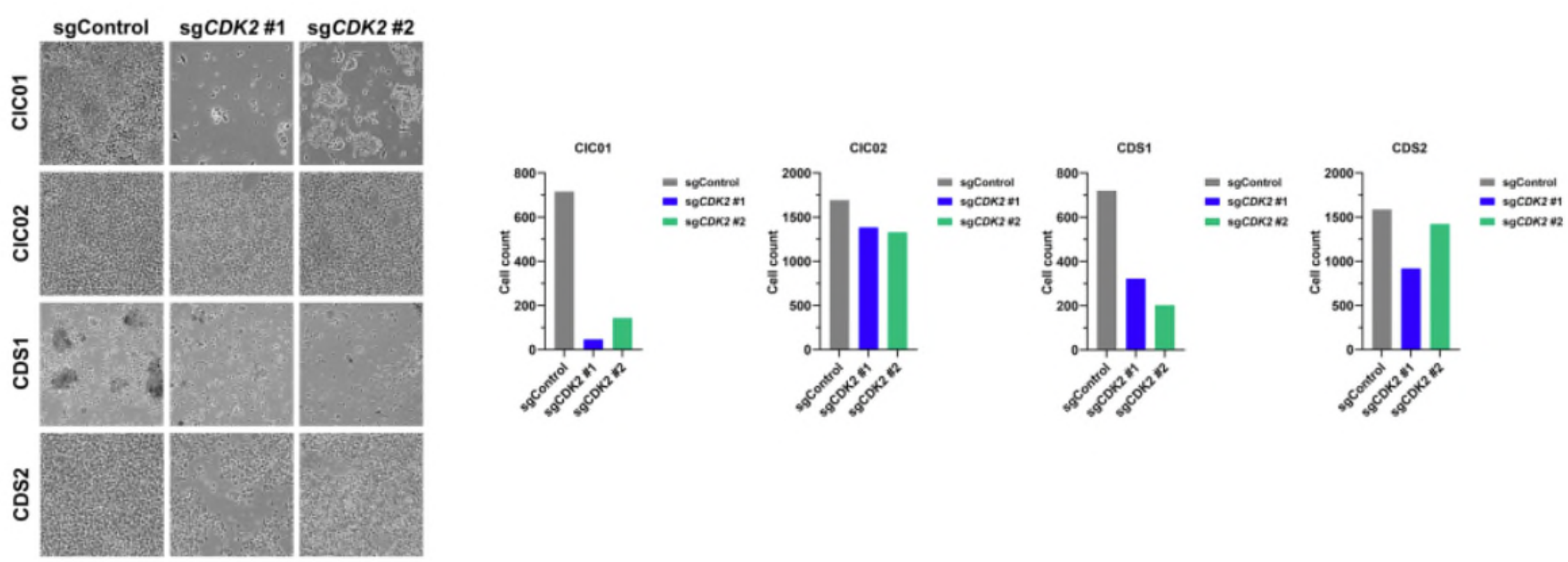

Fig. 3G: Both sgRNAs directed against CDK2 also reduced cell counts, as shown in phase-contrast photographs 14 days post-infection. The empty vector served as a control. Bar graphs illustrate corresponding cell counts obtained with semiautomated quantification using the Cellpose-SAM tool.

We also added a description to the Methods (lines: 575-590):
Cell segmentation was performed using the Cellpose framework (v4.0.8; Python 3.10.19)[59], incorporating the Segment Anything Model (SAM) via the Cellpose-SAM interface to improve boundary detection.[60] For standard samples, pre-trained cyto3[61] and base models were initially applied. To address to differences in cell morphology and confluence across samples, a custom segmentation model (CDS3) was developed using an iterative human-in-the-loop training approach.[62] Representative images were divided into non-overlapping 256 × 256-pixel tiles to increase feature density and improve local boundary detection. The CDS3 model was initialized with cyto3 weights and fine-tuned for 100 epochs in a GPU-accelerated training environment to capture the characteristic cell shapes and boundary features present in the dataset. To ensure high biological accuracy, all automated segmentation outputs underwent comprehensive manual curation. Using the Cellpose graphical user interface, each segmentation mask was visually inspected and manually corrected, redrawn, or removed where necessary. While the automated models, including CDS3, provided efficient initialization, the final datasets used for downstream analyses consisted exclusively of manually verified masks.

And added 4 new references:

59. Stringer C, Wang T, Michaelos M, Pachitariu M. Cellpose: a generalist algorithm for cellular segmentation. Nat Methods 18, 100-106 (2021).

60. Pachitariu M, Rariden M, Stringer C. Cellpose-SAM: superhuman generalization for cellular segmentation. bioRxiv, 2025.2004.2028.651001 (2025).

61. Stringer C, Pachitariu M. Cellpose3: one-click image restoration for improved cellular segmentation. Nat Methods 22, 592-599 (2025).

62. Pachitariu M, Stringer C. Cellpose 2.0: how to train your own model. Nat Methods 19, 1634-1641 (2022).

8) In discussing the data presented in Panel 2H, CDK2 is hardly knocked down. The authors stated that cells die at later timepoints (14 d) making it hard to assess CDK2 levels, but is there any data to show CDK2 depletion at intermediate timepoints, particularly for sgCDK2 #2? Also, the data in 2G indicate that there are cells in CIC02 and CDS2 that could be assayed at 14 days.

- We thank the reviewer for this insightful comment. To address this concern, we repeated the *CDK2* knockout experiments in all four *CIC*-rearranged sarcoma cell lines and extended the analysis to a later time point. Consistent with the reviewer's suggestion, immunoblot analyses demonstrated a substantially more pronounced reduction in CDK2 expression, accompanied by decreased MYC levels, at 7 days post-infection compared with 3 days post-infection. Importantly, at this later time point, sg*CDK2* #2 also produced a clear reduction in CDK2 protein levels, supporting its on-target activity and confirming more effective target depletion than was apparent at the earlier time point. These additional data have been incorporated into the revised Figure 3H (formerly Figure 2H).

  Regarding the reviewer's observation that a subset of cells remained viable in CIC02 and CDS2 at later time points, we agree that residual cells could still be detected. However, the substantial loss of cell viability and reduced cell numbers at these later stages limited our ability to reliably assess protein expression. We therefore focused our analysis on the 7-day time point, which allowed us to more clearly demonstrate CDK2 depletion while minimizing potential confounding effects associated with extensive cell death.

Revised Figure 3H:

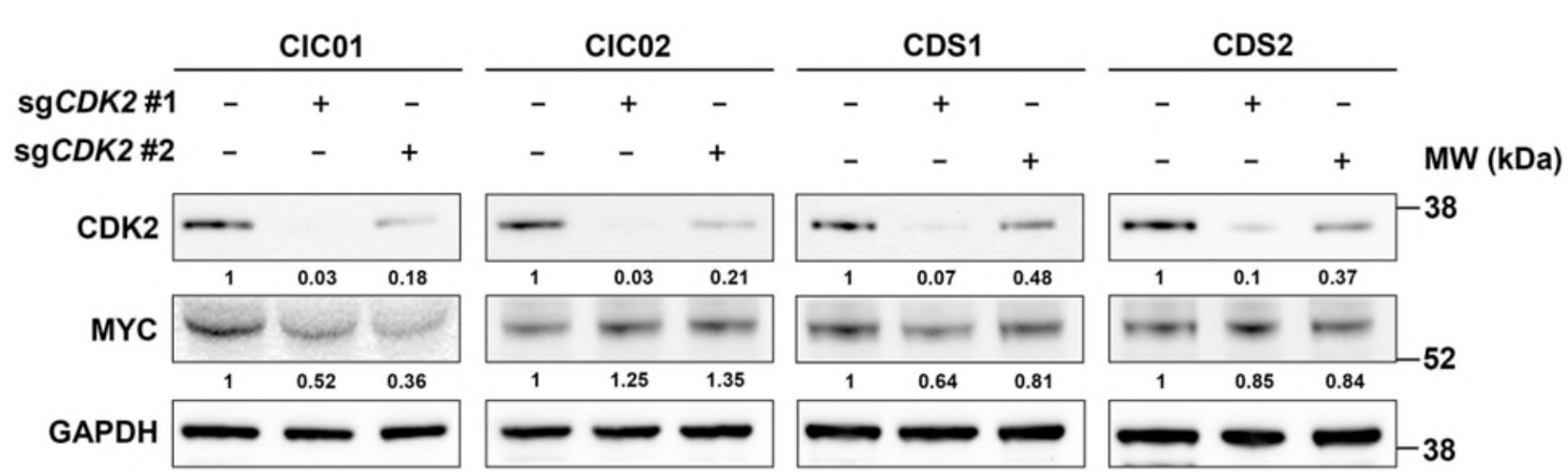

Fig. 3H: *CDK2* knockout not only reduced CDK2 expression but also MYC expression 7 days post-infection.

We revised the Results section accordingly to reflect the later time point and included an additional reference to emphasize a the role of CDK2 for regulating MYC stability (lines: 215-218): Interestingly, *CDK2* knockout not only reduced CDK2 expression but also MYC expression in three cell lines 7 days post infection (**Fig. 3H**). Interestingly, *CDK2* knockout not only reduced CDK2 expression but also MYC expression in some cell lines 7 days post infection (**Fig. 3H**), possibly due to reduced CDK2-mediated phosphorylation of MYC at Ser62, which affects MYC stability and senescence.[25, 26] ~~At this early time point, prior to the start of selection for puromycin resistance, residual CDK2 expression was believed to stem from a subset of uninfected cells, with possible compensatory CDK2 upregulation; capturing CDK2 expression at a later time point post infection was impaired by the inability to prepare cell lysates due to dramatic effects on cell density and viability, further supporting oncogenic dependence on CDK2 in these models (**Fig. 3G**).~~

26. Wang D, et al. Phosphorylation of c-Myc at Ser62 via the protein kinase activity of acetaldehyde dehydrogenase 18A1 promotes tumor growth. Mol Ther 33, 5860-5882 (2025).

9) The data presented in 2K should include a more robust analysis of quantified, or semi-quantification of multiple high-magnification fields for each condition, or at least some additional replicates if possible. A single image of a slide is not sufficient.

- We thank the reviewer for this helpful suggestion regarding the robustness of the analysis presented in Figure 3K (*formerly 2K*). In response, we have revised Figure 3K to strengthen the rigor and representation of the data. Specifically, we now include histologic images from two mice per condition, with two distinct regions analyzed per mouse. This provides additional biological and regional replication beyond the single representative image previously shown. We agree that a single image is insufficient to capture the variability within each condition, and we believe these additions substantially improve the reliability and interpretability of the data. The figure legend has been updated accordingly to clarify the expanded sampling.

Revised Figure 3K (*formerly 2K*):

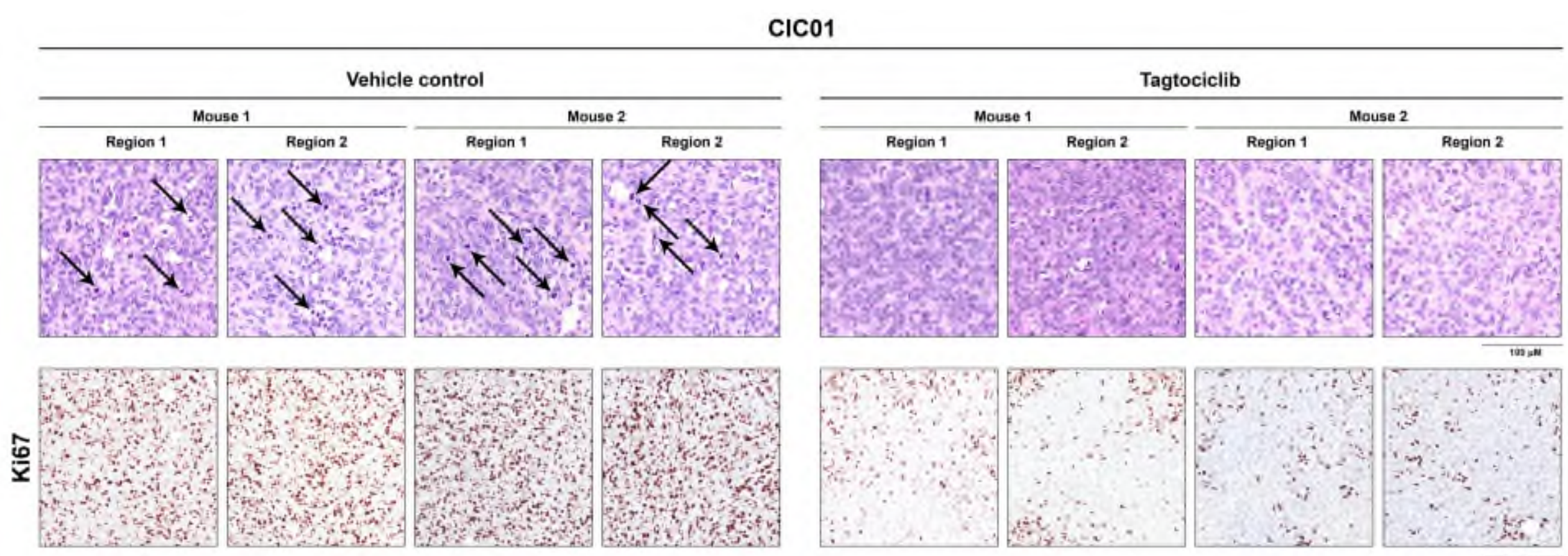

Fig. 3K: Tagtociclib treatment at 150 mg/kg BID for 28 days also eliminated mitotic activity and Ki67 proliferation index in CIC01 PDX cells compared to the vehicle control (shown are N=2 per arm with two regions sampled). Mitoses are indicated by arrows.

10) The transcriptomic analysis has several issues as outlined below:

a. For panel 3A, these data need to be presented with the hierarchical clustering tree shown to properly evaluate how samples are clustering. Simply being next to each other vertically is not sufficient to say they cluster without showing the branching. This is particularly important for the left subpanel of panel 2A.

- We thank the reviewer for this valuable suggestion and agree that displaying the hierarchical clustering structure improves the interpretation of sample relationships within the dataset. In response, we now include the corresponding hierarchical clustering dendrograms for each analysis. The dendrograms have been added directly beneath the heatmaps, allowing readers to visualize the branching structure and clustering distances that underlie sample grouping. We believe that these revised figures provide a more rigorous and transparent representation of the clustering results, particularly for the left subpanel of Figure 3A, and facilitate assessment of the similarity between samples.

Revised Figure 4A (*formerly 3A*):

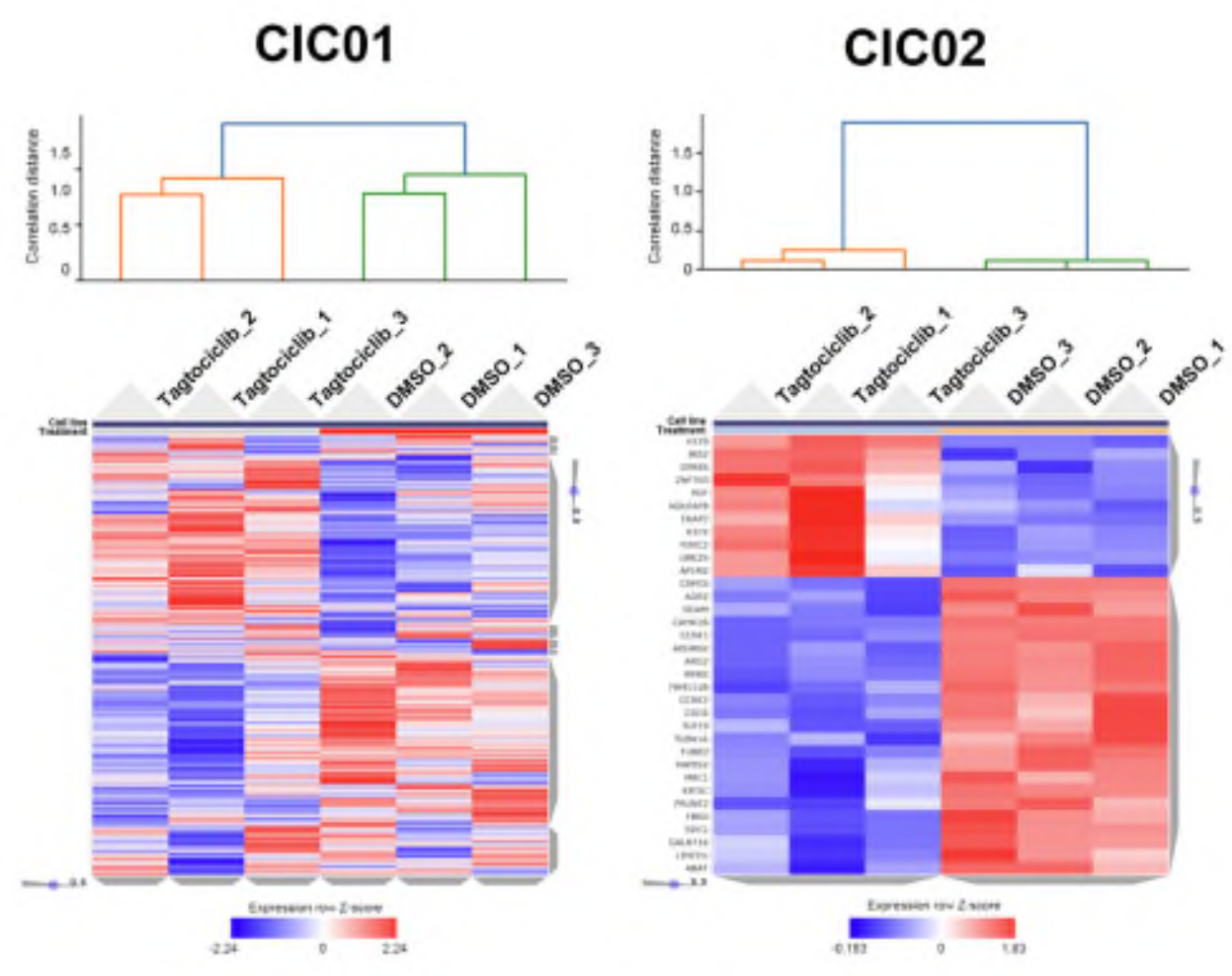

b. There is no way to gauge differential gene expression without the inclusion of a legend showing what the colors represent for these heatmaps.

- We thank the reviewer for this helpful suggestion. We agree that the absence of a color scale limited the interpretability of the heatmaps. To address this, we have added a color legend indicating the expression row z-scores to Figure 4A. This revision enables readers to readily interpret the magnitude and direction of relative gene expression changes represented by the heatmap colors:

Revised Figure 4A (*formerly 3A*):

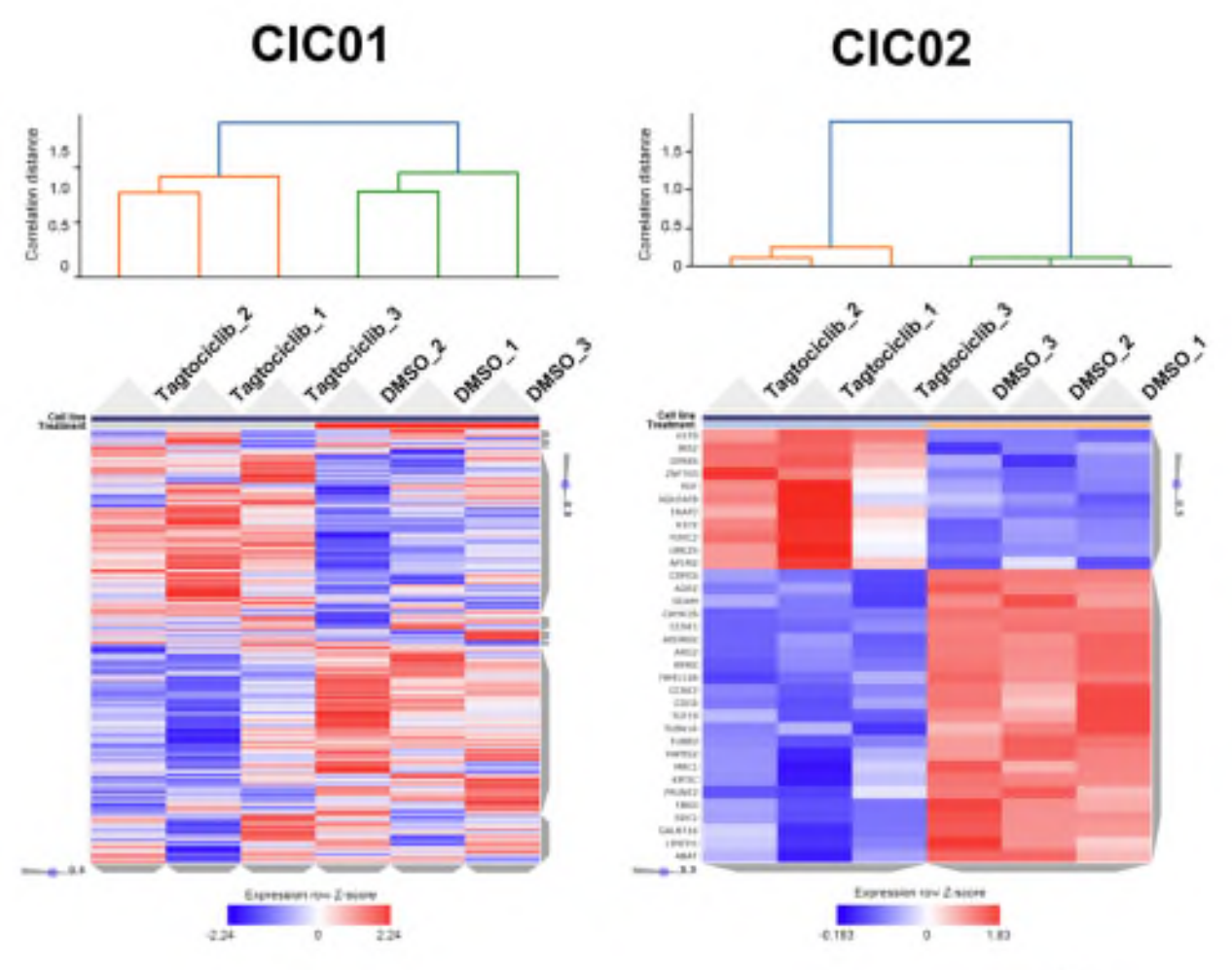

c. The data from Enrichr in panel 3B need to presented with some sort of scale with each sub panel. I see that there are data presented in Supplementary Table S2, however, when I plug in the same genes to Enrichr I get qualitatively the same results, but with slightly different values. Can you discuss which program version you performed these analyses with to improve transparency and reproducibility in reporting bioinformatics analyses?

- We thank the reviewer for this helpful comment regarding the presentation and reproducibility of the enrichment analyses. Enrichr is a continuously maintained and updated web-based platform, and as a result, enrichment statistics may vary slightly over time as underlying gene set libraries are revised and expanded. Nevertheless, the overall enrichment patterns and biological conclusions remain highly reproducible. To improve transparency and reproducibility, we have provided direct links to the Enrichr analyses used in this study in the Methods section, allowing readers to access the analyzed datasets and results.

  The corresponding enrichment statistics and P values for the pathways shown in Figure 4C are shown in Supplementary Table S2 and we verified that the results remained identical when analyzing the data using the updated Enrichr output. The bars in Figure 4C are sorted by *P* value ranking as specified in the figure legend. Gray indicates a non-significant *P* value, whereas shades of red indicate a significant *P* value; bright red indicates the lowest P value. We have added this information to the figure legend:

  Figure 4C: Analysis of the transcription factor protein-protein interactions (PPI) library demonstrates that tagtociclib treatment results in downregulation of the MYC transcription factor signature in CIC01 and CIC02 indicating inhibition of genes encoding proteins that interact with MYC. Signatures are ranked by *P* value. Red bars indicate $P \leq 0.05$; gray bars indicate $P > 0.05$.

  We also confirmed that the graphs shown in Figure 4A, C-E using the updated Enrichr output remained the same with the exception of the networks shown in Figure 4E which are displayed at a slightly rotated angle and were therefore updated in Figure 4E:

Revised Figure 4E (*formerly 3D*):

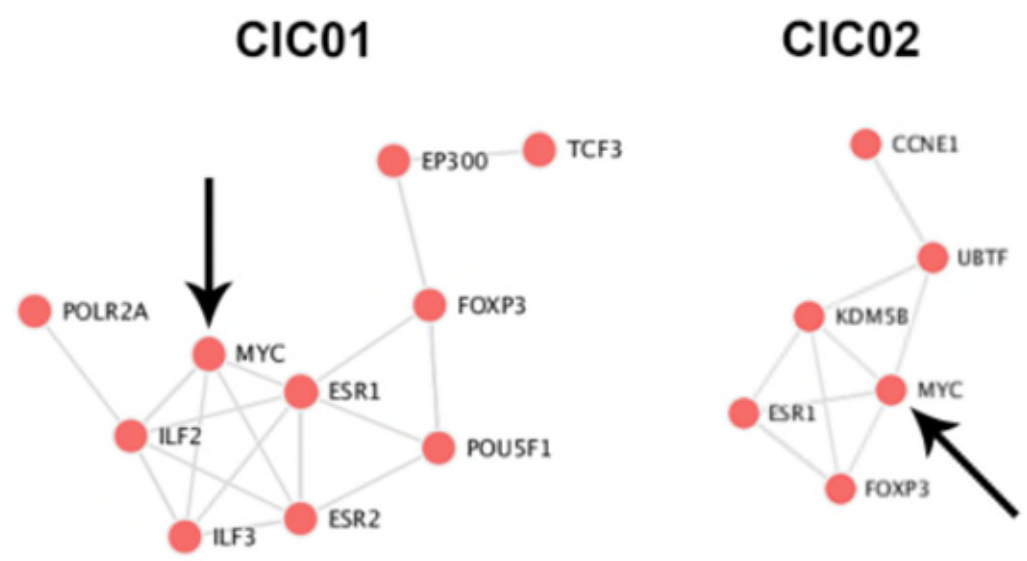

We hope these revisions improve the clarity, transparency, and reproducibility of the bioinformatic analyses presented in the manuscript.

d. Likewise, a more complete description of the data would report the differentially expressed genes (both up and down) in a separate supplementary table, and discuss how gene lists were curated for analysis with Enrichr. For example, for the CIC02 cell line, I see that 4 genes were the input for Enrichr, but there were 11 differentially expressed downregulated genes detected in the differential expression analysis. Why were only 4 genes chosen? They are not the 4 common between both cell lines. Likewise, out of 965 downregulated genes for CIC01, why are only 10 used for Enrichr input? Pathway analysis should be performed using the whole set of differentially expressed genes, unless there is a cutoff being used that is not reported. If there is a separate cutoff performed from what is reported on Line 564.

- We thank the reviewer for this comment and appreciate the opportunity to clarify our analysis workflow. The Enrichr analyses were performed using the complete sets of significantly upregulated and downregulated genes identified in each cell line based on the differential expression criteria described in the Methods. No additional filtering or gene selection was applied prior to pathway enrichment analysis.

  The apparent discrepancy arises from a misunderstanding of the Venn diagrams shown in the figure. The numbers displayed in the Venn diagrams (e.g., 4 commonly downregulated genes and 11 commonly upregulated genes) represent only the overlap of differentially expressed genes between the two cell lines and do not reflect the number of genes submitted to Enrichr. For example, all significantly differentially expressed genes identified in CIC02 and CIC01 were included in the enrichment analyses, whereas the Venn diagrams were intended solely to illustrate the subset of genes shared between the two datasets.

  To avoid confusion, we have revised the Supplementary Fig. S4 figure legend and corresponding text in the Results section (lines: 239-243) to more clearly distinguish between the genes used for enrichment analysis and the overlapping gene sets displayed in the Venn diagrams:

  Supplementary Figure S4. Common significantly down- and upregulated genes in CIC01 and CIC02.
  Venn diagram showing significantly down- and upregulated genes common to both cell lines as assessed by RNA sequencing analysis after 48 hours of treatment with tagtociclib.

  Results: All significantly differentially expressed genes identified in CIC02 and CIC01 were included in the enrichment analyses. *CCNE1* and *CCNE2* were among the 11 most significantly upregulated genes common in CIC01 and CIC02 cells in response to tagtociclib treatment (**Supplementary Fig. S4**), indicating compensatory cyclin E upregulation upon CDK2 inhibition.

e. Related to comment 5d, why did authors choose to present the Transcription Factor PPI library from Enrichr from all of the possible enrichment options? I believe this means that enriched transcription factors are likely interactors with the genes listed in the gene set, as opposed to transcription factors that are regulated by the differentially expressed genes or transcription factors that control expression of the differentially expressed genes, either of which may be more interesting.

- We thank the reviewer for this important comment and agree that the interpretation of enrichment analyses depends on the specific gene set library being queried. As noted by the reviewer, the Enrichr Transcription Factor PPI library consists of genes whose protein products are known to interact with a given transcription factor, rather than genes directly regulated by that transcription factor or transcription factors whose expression is altered in the dataset.

  We chose to highlight the Transcription Factor PPI library because it yielded one of the strongest and most biologically relevant enrichment signals in our analysis, with MYC-associated interacting proteins emerging prominently among genes affected by CDK2 inhibition. Given the established functional relationship between CDK2 and MYC in several cancer contexts, we considered this finding particularly noteworthy. However, we agree that the enrichment should not be interpreted as evidence that CDK2 inhibition directly regulates MYC transcription or MYC target gene expression.

  To avoid this potential misunderstanding, we have revised the Results section to clarify that CDK2 inhibition is associated with altered expression of genes encoding proteins that interact with MYC, rather than changes in MYC transcript levels or direct regulation of canonical MYC target genes. We have also revised the corresponding figure legend and text to more accurately reflect the nature of the enrichment analysis and its biological interpretation (see also our response to the following comment).

  Results (line: 246-250): However, analysis of the transcription factor protein-protein interactions (PPI) library uncovered that tagtociclib treatment of CIC01 and CIC02 cells resulted in downregulation of genes encoding proteins that interact with MYC, indicating an indirect effect of tagtociclib treatment on MYC signatures (**Fig. 4C-E, Supplementary Table S2**).

  Fig. 4C: Analysis of the transcription factor protein-protein interactions (PPI) library demonstrates that tagtociclib treatment results in downregulation of the MYC transcription factor signature in CIC01 and CIC02 indicating inhibition of genes encoding proteins that interact with MYC. Signatures are ranked by *P* value. Red bars indicate $P \leq 0.05$; gray bars indicate $P > 0.05$.

f. Given all of the issues with the transcriptomic analysis discussed, the subsection title on line 209 and the figure legend title for figure 3 both indicating that tagtociclib inhibits MYC transcript expression and MYC oncogenic programs are highly misleading. That is not what this transcriptomic analysis shows. The data presented here indicate that the differentially expressed genes are known to interact with MYC protein, not that the compound is having any effect on MYC transcript level. The data needed to support these titles would include an analysis of MYC mRNA expression or GSEAs looking at MYC target gene expression in the context of CDK2 inhibition. Alternatively, the titles of these sections should be changed to more accurately reflect the transcriptomic data presented.

- We appreciate the reviewer's suggestion. See also our response to Reviewer 1, Point #2:

  To further address whether MYC regulation occurs at the transcriptional or post-translational level, we performed additional mechanistic studies. As shown in newly added Figure 4B,

tagtociclib treatment did not suppress MYC transcription, suggesting that the observed reduction in MYC expression is not primarily mediated at the transcriptional level. We next examined the effects of tagtociclib in combination with cycloheximide, an inhibitor of protein translation. As shown in Figure 4F, combined treatment with tagtociclib and cycloheximide produced an additive reduction in MYC protein expression, supporting a model in which tagtociclib primarily inhibits MYC stability rather than inhibiting MYC translation.

Together, these findings provide additional mechanistic insight into MYC regulation in *CIC*-rearranged sarcoma cells following CDK2 inhibition. We have also incorporated these results into the newly added schematic model presented in Figure 6J (see also our response to Minor Comment #1).

Revised Figure 4 (*formerly 3*):

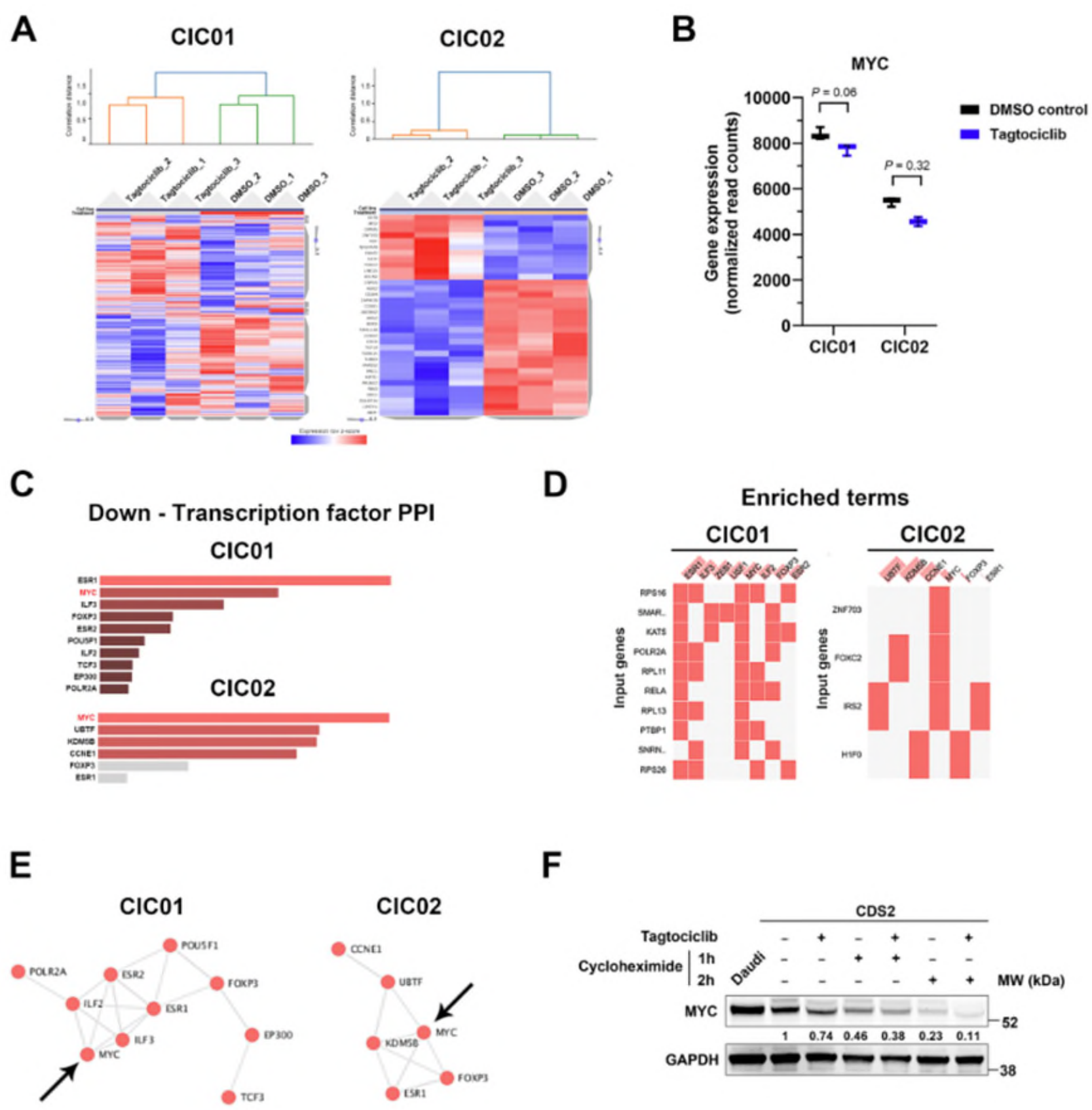

Fig. 4. Tagtociclib inhibits MYC oncogenic programs and reduces MYC protein stability.
(A) RNA sequencing analysis was performed in triplicate in the CIC01 and CIC02 cell lines after treatment with tagtociclib at 200 nM for 48 hours. Unsupervised hierarchical clustering demonstrates that tagtociclib treated cells separated from DMSO-control within each cell line. **(B)** Tagtociclib treatment did not suppress MYC transcript expression in CIC01 and CIC02. **(C)** Analysis of the transcription factor protein-protein interactions (PPI) library demonstrates that tagtociclib treatment results in downregulation of the MYC transcription factor signature in CIC01 and CIC02 indicating inhibition of genes encoding proteins that interact with MYC. Signatures are ranked by *P* value. Red bars indicate $P \leq 0.05$; gray bars indicate $P > 0.05$. **(D)** Heatmap showing the genes downregulated by tagtociclib treatment in CIC01 and CIC02 that contribute to the MYC transcription factor signature. **(E)** Functional networks for the MYC transcription factor signature highlighting MYC and other genes significantly downregulated by tagtociclib treatment in CIC01 and CIC02. **(F)** Combined treatment with tagtociclib at 1 μM for 6 days and cycloheximide at 178 μM for 1 or 2 hours produced an additive reduction in MYC protein expression, supporting a model in which tagtociclib primarily reduces MYC stability rather than inhibiting MYC translation.

We revised the heading of the Results section accordingly and included our new observations (lines: 235-256):

Tagtociclib inhibits MYC oncogenic programs and reduces MYC protein stability.
To determine the effects of CDK2 inhibition at the transcriptomic level, we performed RNA sequencing analysis of the CIC01 and CIC02 cell lines in response to tagtociclib. Unsupervised hierarchical clustering demonstrated that cells treated with tagtociclib were separated from DMSO-control within each cell line (**Fig. 4A**). All significantly differentially expressed genes identified in CIC02 and CIC01 were included in the enrichment analyses. *CCNE1* and *CCNE2* were among the 11 most significantly upregulated genes common in CIC01 and CIC02 cells in response to tagtociclib treatment (**Supplementary Fig. S4**), indicating compensatory cyclin E upregulation upon CDK2 inhibition. Tagtociclib treatment did not suppress MYC transcription (**Fig. 4B**) suggesting that the observed reduction in MYC expression and downregulation is not primarily mediated at the MYC transcriptional level. However, analysis of the transcription factor protein-protein interactions (PPI) library uncovered that tagtociclib treatment of CIC01 and CIC02 cells resulted in downregulation of genes encoding proteins that interact with MYC, indicating an indirect effect of tagtociclib treatment on MYC signatures (**Fig. 4C-E, Supplementary Table S2**). We next examined the effects of tagtociclib on MYC protein stability using cycloheximide, a strong inhibitor of protein translation, to assess protein turnover. Combined treatment with tagtociclib and cycloheximide produced an additive reduction in MYC protein expression (**Fig. 4F**), supporting a model in which tagtociclib primarily decreases MYC stability rather than inhibiting MYC transcription. Together, these findings suggest that CDK2 inhibition in *CIC*-rearranged sarcoma cells downregulates MYC transcriptional activity by reducing MYC protein stability.

We added the following sections to the Discussion (lines: 343-345 and 402-409): Interestingly, our studies also demonstrated that CDK2 inhibition and *CDK2* knockout resulted in downregulation of MYC expression through decreased MYC protein stability rather than inhibition of MYC transcription (**Fig. 6J**). [...] In summary, our study highlights *CIC*-rearranged sarcoma as a MYC-dependent cancer and demonstrates anti-tumor effects of concurrent inhibition of CDK2 and CDK9 through proliferative arrest and induction of caspase 3-mediated apoptotic cell death in preclinical models of *CIC*-rearranged sarcoma. We further identify distinct but complementary mechanisms of MYC regulation, whereby CDK9 inhibition suppresses MYC transcription and CDK2 inhibition impairs MYC protein stability. Our findings provide key preclinical data that could serve as a rationale for clinical trials evaluating CDK2 and CDK9-targeted therapies in patients with *CIC*-rearranged sarcoma.

11) Line 219-224 (Results) – It was unclear how this information was connected to the results shown for figure 3 or any other relevant discussion of MYC. Is this information perhaps more suited to the Discussion section?

We appreciate the reviewer's suggestion and have removed that paragraph about CDK9 to the Discussion where it is more appropriate (lines: 356-367): CDK9, a Ser/Thr protein kinase and catalytic subunit of positive transcription elongation factor b (P-TEFb), regulates transcriptional elongation by phosphorylating RNA polymerase II at serine 2 (pRPB1-CTD Ser2). Transient inhibition of CDK9 modulates the expression of genes with short-lived transcripts and labile proteins, providing a potential therapeutic opportunity in cancers dependent upon oncogenes fitting these criteria.[38] KB-0742[27, 39, 40] and AZD4573[28, 38] are potent and selective ATP-competitive CDK9 inhibitors that inhibit phospho-RPB1-CTD Ser2, thereby decreasing the expression of MCL-1, a member of the BCL-2 family of proteins that prevents apoptosis, and the

oncogene MYC, which induces apoptosis in a broad range of malignancies. KB-0742 and AZD4573 are being evaluated clinically for MYC-dependent hematologic malignancies (NCT04718675, NCT05140382, NCT04630756, and NCT03263637).

12) Line 259: (Results) - Is MYC expression uniformly high across all CIC-rearranged sarcomas? Have the authors evaluated MYC-nonamplified CIC sarcoma models (if any, figure 6 suggests that there are MYC-nonamplified CIC-rearranged sarcomas)? If so, could they clarify whether the mechanism of action of CDK1/2/9 inhibitors differs in those settings compared with the models described in this study?

- We are grateful for this thoughtful question. Based on the literature, MYC expression seems to be universally high across *CIC*-rearranged sarcomas (Smith SC, et al. CIC-DUX sarcomas demonstrate frequent MYC amplification and ETS-family transcription factor expression. Mod Pathol 28, 57-68 (2015). Of the four cell line models available to us, one (i.e., CDS2) showed copy number gain of *MYC*, whereas the other 3 cell lines (i.e., CIC01, CIC02, and CDS1) did not show *MYC* copy number gain. We have added a statement to the Discussion to clarify that response to CDK2 and CDK9 inhibition we evaluated does not seem to depend on *MYC* copy number (lines: 395-401): Our genomic profiling studies demonstrated near-ubiquitous oncogenic activation of MYC in clinical samples of *CIC*-rearranged sarcoma, either directly or indirectly, through the genomic gain of *ETV4*. Of note, response to CDK2 and CDK9 inhibition seems to be independent of *MYC* copy number, as only CDS2 shows *MYC* copy number gain, whereas CIC01, CIC02, and CDS1 do not. These findings support the concept of MYC dependence in *CIC*-rearranged sarcoma and provide further justification for MYC-targeted treatment strategies.

13) Line 292: Fig 6A and 6B-The MYC amplification findings are intriguing, and we would welcome additional detail to better understand this observation. Specifically, could the authors clarify how many MYC copies are gained, and the nature of the amplification (e.g., extrachromosomal versus homogeneously staining regions, or another mechanism)? If FISH data are available, representative images would be especially valuable and would nicely complement the existing results.

- We thank the reviewer for this question. As mentioned in the manuscript (line 119), one of the cases had *MYC* amplification (seven copies) which was detected by our targeted NGS approach. Unfortunately, no material remains from this case that could be used for FISH analysis, so we cannot speculate on the nature of the amplification.

14) Related to Figure 6, while these data are intriguing, would there be a way to include discussion of these results earlier in the paper, perhaps more connected to description of clinically available data or characteristics of the patient-derived models. As presented, this doesn't feel particularly well connected to the rest of the paper.

- We appreciate the reviewer's suggestion and have introduced the genomic analysis of clinical cases, along with Figure 6 (now Figure 1), at the beginning of the Results (lines:107-124):

  Copy number gains of *ETV4* and *MYC* are recurrent events in *CIC*-rearranged sarcoma. We performed targeted NGS in 26 clinical cases of FISH-proven *CIC*-rearranged sarcoma to determine the frequency of aberrations that indirectly or directly activate MYC supporting a rationale for therapeutic targeting. FISH for *MYC* and centromere 8 was performed in a subset of two cases. The 26 patients comprised 9 males and 17 females, aged 6-72 (median, 35.5 years). Twenty-four tumors were primary tumors, and two were metastases. Upon manual review NGS uncovered a *CIC::DUX4* fusion in 24 cases, a *CIC::LEUTX* fusion in one case, and no definitive *CIC* rearrangement in one case. NGS identified genomic gains in the transcription

factor *ETV4* in 24/26 cases, including amplification (N=5), copy number gain (N=18), and subclonal gain (N=1) (**Fig. 1A**). In addition, NGS analysis identified genomic gain of *MYC* in 14/26 cases, including *MYC* amplification (seven copies) with concurrent gain of chromosome 8 (N=1), copy number gain of *MYC* only (N=1), copy number gain of *MYC* and chromosome 8 (N=10). Trisomy 8 was detected by FISH in two additional cases (N=2) (**Fig. 1B**). These findings are in line with the previously reported additional gain of chromosome 8 and *MYC* amplification in *CIC::DUX4*-rearranged sarcoma.[12] Taken together, these data support a rationale for evaluating MYC as an oncogenic target in *CIC*-rearranged sarcoma

All main figures have been renumbered accordingly.

15) Line 343: There is an "in" missing after the word "sensitivity".

- We thank the reviewer for carefully identifying this wording error. We have corrected the text in the Discussion (lines: 371-373): Of note, two recent studies identified sensitivity of *CIC*-rearranged sarcoma tumoroids to MCL-1 inhibitors which, however, have limited 7clinical utility due to cardiac side effects.[41, 42]

We have revised the section about CDK2 in the Introduction to increase clarity (lines: 95-101): CDK2-cyclin E was recently identified as a potential druggable target in *CIC*-rearranged sarcoma, with the pan-CDK inhibitor dinaciclib suppressing tumor growth and metastasis *in vivo*.[15] CDK2 belongs to the cyclin-dependent kinase family of Ser/Thr protein kinases. It binds cyclin E and regulates the transition from G1 to S phase; binding of CDK2 to cyclin A coordinates S phase progression. CDK2 can phosphorylate substrates involved in a range of physiological functions including the regulation of histone modifications, chromatin, transcription, and RNA/DNA metabolism.[16]

In addition, we have corrected the spelling of the MYC-MAX inhibitor from 10075-G5 to 10074-G5 and standardized nomenclature throughout the manuscript, figures, and supplementary materials. Specifically, we now use NUT to refer to the protein and *NUTM1* to refer to the gene, and Rb for the protein in accordance with accepted nomenclature conventions.

We further included a Human and Animal Ethics Statement to meet the journal's guidelines including IRB approval numbers and statements of accordance with relevant guidelines and regulations.

We would also like to request the addition of two coauthors, Dr. Timothy B. Branigan and Dr. Mohamed Alyousef, to the revised manuscript. Both individuals made substantial contributions to the additional experiments and analyses undertaken during the revision process. Written agreement from all authors regarding this authorship change has been obtained and is provided as a separate file.

We hope that our revisions have fully addressed the reviewers' comments and have strengthened both the rigor and clarity of the study. We believe that the revised manuscript is substantially improved and is now better aligned with the standards of the journal. We sincerely thank the editor and reviewers for their thoughtful feedback, careful evaluation, and the opportunity to revise our work. We appreciate your consideration of our revised submission and look forward to your assessment.

Very sincerely,

Inga-Marie Schaefer

Inga-Marie Schaefer, MD
