# Supplementary material for: Co-targeting CDK2 and CDK9 inhibits MYC oncogenic programs and has anti-tumor effects in CIC-rearranged sarcoma
Source: Commun Med (Lond). 2026 Oct 7;6:572. doi: 10.1038/s43856-026-01956-1 (PMC13728178; doi:10.1038/s43856-026-01956-1)
Supplement: Supplementary file 2 [file 43856_2026_1956_MOESM2_ESM.pdf]

SUPPLEMENTARY INFORMATION

Co-targeting CDK2 and CDK9 inhibits MYC oncogenic programs and has anti-tumor effects in *CIC*-rearranged sarcoma

Quentin Odom-Lewis[1], Samantha L. Davis[1], Stephan Pieper[1], Shannon M. R. Legge[1], Emilia F. A. Regenfuss[1], Nicolas Fernandez[2], Phillip D. Michaels[1], João A. Paulo[3], Steven P. Gygi[3], Chen Chu[4,5], Shanshan Zheng[4,5], Paola Dal Cin[1], Jason L. Hornick[1], Nicole L. Solimini[6,7,8], Timothy B. Branigan[6], Mohamed Alyousef[1], Emanuele Mazzola[9], Ewa T. Sicinska[10], Karen Bui[6], Prafulla C. Gokhale[6], Christopher A. French[1], George D. Demetri[6,7,8,11], Geoffrey I. Shapiro[6,8,11], and Inga-Marie Schaefer[1]

[1] Department of Pathology, Brigham and Women's Hospital, Harvard Medical School, Boston, Massachusetts, USA

[2] Broad Institute of MIT and Harvard, Cambridge, Massachusetts, USA

[3] Department of Cell Biology, Harvard Medical School, Boston, Massachusetts, USA

[4] Department of Cancer Biology, Dana-Farber Cancer Institute, Boston, Massachusetts, USA

[5] Department of Genetics, Blavatnik Institute, Harvard Medical School, Boston, MA, USA

[6] Department of Medical Oncology, Experimental Therapeutics Core, Dana-Farber Cancer Institute, Harvard Medical School, Boston, Massachusetts, USA

[7] Center for Sarcoma and Bone Oncology, Dana-Farber Cancer Institute, Boston, Massachusetts, USA

[8] Ludwig Center at Harvard, Boston, Massachusetts, USA

[9] Department of Data Science, Dana-Farber Cancer Institute, Boston, Massachusetts, USA

[10] Department of Pathology, Dana-Farber Cancer Institute, Harvard Medical School, Boston, Massachusetts, USA

[11] Department of Medicine, Brigham and Women's Hospital, Harvard Medical School, Boston, Massachusetts, USA

CORRESPONDING AUTHOR

Inga-Marie Schaefer, MD

Department of Pathology, Brigham and Women's Hospital

75 Francis Street, Boston, Massachusetts 02115, USA

Tel: +1 617-525-7447; Fax: +1 617-277-9015;

E-mail: ischaefer@bwh.harvard.edu

ORCID: https://orcid.org/0000-0001-9710-5500

SUPPLEMENTARY INFORMATION - Table of Contents

Supplementary Figure S1. Detection of *CIC* fusions in *CIC*-rearranged sarcoma cell lines by targeted NGS.

Supplementary Figure S2. Uncropped blots for Figure 2c.

Supplementary Figure S3. Uncropped blots for Figure 2d.

Supplementary Figure S4. *In vitro* efficacy of selective CDK2 inhibition.

Supplementary Figure S5. Uncropped blots for Supplementary Figure S4d.

Supplementary Figure S6. Uncropped blots for Figure 3d.

Supplementary Figure S7. Uncropped blots for Figure 3h.

Supplementary Figure S8. CDK2/4/6 but not CDK4/6 inhibition has anti-tumor effects *in vitro*.

Supplementary Figure S9. Common significantly down- and upregulated genes in CIC01 and CIC02.

Supplementary Figure S10. Direct MYC inhibition has anti-tumor effects in *CIC*-rearranged sarcoma.

Supplementary Figure S11. Uncropped blots for Figure 5g.

Supplementary Figure S12. Uncropped blots for Figure 5h.

Supplementary Figure S13. Time course for KB-0742 treatment in CDS2.

Supplementary Figure S14. Uncropped blots for Supplementary Figure S13.

Supplementary Figure S15. Dose response matrices for combined CDK2 and CDK9 inhibition.

Supplementary Figure S16. Uncropped blots for Figure 6c.

Supplementary Figure S17. Uncropped blots for Figure 6d.

Supplementary Figure S18. Uncropped blots for Figure 6e.

Supplementary Figure S19. Uncropped blots for Figure 6f.

Supplementary Figure S20. Uncropped blots for Figure 6g.

Supplementary Table S1.

Supplementary Table S2.

Supplementary Table S3.

Supplementary Table S4.

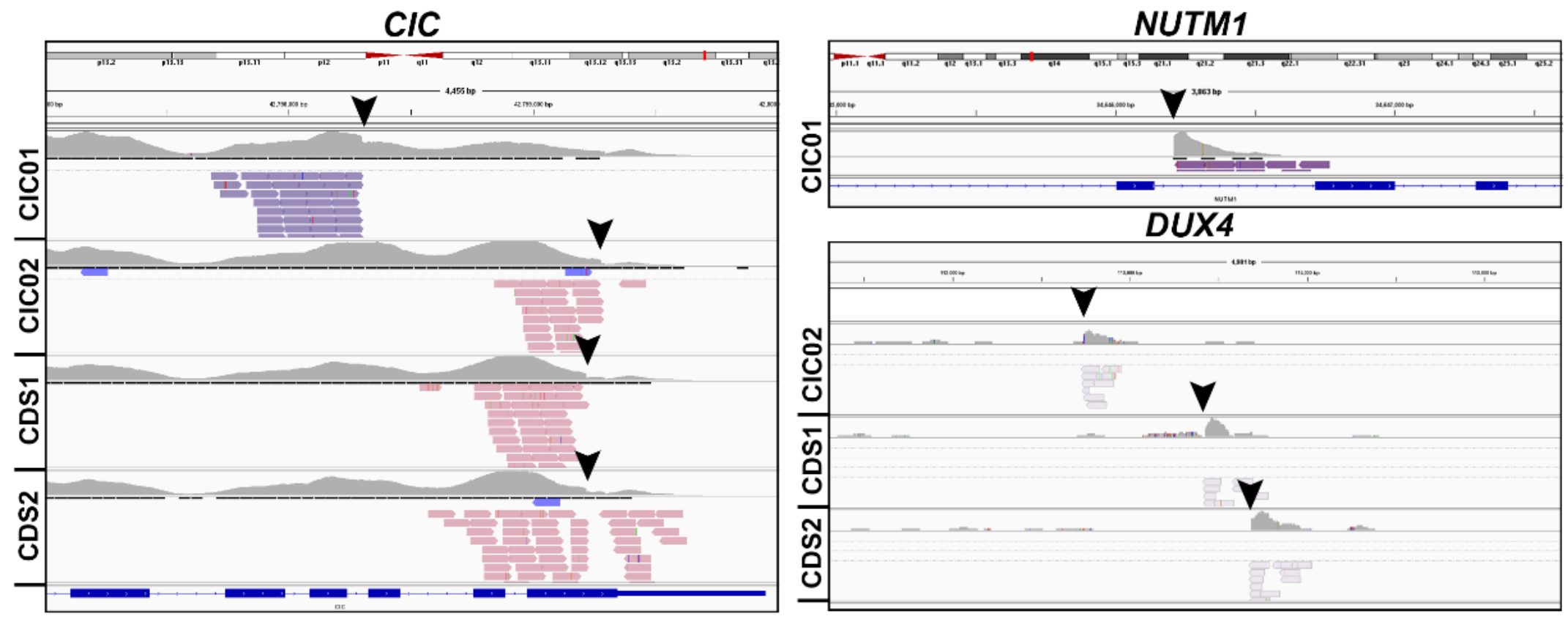

Supplementary Figure S1

Detection of *CIC* fusions in *CIC*-rearranged sarcoma cell lines by targeted NGS.

Targeted next-generation sequencing (NGS) detected a rearrangement between *CIC* (NM_001386298.1) intron 18 (hg19 breakpoint: chr19:42,294,150) and *NUTM1* (NM_001284292.2) intron 5 (hg19 breakpoint: chr15:34,354,009) in CIC01 and a rearrangement between *CIC* (NM_001386298.1) exon 21 (hg19 breakpoint: chr19:42,295,124) and *DUX4* (NM_001306068.3) exon 1 (hg19 breakpoint: chr4:190,173,906) in CIC02. Previously reported *CIC::DUX4* fusions were confirmed in CDS1 and CDS2. Breakpoints are indicated by arrowheads.

Supplementary Figure S2

Uncropped blots for Figure 2c.

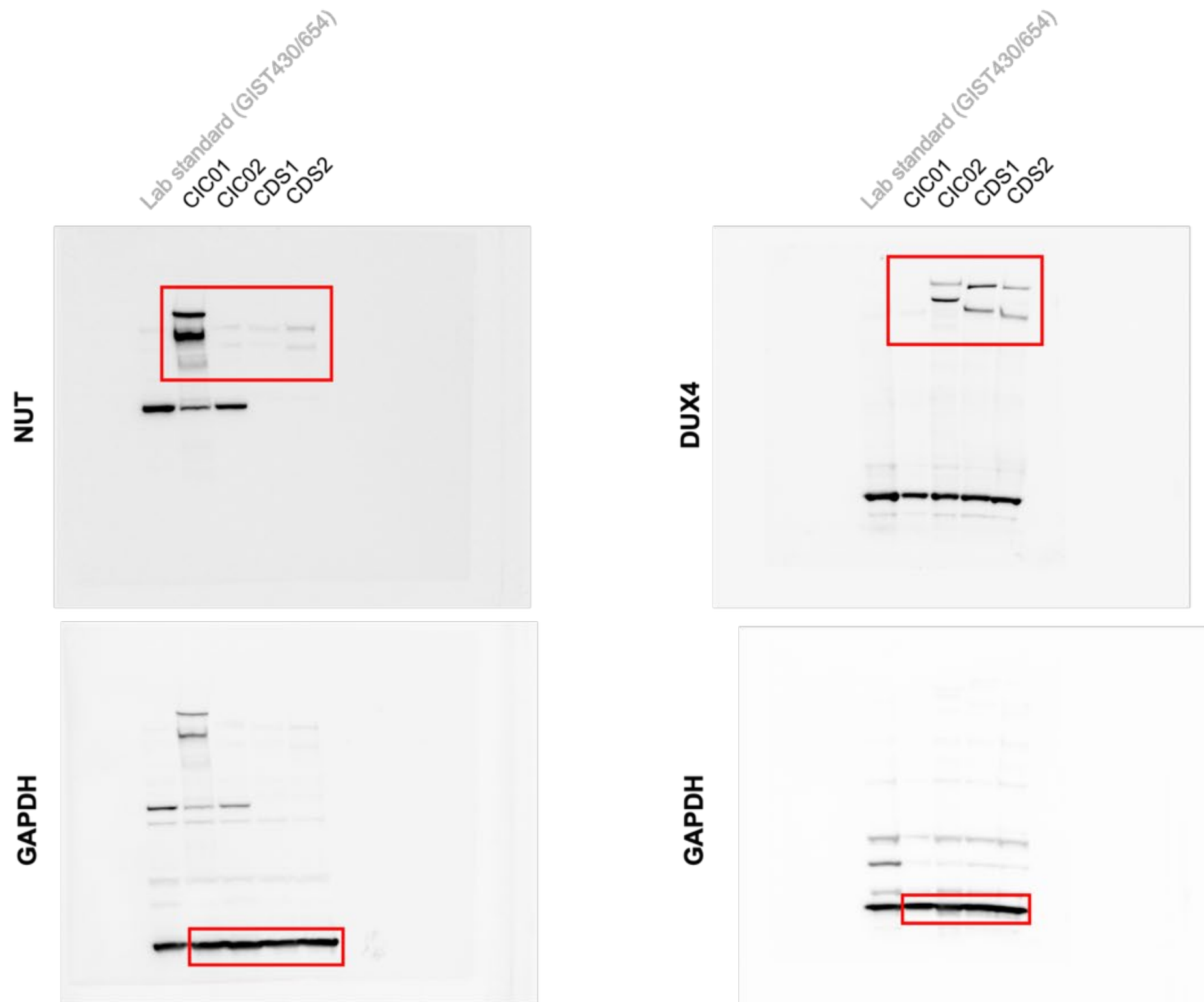

Note: Lane labels in gray identify lanes that were present on the original blot but were not displayed in the corresponding figure in the manuscript.

Supplementary Figure S3

Uncropped blots for Figure 2d.

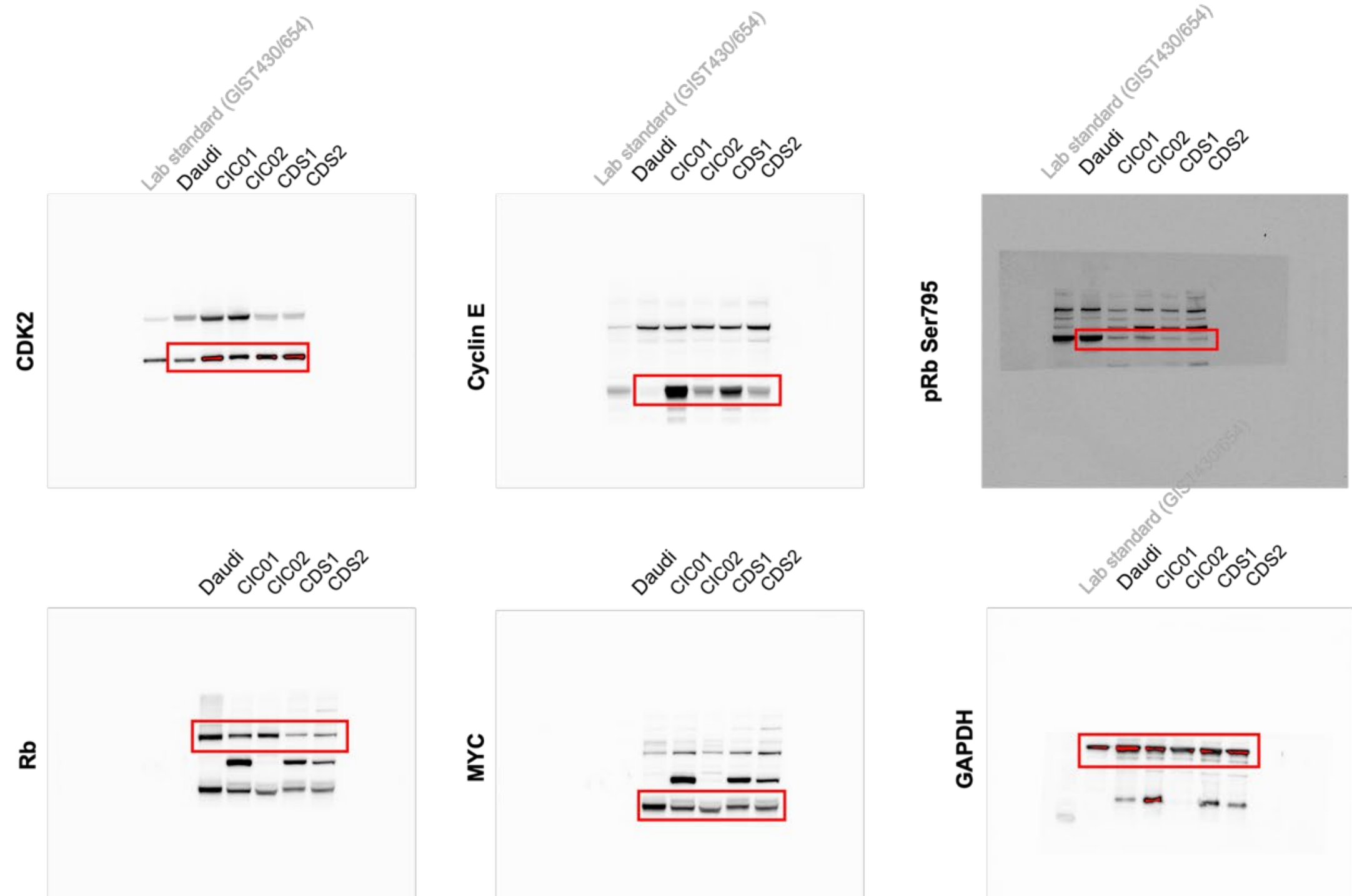

Note: Lane labels in gray identify lanes that were present on the original blot but were not displayed in the corresponding figure in the manuscript.

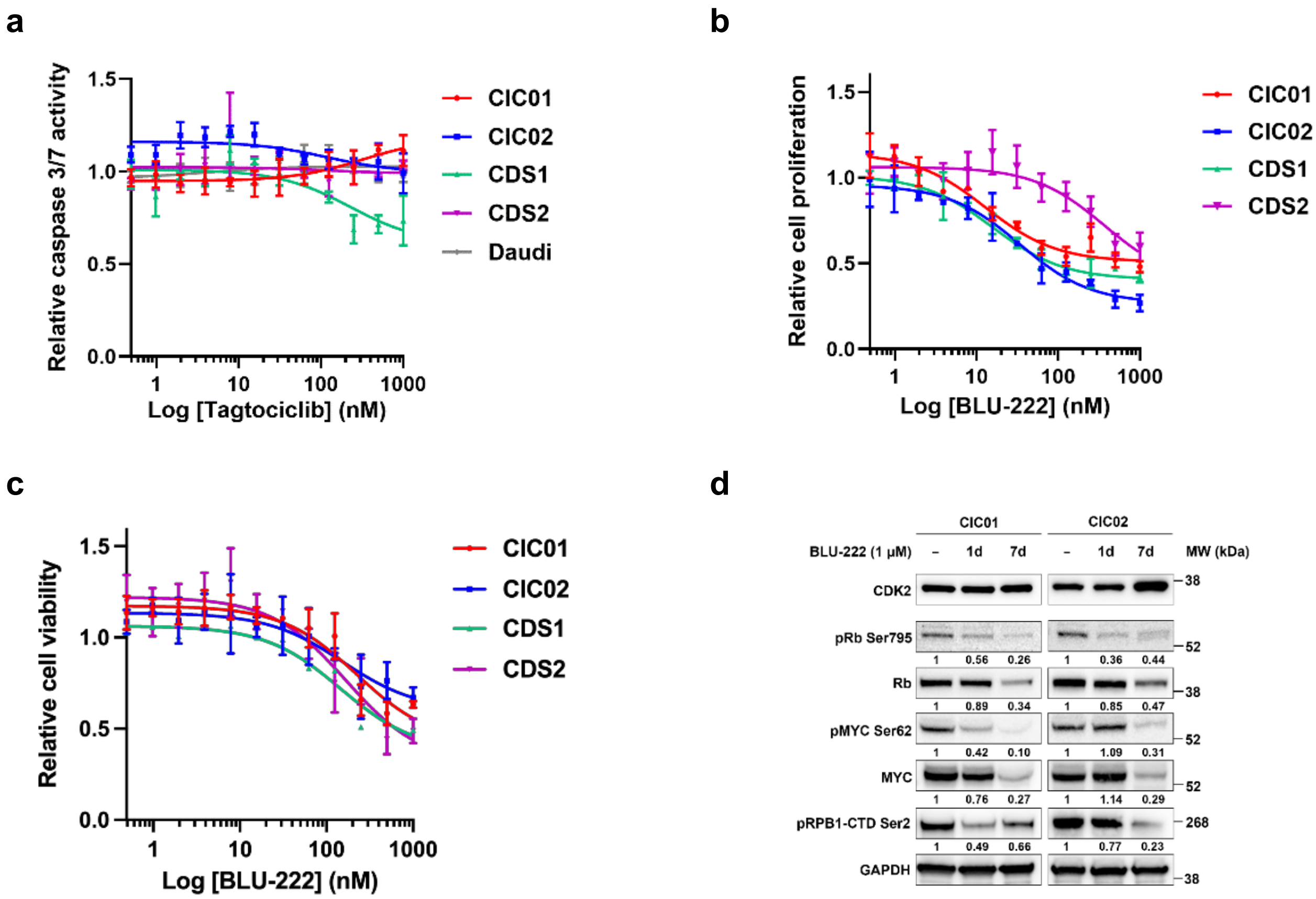

Supplementary Figure S4

***In vitro* efficacy of selective CDK2 inhibition.**

(**a**) Tagtociclib does not lead to increased apoptosis as assessed by Caspase-Glo 3/7 assay in the CIC01, CIC02, CDS1, and CDS2 cell lines (normalized to DMSO). Tests were performed in triplicate (N=3 biologically independent samples; mean +/- s.d). The selective CDK2 inhibitor BLU-222 reduces cell proliferation as assessed by BrdU incorporation at 24 h **(b)** and cell viability as assessed by CellTiter-Glo assay **(c)** in the CIC01, CIC02, CDS1, and CDS2 cell lines (normalized to DMSO). Tests were performed in triplicate (N=3 biologically independent samples; mean +/- s.d). **(d)** Immunoblotting analyses show that BLU-222 treatment inhibits the expression of phosphor-Rb Ser795, phospho-MYC Ser62, total MYC, and phospho-RPB1-carboxyl-terminal domain (CTD) Ser2 in the CIC01 and CIC02 cell lines. This effect is more pronounced after 7 days of treatment than after 1 day of treatment. CDK2 expression remains unchanged. The calibrated expression is shown at the bottom of each row. GAPDH was used as the loading control.

Supplementary Figure S5

Uncropped blots for Supplementary Figure S4d.

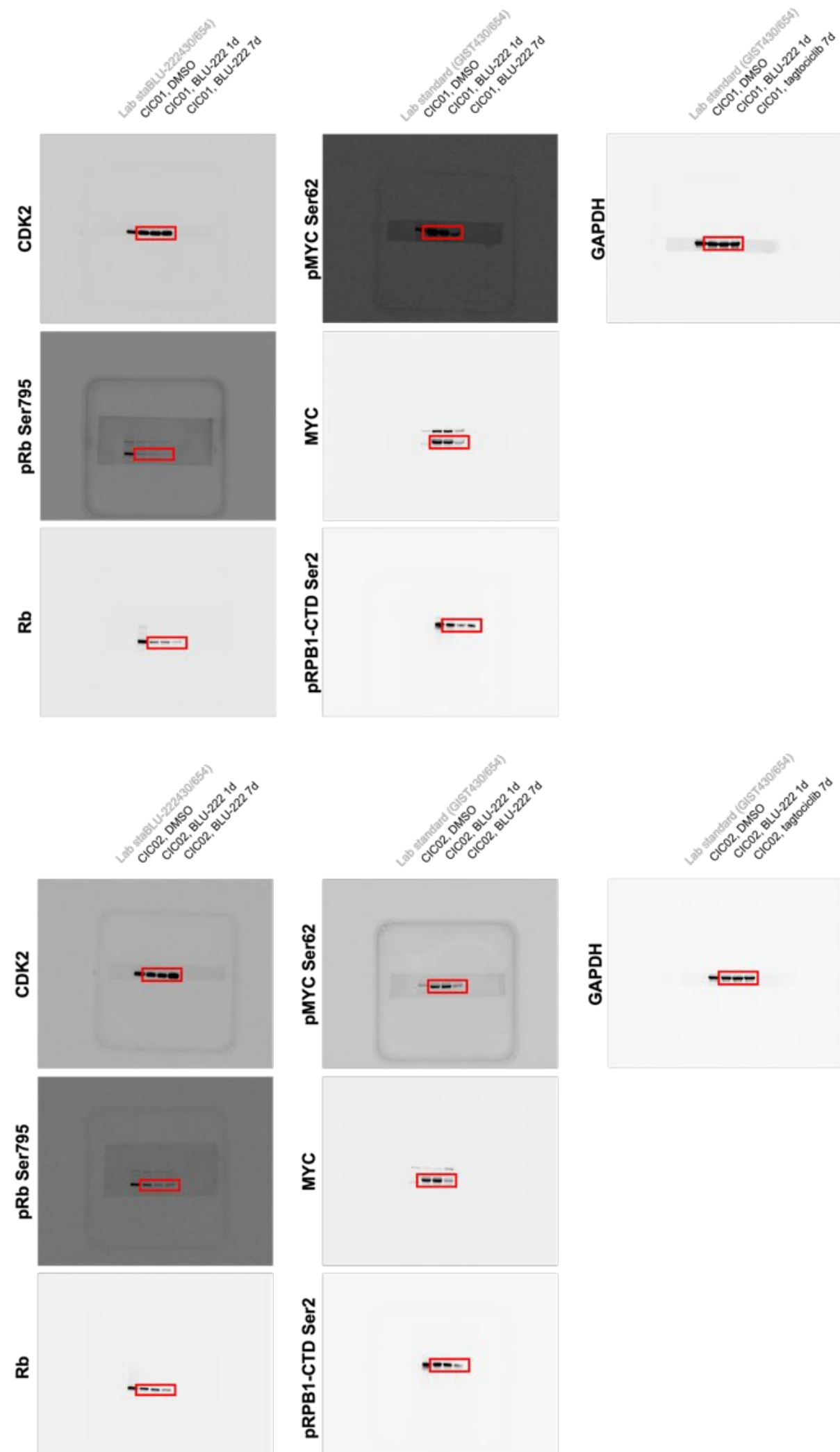

Note: Lane labels in gray identify lanes that were present on the original blot but were not displayed in the corresponding figure in the manuscript.

Supplementary Figure S6

Uncropped blots for Figure 3d.

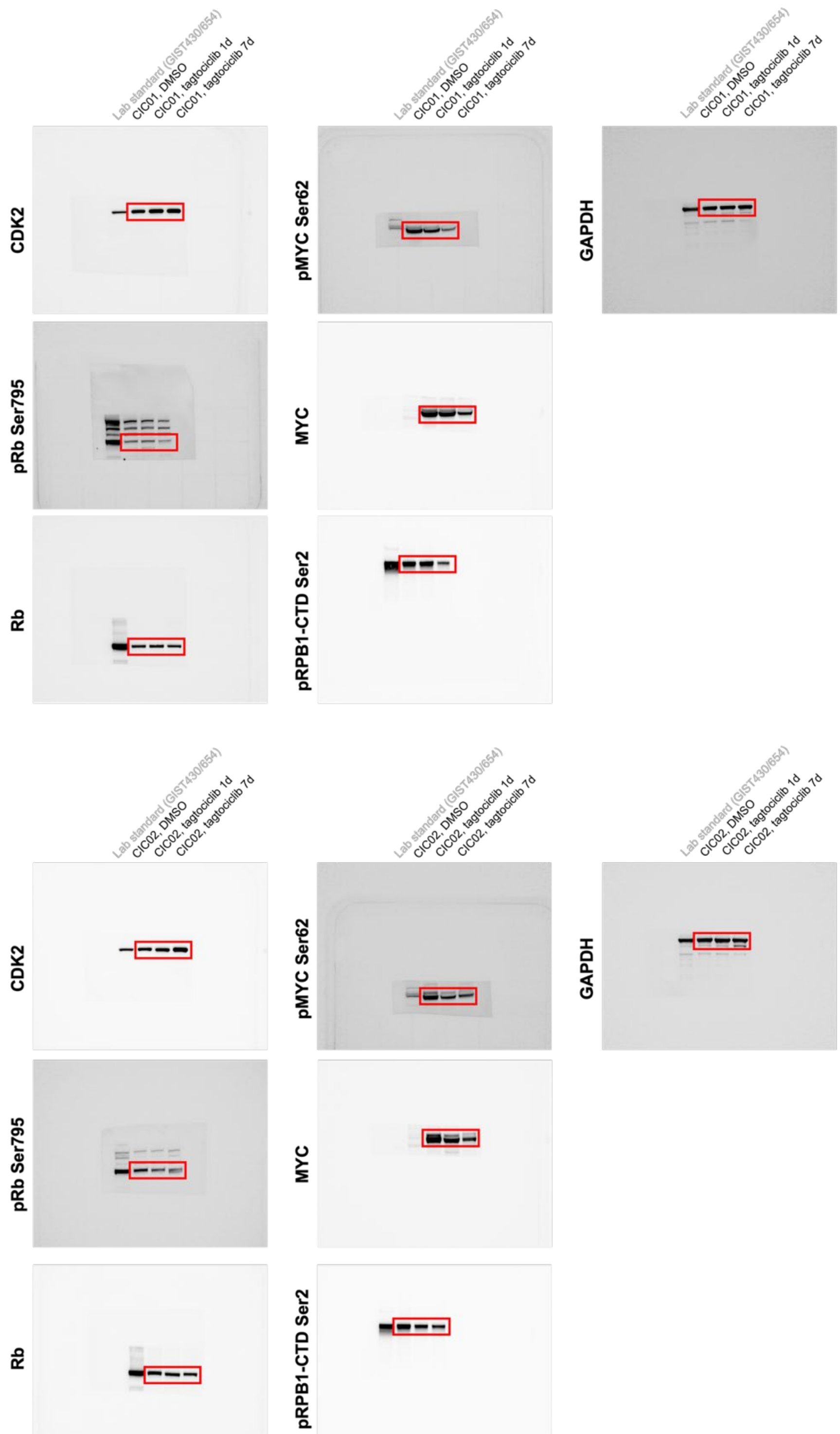

Note: Lane labels in gray identify lanes that were present on the original blot but were not displayed in the corresponding figure in the manuscript.

Supplementary Figure S7

Uncropped blots for Figure 3h.

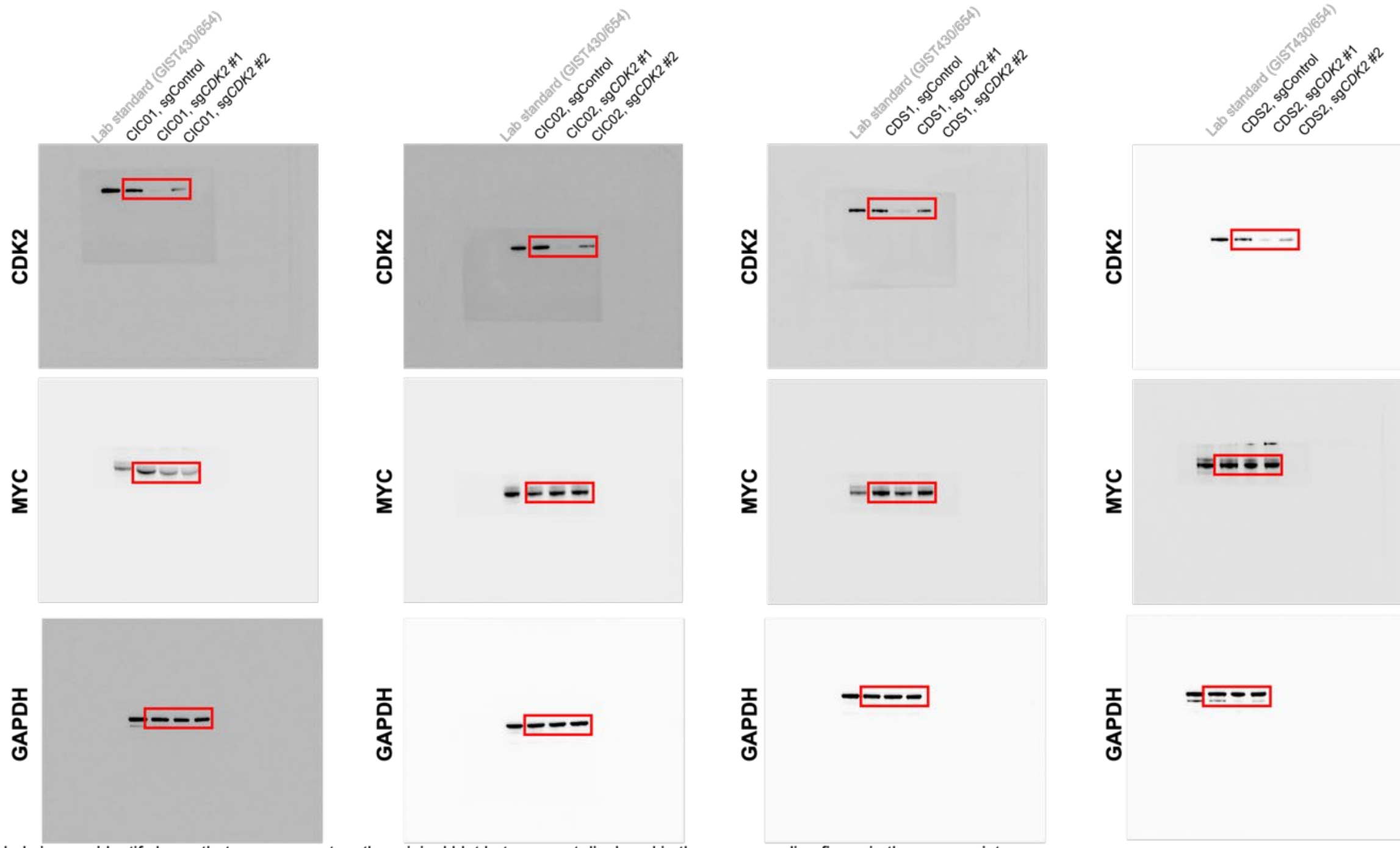

Note: Lane labels in gray identify lanes that were present on the original blot but were not displayed in the corresponding figure in the manuscript.

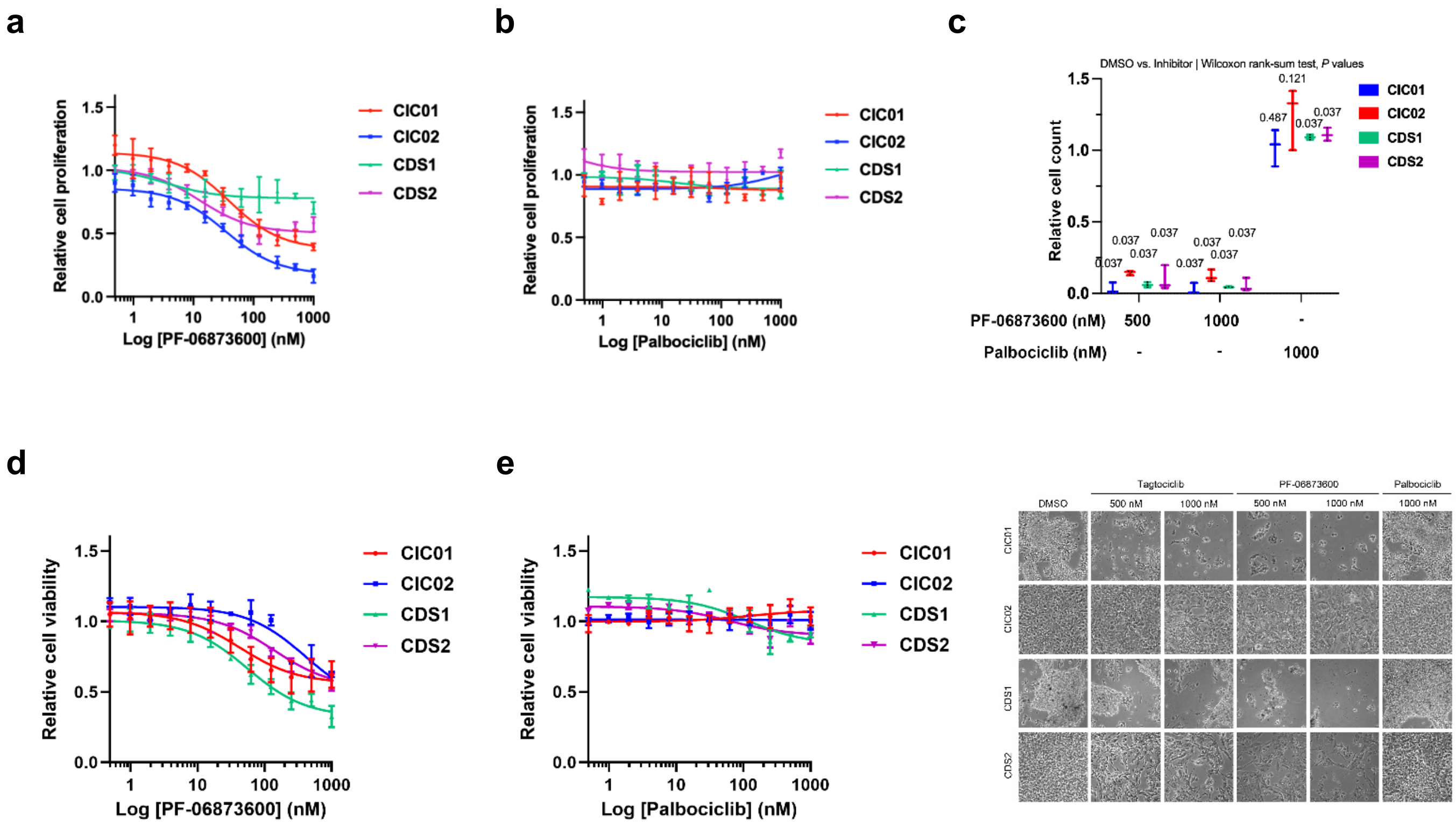

Supplementary Figure S8

CDK2/4/6 but not CDK4/6 inhibition has anti-tumor effects *in vitro*.

The CDK2/4/6 inhibitor PF-06873600 **(a)** but not the CDK4/6 inhibitor palbociclib **(b)** reduces cell proliferation as assessed by BrdU incorporation at 24 h (normalized to DMSO). **(c)** PF-06873600 but not palbociclib reduces cell numbers 7 days after treatment as shown by cell counts (*P*=0.037, each for PF-06873600; *P*=0.487, *P*=0.121, *P*=0.037, and *P*=0.037 for palbociclib; two-sided Wilcoxon rank-sum test); (top) and representative phase contrast images (bottom). Tests were performed in triplicate (N=3 biologically independent samples; mean +/- s.d). PF-06873600 **(d)** but not palbociclib **(e)** reduces cell viability as assessed by CellTiter-Glo assay in the CIC01, CIC02, CDS1, and CDS2 cell lines (normalized to DMSO). Tests were performed in triplicate (N=3 biologically independent samples; mean +/- s.d).

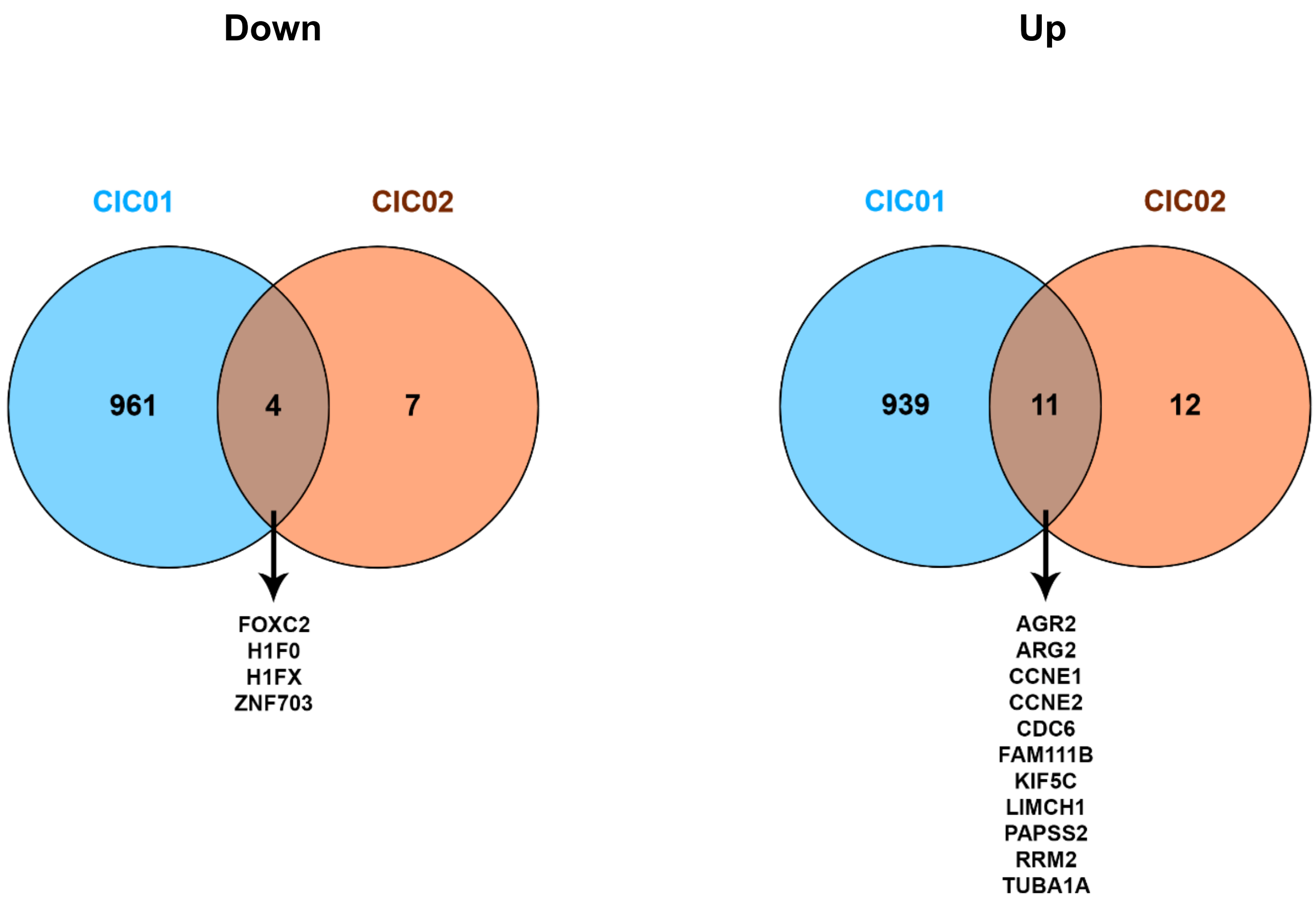

Supplementary Figure S9

Common significantly down- and upregulated genes in CIC01 and CIC02.

Venn diagram showing significantly down- and upregulated genes common to both cell lines as assessed by RNA sequencing analysis after 48 hours of treatment with tagtociclib.

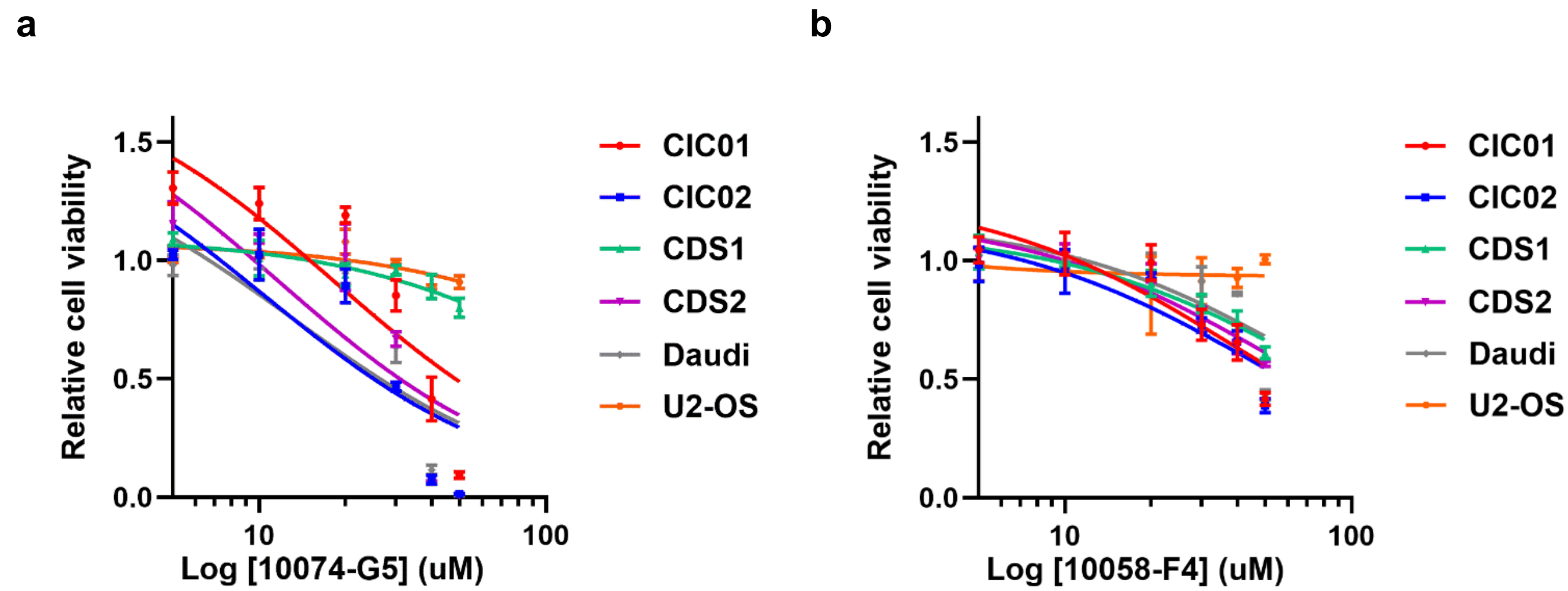

Supplementary Figure S10

Direct MYC inhibition has anti-tumor effects in *CIC*-rearranged sarcoma.

The MYC-MAX dimerization inhibitors 10074-G5 **(a)** and 10058-F4 **(b)** inhibit cell viability as assessed by CellTiter-Glo assay in the CIC01, CIC02, CDS1, and CDS2 cell lines as well as in Daudi control but not in U2-OS (normalized to DMSO). Tests were performed in triplicate (N=3 biologically independent samples; mean +/- s.d).

Supplementary Figure S11

Uncropped blots for Figure 5g.

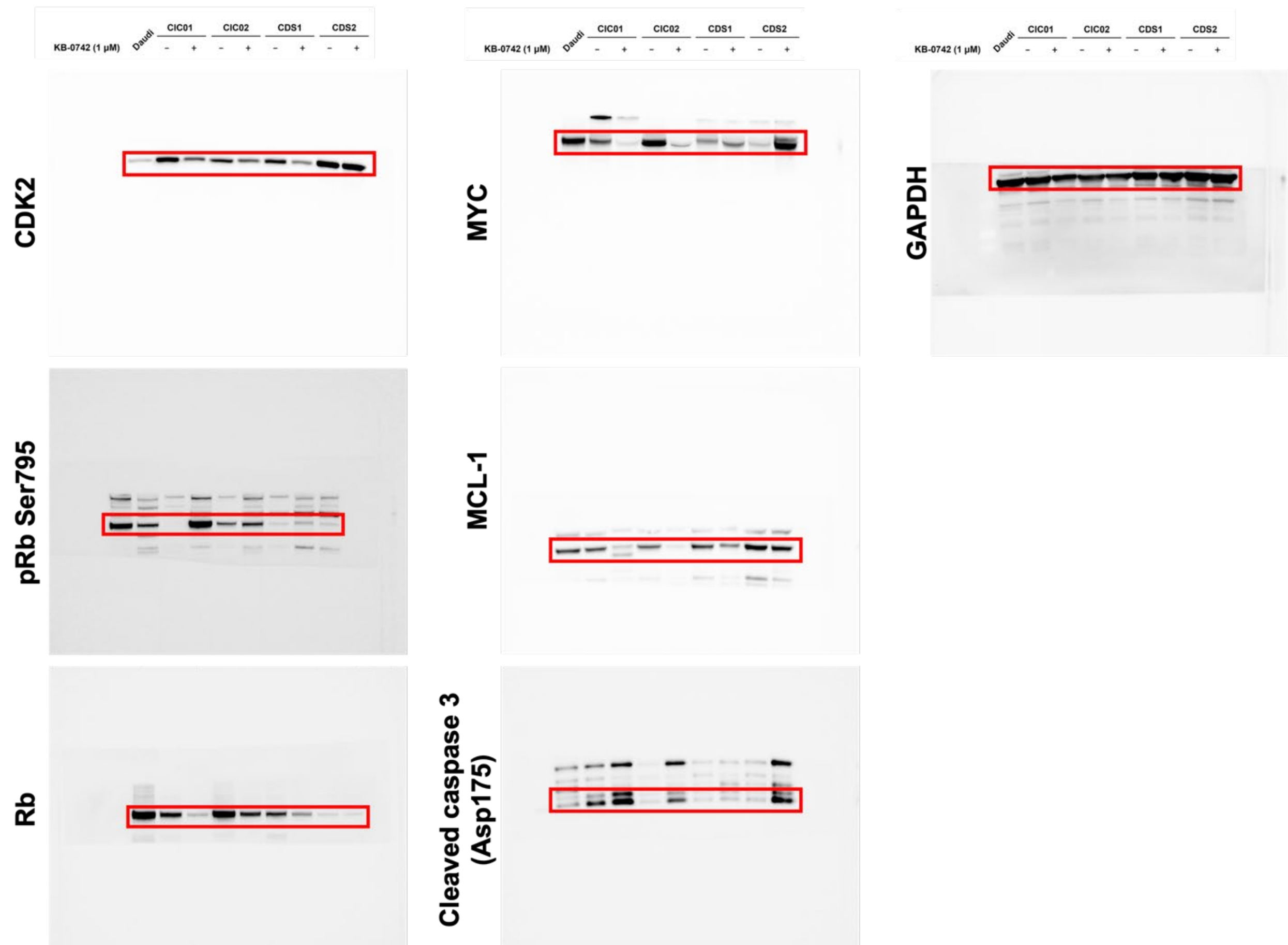

Supplementary Figure S12

Uncropped blots for Figure 5h.

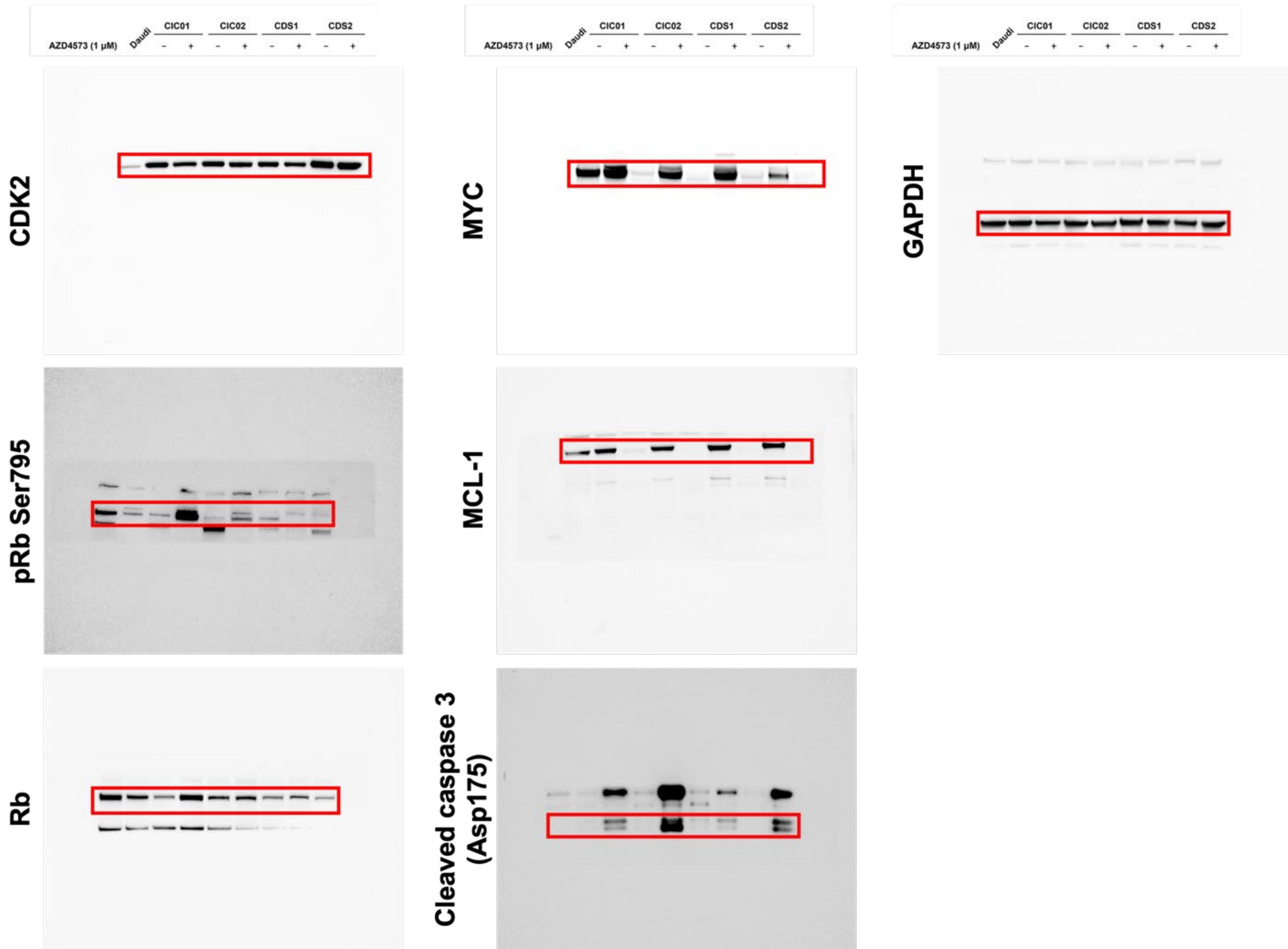

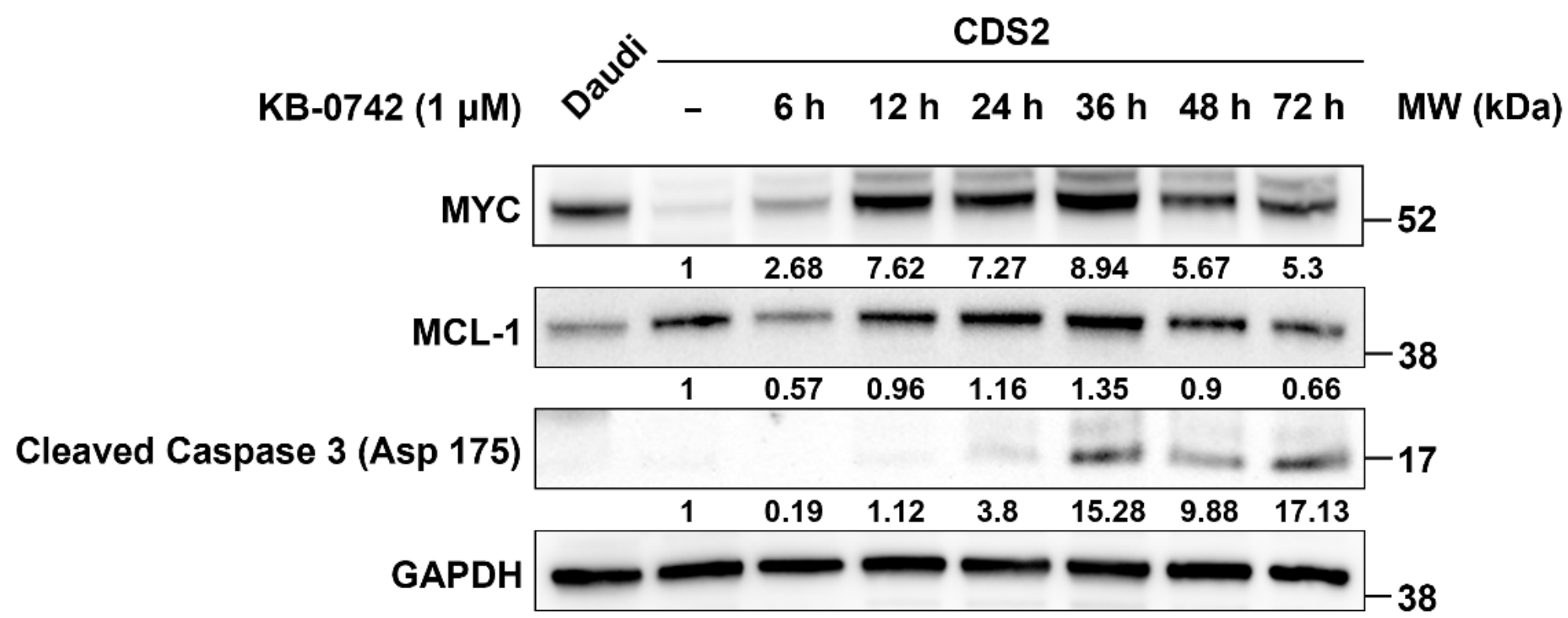

Supplementary Figure S13

Time course for KB-0742 treatment in CDS2.

Immunoblotting studies in the CDS2 cell line after 6, 12, 24, 36, 48, and 72 hours of treatment with KB-0742 (1 µM) demonstrate upregulation of MYC and MCL-1 expression peaking at 36 hours, and delayed increase of cleaved caspase 3 (Asp175) levels peaking at 72 hours. Calibrated expression shown at the bottom of each row. The 17-kDa band was used for calibration of cleaved caspase 3 (Asp175). GAPDH serves as loading control.

Supplementary Figure S14

Uncropped blots for Supplementary Figure S13.

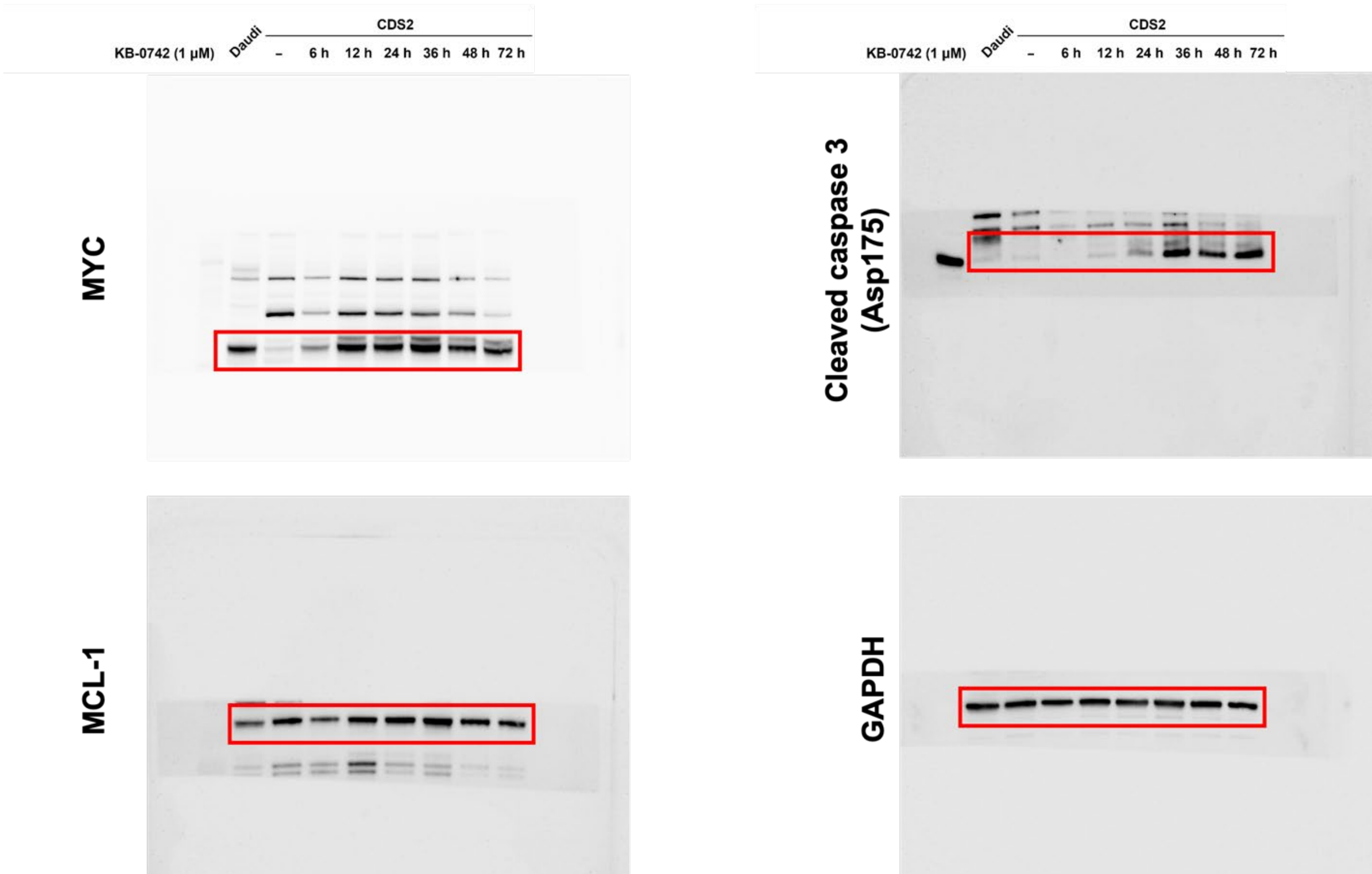

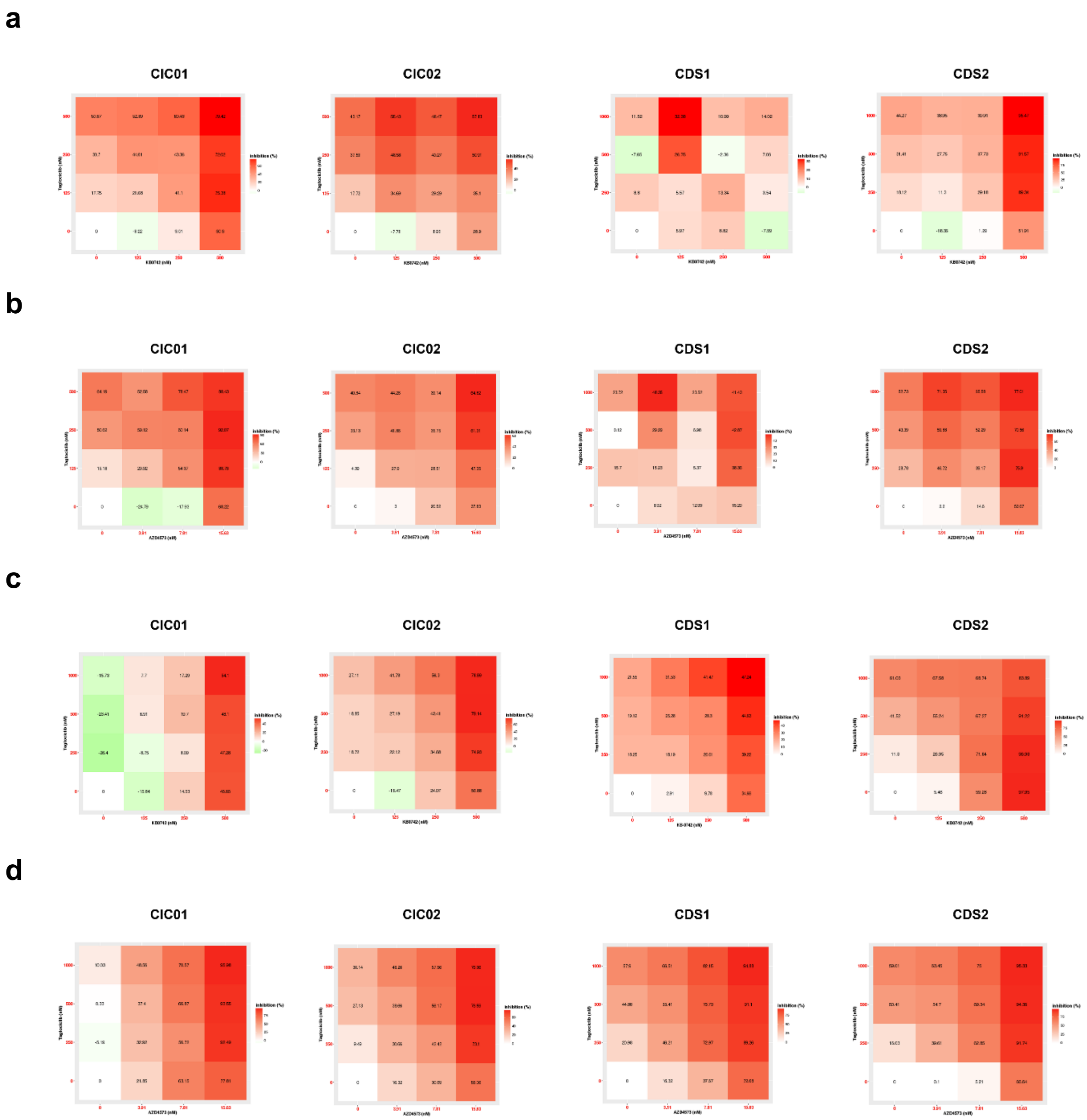

Supplementary Figure S15

Dose response matrices for combined CDK2 and CDK9 inhibition.

Dose response matrices for the combination effect of tagtociclib and KB-0742 as measured by cell proliferation **(a)** and cell viability **(b)** as well as the combination effect of tagtociclib and AZD4573 as measured by cell proliferation **(c)** and cell viability **(d)** in the CIC01, CIC02, CDS1, and CDS2 cell lines.

Supplementary Figure S16

Uncropped blots for Figure 6c.

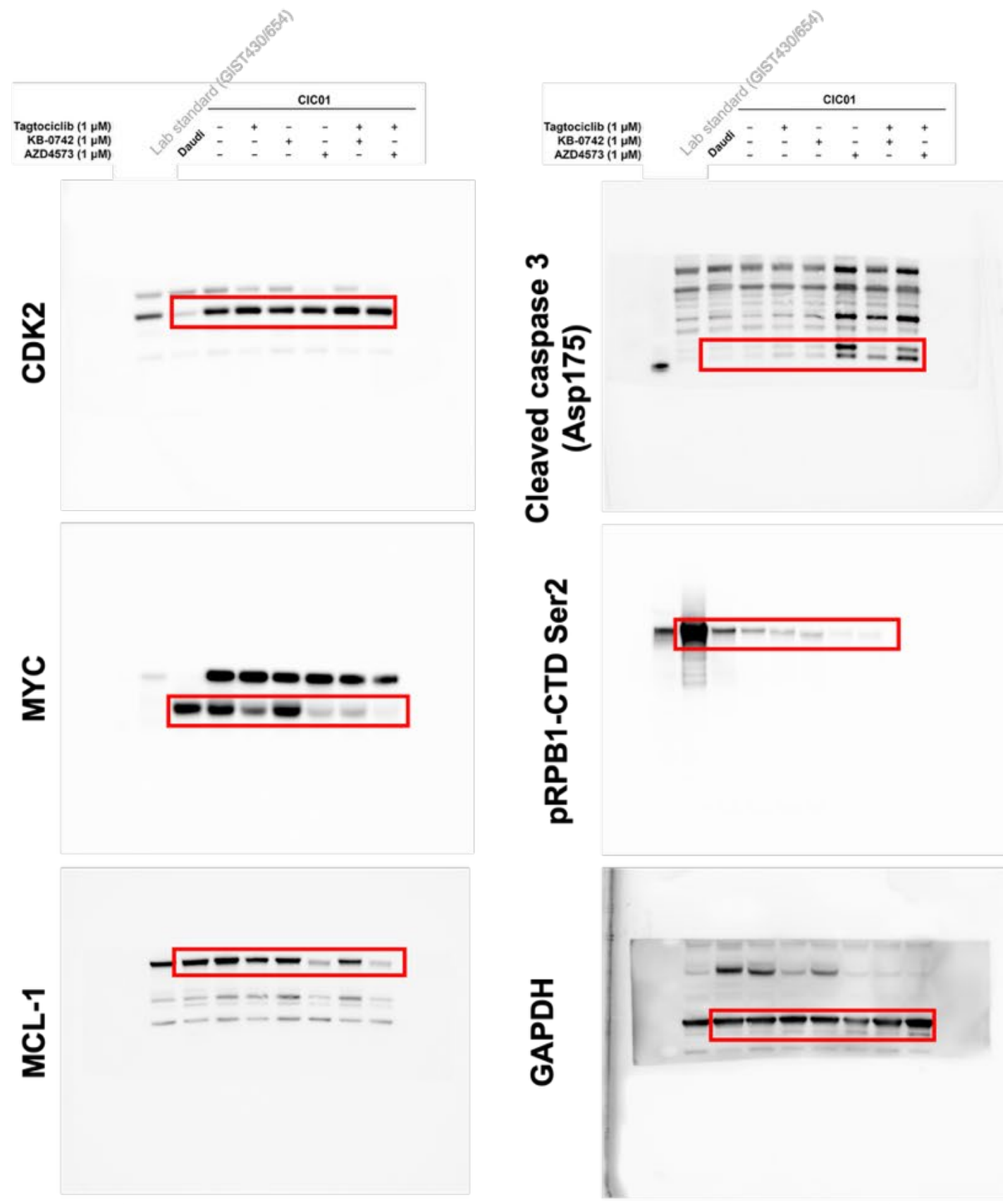

Note: Lane labels in gray identify lanes that were present on the original blot but were not displayed in the corresponding figure in the manuscript.

Supplementary Figure S17

Uncropped blots for Figure 6d.

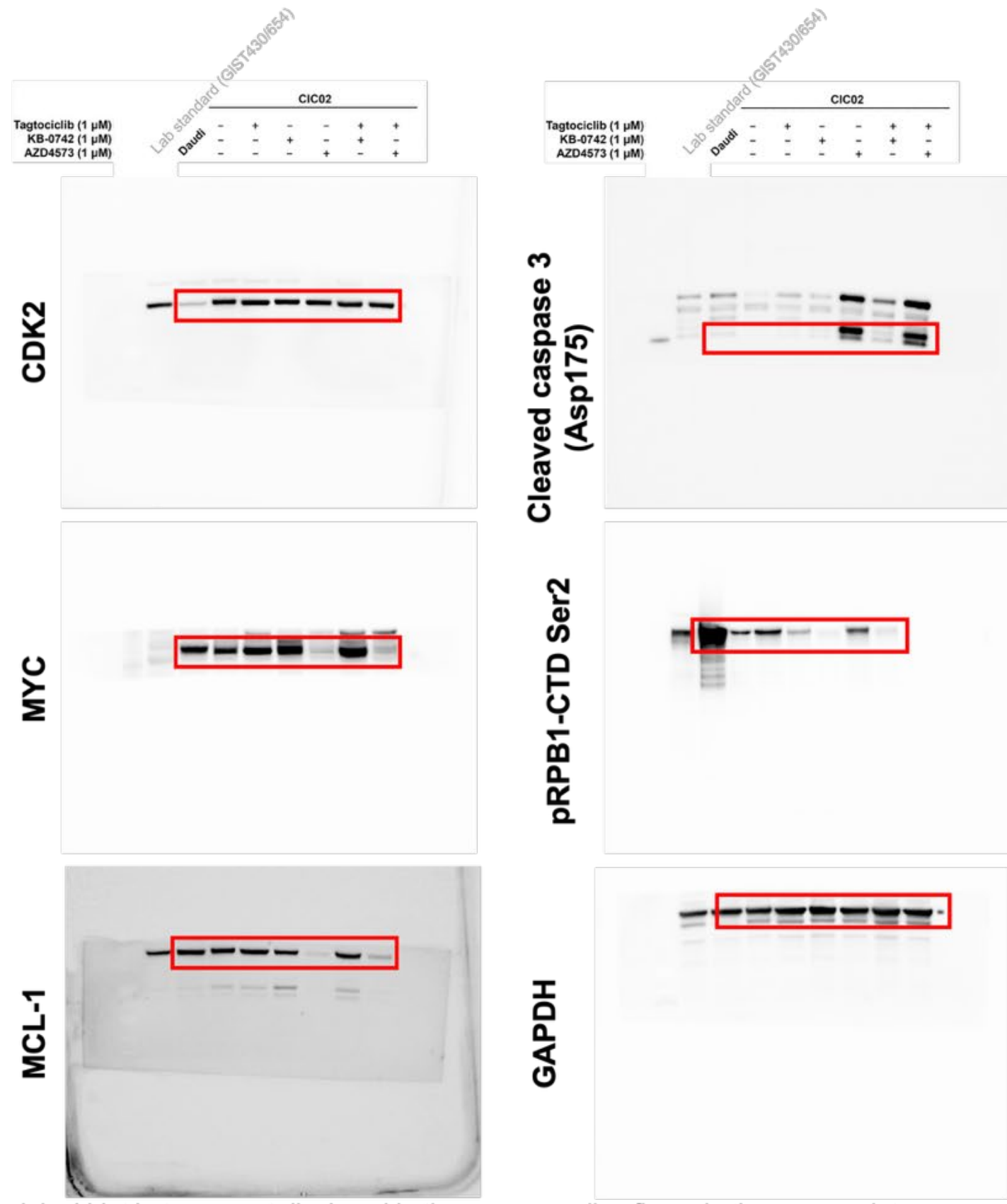

Note: Lane labels in gray identify lanes that were present on the original blot but were not displayed in the corresponding figure in the manuscript.

Supplementary Figure S18

Uncropped blots for Figure 6e.

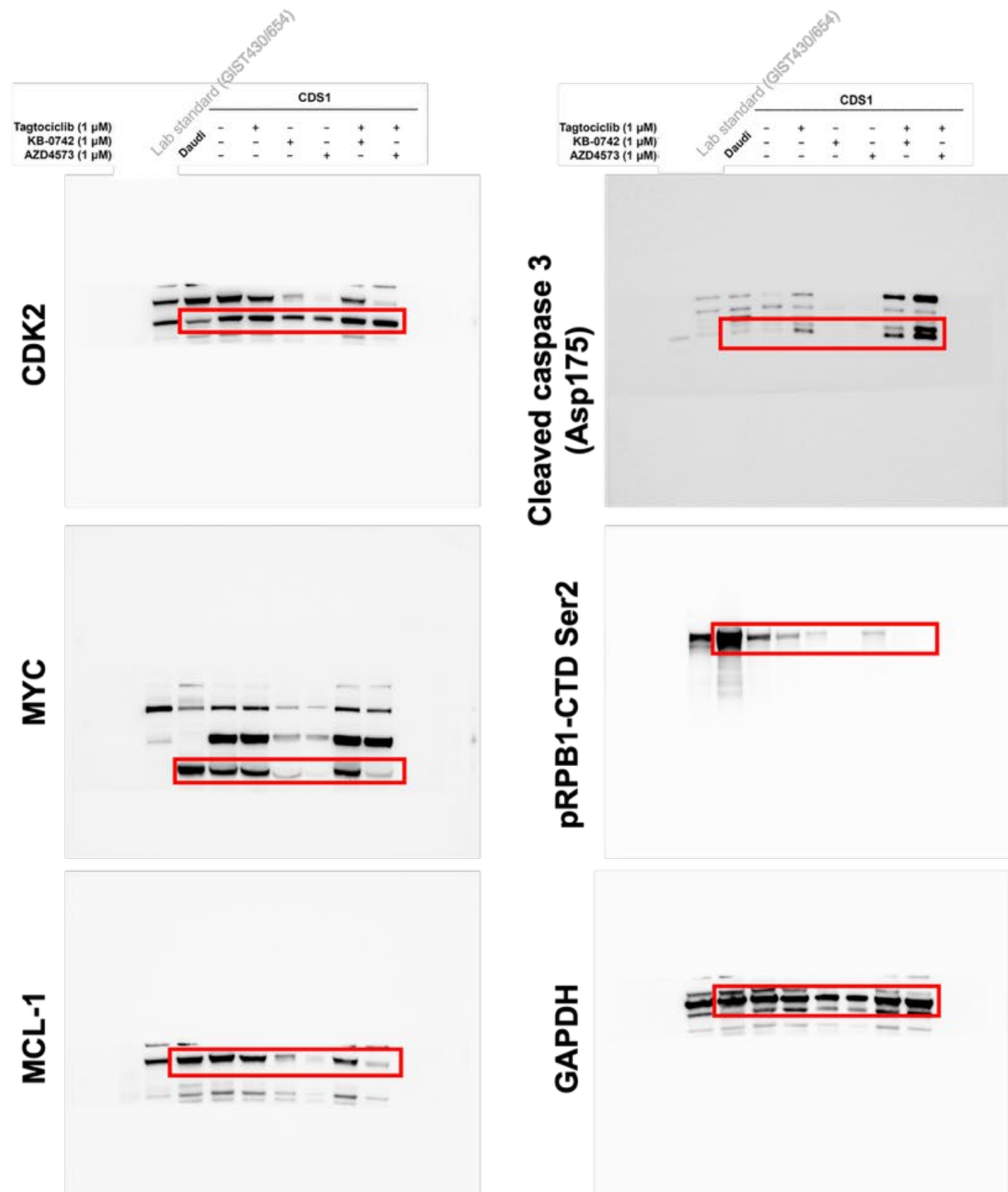

Note: Lane labels in gray identify lanes that were present on the original blot but were not displayed in the corresponding figure in the manuscript.

Supplementary Figure S19

Uncropped blots for Figure 6f.

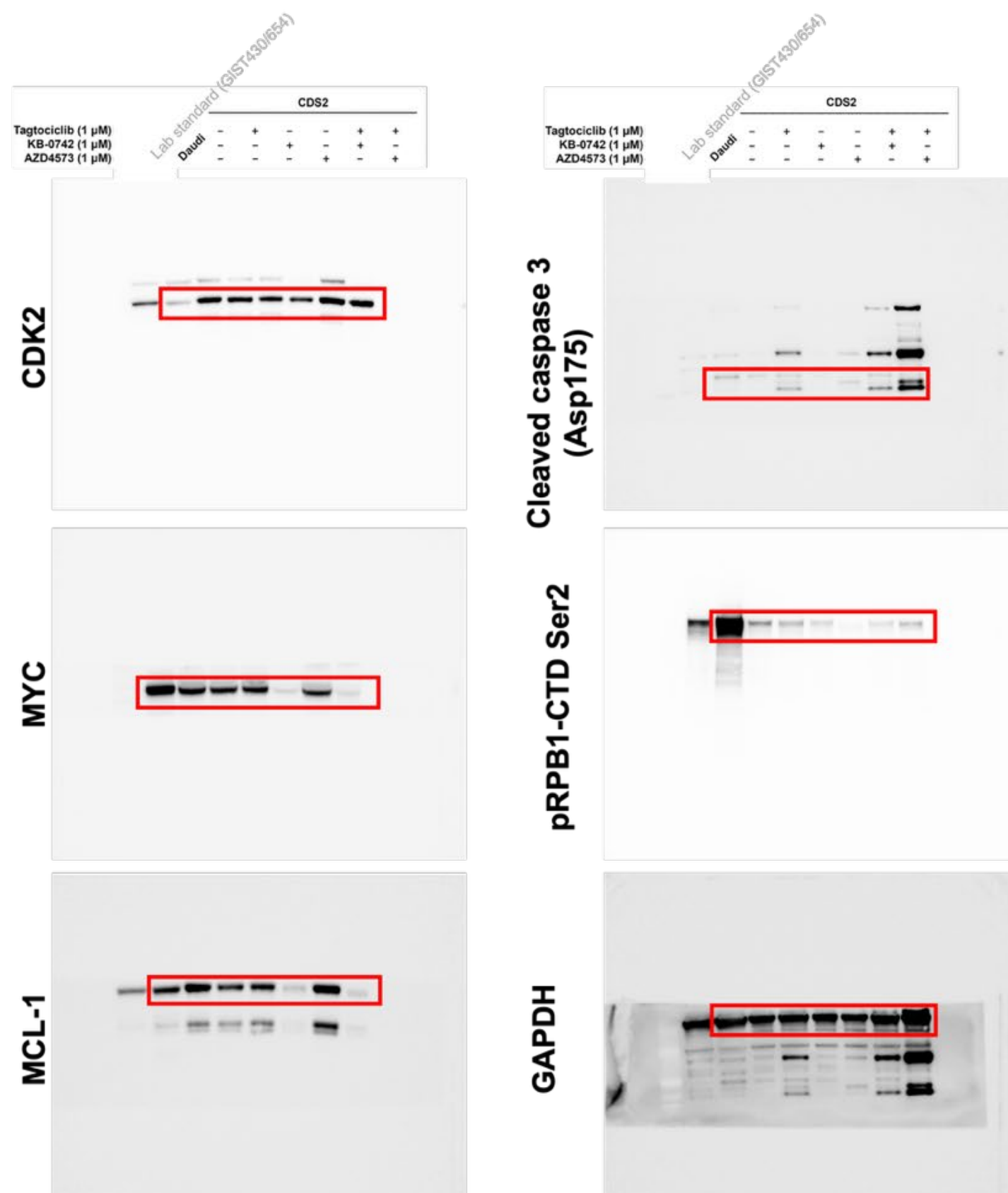

Note: Lane labels in gray identify lanes that were present on the original blot but were not displayed in the corresponding figure in the manuscript.

Supplementary Figure S20

Uncropped blots for Figure 6g.

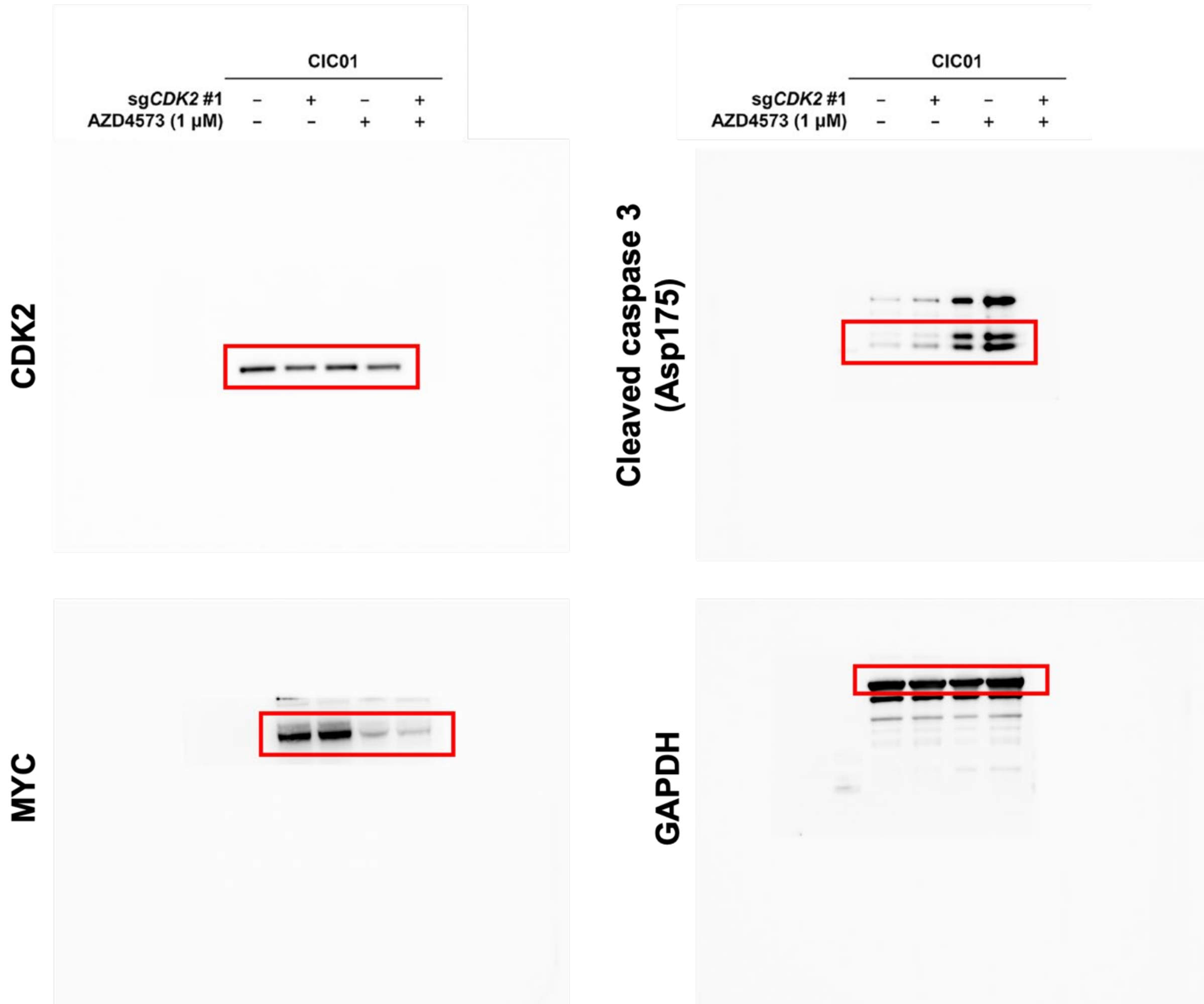

Supplementary Table S1

Proliferation IC_{50} values of CDK2 and CDK9 inhibitor drugs in *CIC*-rearranged sarcoma cell lines.

	IC_{50} value [nM] (95% CI)					
**Cell line**	**Tagtociclib**	**KB-0742**	**AZD4573**	**BLU-222**	**PF-06873600**	**Palbociclib**
CIC01	71.32 (52.17-97.74)	589.6 (325.4-760.3)	20.49 (14.56-28.56)	12.88 (9.103-18.09)	39.12 (30.02-50.99)	Not reached
CIC02	45.6 (35.50-58.33)	744.4 (499.7-1089)	10.56 (8.110-13.65)	33.74 (23.29-48.98)	34.39 (26.23-45.03)	Not reached
CDS1	125.4 (89.91-176.8)	524.6 (238.3-935.9)	1.16 (0.8552-1.539)	15.86 (11.97-20.95)	3.505 (1.101-10.68)	Not reached
CDS2	23.91 (10.20-54.42)	304.1 (197.3-349.5)	5.406 (3.813-7.555)	369.3 (177.4-896.6)	12.57 (8.960-17.48)	Not reached
Daudi	824.7 (402.0-2444)	1066 (548.3-1392)	18.65 (13.60-25.53)	-	-	-
U2-OS	-	1187 (919.0-1494)	38.93 (31.26-48.4)	-	-	-

Supplementary Table S2

RNA sequencing results for significantly downregulated genes as part of the transcription factor PPI library in CIC01 (a) and CIC02 (b).

a

Index	Name	*P* value	Adjusted *P* value	Odds Ratio	Combined score
1	ESR1	1.154E-15	2.875E-13	2.70	93.02
2	MYC	6.096E-12	7.589E-10	2.33	60.22
3	ILF3	3.869E-10	3.212E-08	3.35	72.63
4	FOXP3	1.844E-08	1.110E-06	2.85	50.67
5	ESR2	2.229E-08	1.110E-06	2.79	49.07
6	POU5F1	1.597E-07	6.627E-06	2.72	42.55
7	ILF2	2.458E-07	8.744E-06	3.46	52.67
8	TCF3	4.024E-07	1.164E-05	2.99	44.00
9	EP300	4.206E-07	1.164E-05	2.35	34.47
10	POLR2A	5.425E-07	1.351E-05	3.00	43.34

b

Index	Name	*P* value	Adjusted *P* value	Odds Ratio	Combined score
1	MYC	1.350E-02	6.630E-02	7.48	32.21
2	UBTF	3.305E-02	6.630E-02	33.22	113.25
3	KDM5B	3.412E-02	6.630E-02	32.14	108.57
4	CCNE1	4.420E-02	6.630E-02	24.58	76.66
5	FOXP3	1.766E-01	2.119E-01	5.63	9.76
6	ESR1	3.873E-01	3.873E-01	2.20	2.08

Supplementary Table S3

Loewe synergy scores for tagtociclib combination therapies.

Cell line	**Loewe Synergy Score**			
	Cell proliferation		Cell viability	
	Tagtociclib + KB-0742	Tagtociclib + AZD4573	Tagtociclib + KB-0742	Tagtociclib + AZD4573
CIC01	**15.2**	**22.4**	-3.0	-9.0
CIC02	**12.1**	**13.3**	-1.3	**11.5**
CDS1	7.1	**13.6**	**12.0**	**24.2**
CDS2	**14.1**	**19.3**	**17.6**	**29.4**

Bold indicates synergistic effect

Supplementary Table S4

Antibodies used for immunohistochemistry and immunoblotting.

Antibody	Vendor, Catalog Number	Dilution	Application
Cleaved Caspase 3 (Asp175)	Cell Signaling Technology, 9664	1:1000	IHC
Cleaved PARP (Asp214)	Cell Signaling Technology, 5625	1:50	IHC
ETV4	Santa Cruz Biotechnology, sc-113	1:40	IHC
Ki67	Agilent, GA626	1:400	IHC
Ki67	Biocare, PRM325	-	IHC
NUT	Cell Signaling Technology, 3625	1:250	IHC
WT1	Agilent, IR05561-2	1:75	IHC
CDK2	Cell Signaling Technology, 2546	1:1000	IB
Cleaved Caspase 3 (Asp175)	Cell Signaling Technology, 9661	1:1000	IB
Cyclin E	Santa Cruz Biotechnology, sc-247	1:500	IB
DUX4	Abcam, 124699	1:500	IB
GAPDH	Sigma, G8795	1:5000	IB
MCL-1	Cell Signaling Technology, 4572	1:1000	IB
MYC	Cell Signaling Technology, 5605	1:1000	IB
NUT	Cell Signaling Technology, 3625	1:1000	IB
pMYC Ser62	Cell Signaling Technology, 13748	1:1000	IB
pRb Ser795	Cell Signaling Technology, 9301	1:1000	IB
pRPB1-CTD Ser2	Cell Signaling Technology, 13499	1:1000	IB
Rb	Cell Signaling Technology, 9309	1:1000	IB

IHC: immunohistochemistry; IB: immunoblotting
