# Supplementary material for: Co-targeting CDK2 and CDK9 inhibits MYC oncogenic programs and has anti-tumor effects in CIC-rearranged sarcoma
Source: Commun Med (Lond). 2026 Oct 7;6:572. doi: 10.1038/s43856-026-01956-1 (PMC13728178; doi:10.1038/s43856-026-01956-1)
Supplement: Supplementary file 3 [file 43856_2026_1956_MOESM5_ESM.pdf]

NOTE: Please save this file locally before filling in the table, DO NOT work on the file within your internet browser as changes will not be saved. Adobe Acrobat Reader (available free here) is recommended for completion.

The ARRIVE guidelines 2.0: author checklist

The ARRIVE Essential 10

These items are the basic minimum to include in a manuscript. Without this information, readers and reviewers cannot assess the reliability of the findings.

Item		Recommendation	Section/line number, or reason for not reporting
**Study design**	1	For each experiment, provide brief details of study design including:	
		a. The groups being compared, including control groups. If no control group has been used, the rationale should be stated.	Methods, "Xenograft models"; Results, xenograft efficacy studies
		b. The experimental unit (e.g. a single animal, litter, or cage of animals).	Methods, "Xenograft models"; Results, xenograft efficacy studies
**Sample size**	2	a. Specify the exact number of experimental units allocated to each group, and the total number in each experiment. Also indicate the total number of animals used.	Methods, "Xenograft models"; figure legends for Figs. 3 and 6
		b. Explain how the sample size was decided. Provide details of any *a priori* sample size calculation, if done.	No formal a priori sample size calculation was performed; at least three animals per group were planned
**Inclusion and exclusion criteria**	3	a. Describe any criteria used for including and excluding animals (or experimental units) during the experiment, and data points during the analysis. Specify if these criteria were established *a priori*. If no criteria were set, state this explicitly.	Inclusion criteria: Animals with growing tumors
		b. For each experimental group, report any animals, experimental units or data points not included in the analysis and explain why. If there were no exclusions, state so.	No exclusions
		c. For each analysis, report the exact value of *n* in each experimental group.	N is provided in the figure legends
**Randomisation**	4	a. State whether randomisation was used to allocate experimental units to control and treatment groups. If done, provide the method used to generate the randomisation sequence.	Methods, "Xenograft models" (random allocation using Studylog software)
		b. Describe the strategy used to minimise potential confounders such as the order of treatments and measurements, or animal/cage location. If confounders were not controlled, state this explicitly.	Each treatment groups' animals were separated by labeled cages and the order of treatment was the same throughout the study
**Blinding**	5	Describe who was aware of the group allocation at the different stages of the experiment (during the allocation, the conduct of the experiment, the outcome assessment, and the data analysis).	The study was not blinded but the animal technician was agnostic to the outcome of the study
**Outcome measures**	6	a. Clearly define all outcome measures assessed (e.g. cell death, molecular markers, or behavioural changes).	Methods, "Xenograft models"; tumor volume and histopathologic analyses
		b. For hypothesis-testing studies, specify the primary outcome measure, i.e. the outcome measure that was used to determine the sample size.	Tumor volume was the primary outcome measure. No formal a priori sample size calculation was performed; at least 3 animals per group were planned.
**Statistical methods**	7	a. Provide details of the statistical methods used for each analysis, including software used.	Methods, "Statistics and reproducibility"; relevant figure legends
		b. Describe any methods used to assess whether the data met the assumptions of the statistical approach, and what was done if the assumptions were not met.	Methods, "Statistics and reproducibility"; relevant figure legends; no formal assessment of statistical assumptions was performed.
**Experimental animals**	8	a. Provide species-appropriate details of the animals used, including species, strain and substrain, sex, age or developmental stage, and, if relevant, weight.	Abstract; Methods, "Xenograft models"
		b. Provide further relevant information on the provenance of animals, health/immune status, genetic modification status, genotype, and any previous procedures.	Abstract; Methods, "Xenograft models"
**Experimental procedures**	9	For each experimental group, including controls, describe the procedures in enough detail to allow others to replicate them, including:	
		a. What was done, how it was done and what was used.	Methods, "Xenograft models"
		b. When and how often.	Methods, "Xenograft models"
		c. Where (including detail of any acclimatisation periods).	Methods, "Xenograft models"
		d. Why (provide rationale for procedures).	Methods, "Xenograft models"
**Results**	10	For each experiment conducted, including independent replications, report:	
		a. Summary/descriptive statistics for each experimental group, with a measure of variability where applicable (e.g. mean and SD, or median and range).	Results, CIC01 and CIC02 xenograft studies; Figs. 3 and 6
		b. If applicable, the effect size with a confidence interval.	Results, CIC01 and CIC02 xenograft studies; Figs. 3 and 6

The Recommended Set

These items complement the Essential 10 and add important context to the study. Reporting the items in both sets represents best practice.

Item		Recommendation	Section/line number, or reason for not reporting
**Abstract**	11	Provide an accurate summary of the research objectives, animal species, strain and sex, key methods, principal findings, and study conclusions.	Abstract
**Background**	12	a. Include sufficient scientific background to understand the rationale and context for the study, and explain the experimental approach.	Introduction
		b. Explain how the animal species and model used address the scientific objectives and, where appropriate, the relevance to human biology.	Immunodeficient mice were used to establish and propagate patient-derived xenograft models of human CIC-rearranged sarcoma, providing an in vivo model relevant to human tumor biology
**Objectives**	13	Clearly describe the research question, research objectives and, where appropriate, specific hypotheses being tested.	Introduction
**Ethical statement**	14	Provide the name of the ethical review committee or equivalent that has approved the use of animals in this study, and any relevant licence or protocol numbers (if applicable). If ethical approval was not sought or granted, provide a justification.	Methods, "Human and animal ethics statement"
**Housing and husbandry**	15	Provide details of housing and husbandry conditions, including any environmental enrichment.	Methods, "Xenograft models"
**Animal care and monitoring**	16	a. Describe any interventions or steps taken in the experimental protocols to reduce pain, suffering and distress.	Methods, "Xenograft models"
		b. Report any expected or unexpected adverse events.	Methods, "Xenograft models"
		c. Describe the humane endpoints established for the study, the signs that were monitored and the frequency of monitoring. If the study did not have humane endpoints, state this.	Methods, "Xenograft models"
**Interpretation/ scientific implications**	17	a. Interpret the results, taking into account the study objectives and hypotheses, current theory and other relevant studies in the literature.	Discussion
		b. Comment on the study limitations including potential sources of bias, limitations of the animal model, and imprecision associated with the results.	Discussion
**Generalisability/ translation**	18	Comment on whether, and how, the findings of this study are likely to generalise to other species or experimental conditions, including any relevance to human biology (where appropriate).	Discussion
**Protocol registration**	19	Provide a statement indicating whether a protocol (including the research question, key design features, and analysis plan) was prepared before the study, and if and where this protocol was registered.	The study protocol was not preregistered
**Data access**	20	Provide a statement describing if and where study data are available.	Data Availability
**Declaration of interests**	21	a. Declare any potential conflicts of interest, including financial and non-financial. If none exist, this should be stated.	Competing Interests; Acknowledgements/Funding
		b. List all funding sources (including grant identifier) and the role of the funder(s) in the design, analysis and reporting of the study.	Funding
