# Supplementary material for: Co-targeting CDK2 and CDK9 inhibits MYC oncogenic programs and has anti-tumor effects in CIC-rearranged sarcoma
Source: Commun Med (Lond). 2026 Oct 7;6:572. doi: 10.1038/s43856-026-01956-1 (PMC13728178; doi:10.1038/s43856-026-01956-1)
Supplement: Supplementary file 4 [file 43856_2026_1956_MOESM3_ESM.docx]

**Description of Additional Supplementary Files**

Supplementary Data 1- Raw numerical data underlying the graphs in Figures 3-6 and Supplementary Figures S4, S8 and S10
